# A Computational Model of Hepatic Energy Metabolism: Understanding Zonated Damage and Steatosis in NAFLD

**DOI:** 10.1371/journal.pcbi.1005105

**Published:** 2016-09-15

**Authors:** William B. Ashworth, Nathan A. Davies, I. David L. Bogle

**Affiliations:** 1 Institute of Liver and Digestive Health, University College London, London, United Kingdom; 2 Department of Chemical Engineering, University College London, London, United Kingdom; 3 CoMPLEX, University College London, London, United Kingdom; University of Michigan, UNITED STATES

## Abstract

In non-alcoholic fatty liver disease (NAFLD), lipid build-up and the resulting damage is known to occur more severely in pericentral cells. Due to the complexity of studying individual regions of the sinusoid, the causes of this zone specificity and its implications on treatment are largely ignored. In this study, a computational model of liver glucose and lipid metabolism is presented which treats the sinusoid as the repeating unit of the liver rather than the single hepatocyte. This allows for inclusion of zonated enzyme expression by splitting the sinusoid into periportal to pericentral compartments. By simulating insulin resistance (IR) and high intake diets leading to the development of steatosis in the model, we identify key differences between periportal and pericentral cells accounting for higher susceptibility to pericentral steatosis. Secondly, variation between individuals is seen in both susceptibility to steatosis and in its development across the sinusoid. Around 25% of obese individuals do not show excess liver fat, whilst 16% of lean individuals develop NAFLD. Furthermore, whilst pericentral cells tend to show higher lipid levels, variation is seen in the predominant location of steatosis from pericentral to pan-sinusoidal or azonal. Sensitivity analysis was used to identify the processes which have the largest effect on both total hepatic triglyceride levels and on the sinusoidal location of steatosis. As is seen *in vivo*, steatosis occurs when simulating IR in the model, predominantly due to increased uptake, along with an increase in *de novo* lipogenesis. Additionally, concentrations of glucose intermediates including glycerol-3-phosphate increased when simulating IR due to inhibited glycogen synthesis. Several differences between zones contributed to a higher susceptibility to steatosis in pericentral cells in the model simulations. Firstly, the periportal zonation of both glycogen synthase and the oxidative phosphorylation enzymes meant that the build-up of glucose intermediates was less severe in the periportal hepatocyte compartments. Secondly, the periportal zonation of the enzymes mediating β-oxidation and oxidative phosphorylation resulted in excess fats being metabolised more rapidly in the periportal hepatocyte compartments. Finally, the pericentral expression of *de novo* lipogenesis contributed to pericentral steatosis when additionally simulating the increase in sterol-regulatory element binding protein 1c (SREBP-1c) seen in NAFLD patients *in vivo*. The hepatic triglyceride concentration was predicted to be most sensitive to inter-individual variation in the activity of enzymes which, either directly or indirectly, determine the rate of free fatty acid (FFA) oxidation. The concentration was most strongly dependent on the rate constants for β-oxidation and oxidative phosphorylation. It also showed moderate sensitivity to the rate constants for processes which alter the allosteric inhibition of β-oxidation by acetyl-CoA. The predominant sinusoidal location of steatosis meanwhile was most sensitive variations in the zonation of proteins mediating FFA uptake or triglyceride release as very low density lipoproteins (VLDL). Neither the total hepatic concentration nor the location of steatosis showed strong sensitivity to variations in the lipogenic rate constants.

## Author Summary

Fat build up in liver is known to increase the likelihood of developing numerous health problems around the body including cardiovascular problems, desensitisation to the hormone insulin leading to type 2 diabetes, and the development of fibrous tissue in liver (fibrosis) resulting in loss of liver function (cirrhosis). Liver cells show marked differences in their metabolism depending upon their position along liver capillaries (sinusoids). It has been shown previously that cells nearer the output end of the sinusoid (pericentral) are more susceptible to excess fat and the resulting damage than those near the input end. However, due to the micro-meter scale of the sinusoids and the large number of potential variables, it is difficult to study metabolism in individual regions of the sinusoid experimentally when considering various feeding and disease states. Here, a computational model of sinusoidal metabolism incorporating previously measured differences in enzyme activities across the sinusoid is presented and used to assess the key metabolic differences leading to pericentral susceptibility to excess fat build-up. Secondly, the model is used to assess the metabolic variations between individuals most likely to account for inter-individual variability in susceptibility to excess liver fat and its predominant location. These simulations will aid understanding of disease progression and allow for more targeted future experimental work.

## Introduction

Non-alcoholic fatty liver disease (NAFLD) is the term given to the build-up of excess fats in liver cells when other causes have been ruled out. Excessive fat is thought to be present in the livers of 25–30% of the UK population [3] with similar numbers seen across Europe and the USA [4, 5].

NAFLD is strongly linked with IR and type 2 diabetes mellitus (T2DM) [6–9]. IR leads to increased plasma free fatty acid (FFA) and triglyceride concentrations resulting in increased lipid uptake into hepatocytes [10–13]. Additionally, FFAs in liver and muscle are known to reduce insulin sensitivity both in the cells themselves and peripherally [14]. Therefore, a feedback cycle exists in which IR causes lipid build-up in liver and muscle, which in turn cause further IR. Numerous other factors are linked with the development of NAFLD including poor diet, obesity and high blood pressure [3, 8, 9]. As of 2014, 3.2 million people in the UK, 32 million people in Europe and 29.1 million people in the USA were estimated to suffer from diabetes. Additionally, the number of people showing signs of ‘prediabetes’ increased from 10% to 33% in the UK between 1996 and 2011, and from 79 million to 86 million in the USA between 2010 and 2012 [15–19]. As a result, the number of deaths associated with diabetes and the cost of its treatment are expected to rapidly increase in the coming decades and it is of great importance to focus on prevention and tackling the disease in its early stages.

As well as contributing to the development of T2DM, the build-up of fat can cause inflammation and liver damage in a condition known as non-alcoholic steatohepatitis (NASH). It is estimated that 2–5% of the UK population suffer from NASH, which is associated with development of fibrosis in 40–50% of patients [20], cirrhosis in 20% and liver-related death in 12% [3, 21]. It has been suggested that NASH may also be responsible for many cases in which cirrhosis occurs with unknown causes [22, 23]. By 2030 it has been predicted that fatty liver disease will become the largest cause of liver transplant [24]. Fat build-up in liver has also been shown to promote increased mortality due to causes outside of the liver, including cardiovascular disease [24].

In adult sufferers of NAFLD, steatosis is often most severe in pericentral cells [25–27]. When assessed by Chalasani *et al*., 38% of subjects showed steatosis predominantly in pericentral cells, 62% showed azonal or pan-sinusoid steatosis and less than 1% showed steatosis restricted to periportal cells [25]. Additionally, inflammation and fibrosis tend to be more severe towards the pericentral end of the sinusoid [25–28]. When considering the effects of IR, this pattern of lipid build-up could be considered counter intuitive. Pericentral cells specialise in lipogenesis, whilst periportal cells show increased expression of fatty acid (FA) uptake proteins. Given the context of reduced insulin stimulation of lipogenesis but raised plasma lipid levels and increased hepatic FFA uptake, it would be expected that periportal cells to show the most severe steatosis [29, 30].

A potential solution to this apparent paradox suggested in the literature is that IR could be restricted to glucose metabolism, with insulin signalling remaining intact in lipid metabolism [29, 31]. Consistent with this, increased rates of lipogenesis and triglyceride synthesis are seen in insulin resistant NAFLD patients. Additionally, the expression of the lipid metabolism regulatory protein sterol-regulatory element binding protein 1c (SREBP-1c), which is stimulated by insulin under metabolically normal conditions, is increased in insulin resistant NAFLD livers [1, 2]. Furthermore, increased expression of carbohydrate responsive element binding protein (ChREBP), an additional transcription factor whose expression is primarily regulated by the presence of sugars rather than insulin, is seen in NAFLD as a result of hyperglycaemia [32, 33]. However, whilst hepatic lipogenesis is increased in NAFLD, it is known that the majority of the lipids in NAFLD arise from uptake, such that an explanation of pericentral-centered steatosis should not be based primarily on increased hepatic lipid production [30, 34–36]. Additionally, separate pathways for insulin reception required for a duel resistance/sensitivity effect have not been identified, and it is known that the two insulin receptors (IRS-1 and IRS-2) both act on glucose and lipid metabolisms [37–40]. The link between IR and the susceptibility of pericentral cells to steatosis, therefore, remains unexplained.

Fully characterising the development and consequences of metabolic diseases such as NAFLD in cells across the sinusoid experimentally would be a challenging task due to the small size of the sinusoid and the large number of potential variables. In this study, we present a computational model of hepatic glucose, lipid and energy metabolism across the sinusoid, integrating existing knowledge of hepatic enzyme expression, substrate dependences, regulation and zonation.

The model was used to study the development of NAFLD, with a particular focus on the causes of pericentral susceptibility to steatosis. We first assessed whether the model reproduces the changes to hepatic lipid levels, glucose storage and energy metabolism seen in steatotic livers *in vivo* when simulating IR. Additionally, the contributions of increased SREBP-1c expression and increased dietary intake to these changes in metabolism were assessed.

Having validated that the model outputs are consistent with *in vivo* observations for NAFLD, we next investigated what causes cells at the pericentral end of the sinusoid to be more susceptible to steatosis than periportal cells. Using the computational model, we are able to make detailed predictions of the changes in the conversion rates and metabolite concentrations across the sinusoid under different feeding conditions for the healthy and disease states.

Finally, inter-individual variation is seen both in susceptibility to steatosis and in the pattern of steatotic build-up. Around 25% of obese individuals fail to develop steatosis whilst 16% of lean individuals show excess liver fat [8], and variation is seen in the predominant location of steatosis from pericentral to azonal or pan-sinusoidal [25–27]. We wish to identify variations in hepatic metabolism likely to account for these differences. Focussing on hepatic processes, sensitivity analysis was performed on the rate and zonation constants in the model to identify those with the largest effects on hepatic triglyceride and FFA levels.

### Existing Models of Metabolism and Zonation

Previous computational models of glucose homeostasis and liver energy metabolism have varied from those studying liver metabolism within the body as a whole to those focussed in detail on the regulation of specific enzymes depending upon the particular purpose of the study. For example, Kim *et al*. [41] and Xu *et al*. [42] developed whole body models to study the hormonal regulation of glucose homeostasis during exercise (Kim *et al*. [41]) and under varying feeding conditions (Xu *et al*. [42]), whilst Liu *et al*. [43] developed a model focussed in detail on the GLUT proteins responsible for glucose uptake and output. Additionally, representations of glucose regulation vary from black box models, for example with the purpose of calculating optimal insulin input for insulin responsive diabetic patients [44], to mechanistic models aiming to understand the metabolic changes occurring in disease in detail [45–50]. Of these mechanistic models, the vast majority of existing models of the liver are single hepatic compartment models, and zonation in the context of hepatic energy metabolism has yet to be addressed.

Numerous models of glucose regulation and other processes in liver have been presented since as early as 1965 [44]. As a result, the following review will focus on key recent mechanistic models. For a more detailed review of liver metabolism and diabetes modelling, see the reviews of Bogle *et al*. and Balakrishnan *et al*. [44, 51].

Most recently, Somvanshi *et al*. published a detailed single compartment model of glucose, lipid and amino acid metabolism, including regulation at both a signalling and transcription level [45]. This model, which incorporated a number of previously published sub-models, was used to understand the effects of varying dietary compositions on metabolism. König *et al*. developed a detailed model of enzymatic conversions in glucose homeostasis including much of the allosteric regulation known to occur in glucose regulation [46]. This model (a set of ordinary differential equations with 49 localised metabolites and 36 reactions) included all of the enzymatic conversions involved in gluconeogenesis and glycolysis, with separate cytoplasmic and mitochondrial compartments within hepatocytes. Hetherington *et al*. developed a composite model of hormone signalling in glycogen storage, comprising of established and *ab initio* developed sub-models [47]. This was used to understand key features of insulin signalling including ultradian oscillations [47, 48]. Chalhoub *et al*. developed a single hepatic compartment model focussing on gluconeogenesis and lipid metabolism in the hepatocyte [49]. The model was adapted to give results corresponding to the liver in either *in vivo* or in an *ex vivo* perfusion system to allow comparison with different experimental data. It was then used to simulate concentrations of various molecules and fluxes of different reactions in response to changes in the composition of the perfusion medium. Calvetti *et al*. developed a sophisticated spatially distributed model of glucose regulation in liver [50]. This used a set of grid points to measure fluxes of various molecules. However, despite the inclusion of spatial distribution, this model did not include zonation in enzyme expression.

Whilst single compartment, homogenous hepatocyte models are useful for studying the function of liver as a whole, they do not allow simulation of changes within specific regions of the sinusoid, and exclude the implications of zonation in disease progression. Only a few models have included a representation of hepatic zonation in enzyme expression, and none of these have focused on liver energy metabolism. Ohno *et al*. investigated heterogeneity in ammonia detoxification [52]. They set up a model in which substances enter the sinusoids from the periportal tract, pass through 8 compartments (hepatocytes) in series, before exiting the sinusoid in the central vein. The model was tested with homogenous enzyme expression in all compartments and with zonated expression of carbonyl phosphate synthase, glutamine synthase and ornithine aminotransferase to understand the roles of zonated enzyme expression. Anissimov *et al*. created a similar model with 8 compartments to study hepatic availability and clearance [53]. A model by Sheikh-Bahaei *et al*. also studied hepatic zonation xenobiotic toxicity focussing on the development of zonation in key enzymes, rather than the effects of this zonation on metabolite concentrations [54]. Pang et al. looked at the various different ways of modelling heterogeneity for studying pharmacokinetics, starting with a simple compartmental PBPK model, followed by a zonal model and moving on to more complex circulatory and fractal models [55]. The compartmental and zonal models were compared with clinical data for digoxin and estradiol 17β D-glucuronide (E_2_17G). For digoxin, the zonal model provided little improvement over the compartmental model. However, for E_2_17G results improved significantly if Sult1e1 was expressed heterogeneously, suggesting that zonation is a more important for some drugs than others.

Other than this, models have been developed including zonation of closely related non-liver metabolism. König *et al*. published a model of glucose metabolism in cancel cells including localized gradients in metabolites and oxygen and regional hypoxia across a tumour [56]. Although this is for metabolism in cancer cells rather than liver, many similarities are seen including increased glycolysis and reduced oxidative phosphorylation in the most hypoxic cells. Davidson *et al*. studied the development of zonation within a bioartificial device [57]. This study did not model the metabolism in cells but instead focused on optimisation of oxygen input, blood flow and the dimensions of the device to try set-up a liver-like gradient in oxygen expression across the cells to promote a zonated phenotype.

## Methods

### Model Development

Due to the size of the model, a full description of the metabolic processes and the equations used to represent them is provided in S1 Text. A summary is presented in the following sections. Quantitative comparison of the simulated data for the concentrations of the various plasma and hepatic molecules in both metabolically healthy (MH) and insulin resistant individuals with experimental data is provided in S2 Text.

### Model Structure

**Fig 1:**
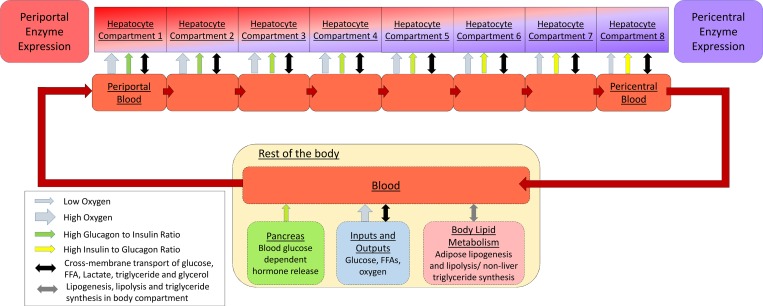
The structure of the model. The porto-central axis of the sinusoid is considered to be the repeating unit of the liver. Cells and blood are compartmentalized into groups according to their position along the liver sinusoid. This allows the model to include changes in blood oxygenation, hormone concentrations and substrate and product concentrations across the sinusoid as well as differences in hepatic enzyme expression between compartments. Blood exits the sinusoid into a larger compartment representing the rest of the body. Blood in this compartment interacts with the pancreas, lungs and adipose tissue. Glucose and FFA inputs and consumption also occur in this compartment.

To include zonation, a computational model of liver function must be able to represent changes in concentrations of metabolites and hormones as blood passes through the sinusoid, as well as the variation in sinusoidal hepatic enzyme expression. Conventional two compartment (blood/hepatocyte) models, which treat the hepatocyte as the repeating unit of the liver, are unable to do this. Instead, following the structure suggested by Anissimov et al. [53] and Ohno *et al*. [52], we treat the porto-central axis of the sinusoid as the repeating unit. The blood and surrounding hepatocytes in the sinusoid are split into compartments according to their position along this axis (proximal periportal -> distal pericentral) (Fig 1).

8 compartments were used when simulating the data in this article to allow simple comparison with experimental studies which tend to split the sinusoid into 2–4 zones, up to a maximum of 8 (e.g. [58]). This is also consistent with previous computational models of zonation in liver [52, 53]. No under-sampling effects were noted when using 8 compartments compared with simulations run using higher compartment numbers.

Blood flows from the periportal to the pericentral end of the sinusoid. After leaving the distal pericentral compartment, it enters a larger body compartment where it interacts with simple representations of the pancreas (hormone input), adipose tissue (FFA and triglyceride regulation) and with glucose and FFA inputs/outputs in the rest of the body (Fig 1). The model simulates an individual at rest and does not include the blood flow, blood oxygenation and hormonal changes occurring during exercise.

Although this representation of the sinusoid allows inclusion of zonated enzyme expression, it remains a simplification. Several sinusoids extend between each portal triad and central vein passing following indirect paths through the cells, and the number of cells fed by each sinusoid will vary. Due to the hexagonal shape of the lobule, each sinusoid is likely to be supplying a larger number of cells nearer the portal triad than the central vein. Additionally, given that oxygen concentration is the signal promoting zonated expression [59], cells further from the capillary (but in the outer periportal region of the lobule) are likely to show more pericentral expression than those neighbouring the capillary. Therefore, there is scope for development of models to refine predictions in the future by representing distributed effects across 2D and 3D representations of the lobule.

### Hepatic Metabolism

**Fig 2:**
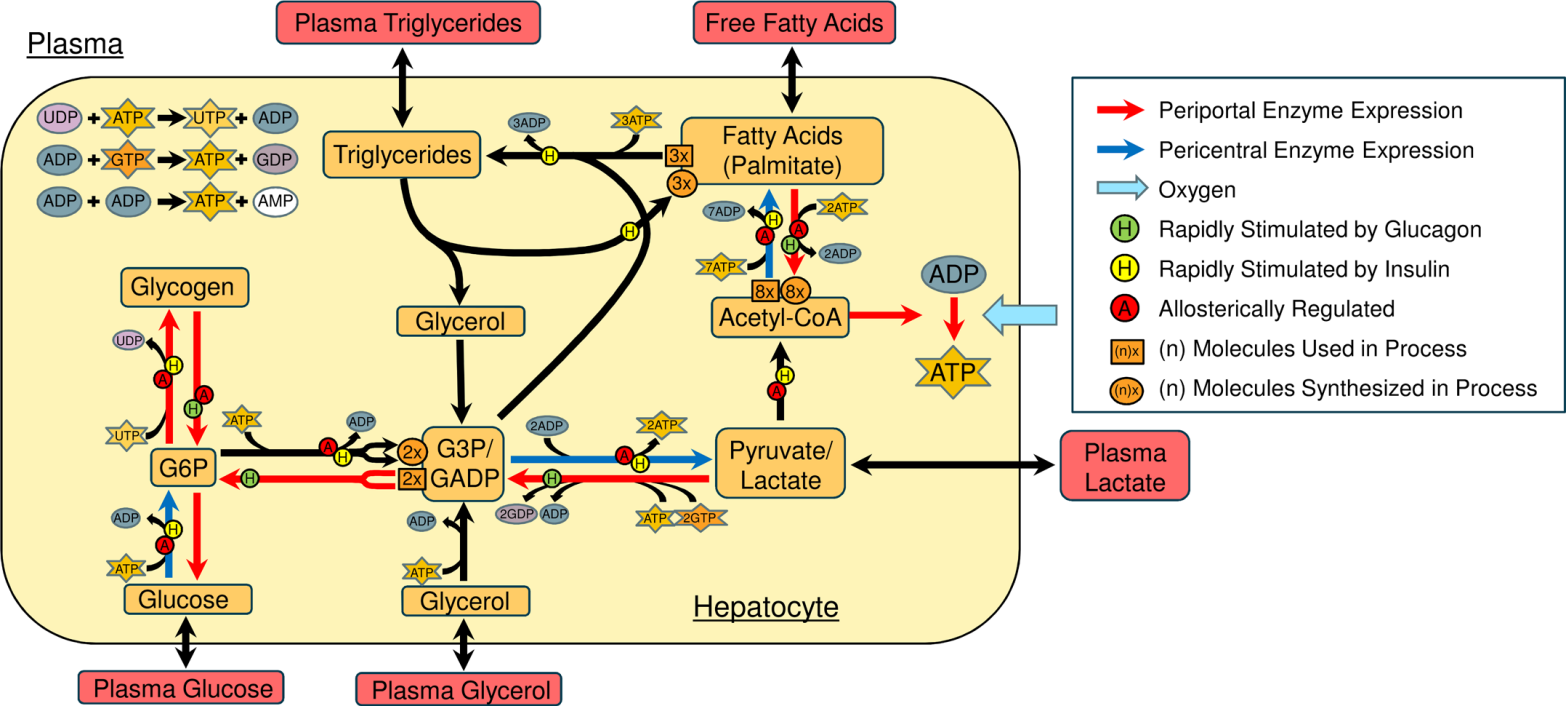
Variables and conversions included in each hepatic compartment. In addition to the hormonal regulation, almost all of the glucose and lipid metabolism conversions show some form of allosteric regulation. Lipolysis is also included but it occurs at very slow rate in hepatocytes.

Fig 2 shows the variables and processes included in each hepatic compartment of the model. The model focusses on the storage of glucose as glycogen, the cycling between glucose and lactate, adenosine triphosphate (ATP) production, FA production and the storage of FFAs as triglycerides. A reduced description of the representation of metabolism in each hepatic compartment in the model is provided in Tables 1 and 2. Table 1 contains the differential equations for each hepatic variable in terms of the metabolic processes. Table 2 defines the metabolic processes included in the model and references the sections of the supplementary material in which the full equations can be found. A detailed description of all equations in the model, along with the values of each constant and references used to set them is provided in S1 Text.

**Table 1:** The rate equations for the variables included in the hepatic compartments of the model.

Hepatic Variable	Rate Equations
Glucose	$\frac{dG_{c}}{dt}=-R_{GK}+R_{G6Pase}{+R}_{Glut}$
G6P	$\frac{dG6P}{dt}=R_{GK}-R_{G6Pase}{+R}_{GP}{-R}_{GS}+R_{FBP}{-R}_{PFK}$
Glycogen	$\frac{dGly}{dt}=R_{GS}-R_{GP}$
G3P/GADP	$\frac{dG3P}{dt}=R_{PEPCK}-2\;R_{FBP}+2\;R_{PFK}-R_{PK}+R_{GConv}-R_{TSyn}$
Pyruvate/Lactate	$\frac{d{Lac}_{c}}{dt}=R_{Lact}{-R}_{PEPCK}{+R}_{PK}-R_{PDH}$
Acetyl-CoA	$\frac{dACoA}{dt}=R_{PDH}-{8\;R}_{Lgen}+8\;R_{\beta oxi}-R_{ATPS}$
FFA (Palmitate)	$\frac{d{FFA}_{c}}{dt}=R_{FFAt}+3\;R_{Lply}-3\;R_{TSyn}+{R_{Lgen}-R}_{\beta oxi}$
Triglycerides	$\frac{d{TG}_{c}}{dt}=R_{TGt}+R_{TSyn}-R_{Lply}$
Glycerol	$\frac{d{Gly}_{c}}{dt}=R_{Glyt}-R_{GConv}+R_{Lply}$
ATP—Adenosine Triphosphate	$\frac{dATP}{dt}=12\;R_{ATPS}-R_{AK}-2\;R_{PEPCK}{-R}_{PFK}+3.25\;R_{PK}{-R}_{GK}-R_{NDKG}{-R}_{NDKU}-R_{ATPu}-2\;R_{\beta oxi}-{7\;R}_{Lgen}-R_{GConv}+{2.5\;R}_{PDH}-3\;R_{TSyn}$
ADP—Adenosine Diphosphate	$\frac{dADP}{dt}=-12\;R_{ATPS}+{2\;R}_{AK}+2\;R_{PEPCK}{+R}_{PFK}-3.25\;R_{PK}+R_{GK}+R_{NDKG}{+R}_{NDKU}+R_{ATPu}+{7\;R}_{Lgen}+R_{GConv}+R_{\beta oxi}-{2.5\;R}_{PDH}$
AMP—Adenosine Monophosphate	$\frac{dAMP}{dt}={-R}_{AK}+R_{\beta oxi}+3\;R_{TSyn}$
Pi—Inorganic Phosphate	$\frac{dPi}{dt}=-12\;R_{ATPS}{-R}_{GP}+R_{G6Pase}{+2\;R}_{GS}{-2.25\;R}_{PK}+2\;R_{PEPCK}+R_{FBP}-R_{PReg}{+R}_{ATPu}+7{\;R}_{Lgen}+R_{\beta oxi}{-2.5\;R}_{PDH}+7\;R_{TSyn}$
UTP—Uridine Triphosphate	$\frac{dUTP}{dt}=R_{NDKU}{-R}_{GS}$
UDP—Uridine Diphosphate	$\frac{dUDP}{dt}=-R_{NDKU}{+R}_{GS}$
GTP—Guanosine Triphosphate	$\frac{dGTP}{dt}=R_{NDKG}-R_{PEPCK}$
GDP—Guanosine Diphosphate	$\frac{dGDP}{dt}=-R_{NDKG}+R_{PEPCK}$

The rates/processes (R) are defined in Table 2. References to the specific sections of the supplementary material in which the full equation for each process can be found are also provided in Table 2. A full description of the model is provided in S1 Text.

**Table 2:** The processes included in the model.

Process	Conversion
$R_{Glut}$–Glucose Uptake.	• $G_{B}\begin{array}{c} ⟶\\ ⟷ \end{array}G_{C}$ (Blood Glucose $\begin{array}{c} ⟶\\ ⟷ \end{array}$ Cellular Glucose) • Periportal to pericentral ratio in rate constant– 1:1 • *S1 Text–Sect. ‘Glucose Uptake by GLUT2’*
$R_{GK}$–Glucokinase.	• *G*_*C*_ + *ATP* → *G*6*P* + *ADP* • Periportal to pericentral ratio in rate constant– 1:2.5 • *S1 Text–Sect. ‘Glucokinase’*
$R_{G6Pase}$–Glucose-6-Phosphatase.	• *G*6*P* → *G*_*C*_ + *P*_*i*_ • Periportal to pericentral ratio in rate constant– 1.9:1 • *S1 Text–Sect. ‘G6Pase’*
$R_{GS}$–Glycogen Synthesis.	• *G*6*P* + *UTP* → *Glycogen* + *UDP* + 2 *P*_*i*_ • Periportal to pericentral ratio in rate constant– 3:1 • *S1 Text–Sect. ‘Glycogen Synthase’*
$R_{GP}$–Glycogen Phosphorylation.	• *Glycogen* + *P*_*i*_ → *G*6*P* • Periportal to pericentral ratio in rate constant– 1:1 • *S1 Text–Sect. ‘Glycogen Phosphorylase’*
$R_{PFK}$–Glycolysis stage 1, primarily rate-limited by phosphofructokinase.	• *G*6*P* + *ATP* → 2 *GADP*/*G*3*P* + *ADP* • Periportal to pericentral ratio in rate constant– 1:1 • *S1 Text–Sect. ‘Glycolysis 1: G6P to GADP’*
$R_{PK}$–Glycolysis stage 2, primarily rate-limited by pyruvate kinase.	• *GADP*/*G*3*P* + 2*ATP* + *P*_*i*_ → *Pyr*/*Lac* + 2*ATP* + additional indirect 1.25 ATP from NADH. • Periportal to pericentral ratio in rate constant– 1:2.1 • *S1 Text–Sect. ‘Glycolysis 2: GADP to Lactate/Pyruvate’*
$R_{PEPCK}$–Gluconeogenesis stage 1, primarily rate-limited by phosphoenolpyruvate carboxykinase.	• *Pyr*/*Lac* + 2*ATP* + *GTP* → *GADP*/*G*3*P* + 2*ADP* + *GDP* + 2 *P*_*i*_ • Periportal to pericentral ratio in rate constant– 2.4:1 • *S1 Text—Sect. ‘Gluconeogenesis 1: Lactate/Pyruvate to GADP’*
$R_{FBP}$–Gluconeogenesis 2, primarily rate-limited by fructose-bisphosphatase.	• 2 *GADP*/*G*3*P* → *G*6*P* + *P*_*i*_ • Periportal to pericentral ratio in rate constant– 1.75:1 • *S1 Text–Sect. ‘Gluconeogenesis 2: GADP to G6P’*
$R_{PDH}$–Pyruvate oxidation mediated by pyruvate dehydrogenase.	• *Pyr*/*Lac* → *ACoA* + indirect 2.5 ATP from NADH • Periportal to pericentral ratio in rate constant– 1:1 • *S1 Text–Sect. ‘Pyruvate Oxidation’*
$R_{\beta oxi}$–β-Oxidation.	• *FFA* + 2 *ATP* → 8 *ACoA* + *ADP* + *AMP* + 3 *P*_*i*_ • Periportal to pericentral ratio in rate constant– 1.6:1 • *S1 Text–Sect. ‘β-Oxidation’*
$R_{ATPS}$—ATP Synthesis via the citrate cycle and electron transport chain.	• *ACoA* + 12*ADP* + 12 *P*_*i*_ → 12 *ATP* • Periportal to pericentral ratio in rate constant– 1.5:1 • *S1 Text–Sect. ‘Oxidative Phosphorylation/The Citrate Cycle’*
$R_{NDKG}/R_{NDKU}$–Nucleoside Diphosphate Kinases.	• (NDKG): *GDP* + *ATP* → *GTP* + *ADP* • (NDKU): *UDP* + *ATP* → *UTP* + *ADP* • Periportal to pericentral ratio in rate constant– 1:1 • *S1 Text–Sect. ‘Nucleoside Diphosphate Kinases’*
$R_{AK}$–Adenosine Kinase.	• *ATP* + *AMP* ↔ 2 *ADP* • Periportal to pericentral ratio in rate constant– 1:1 • *S1 Text–Sect. ‘Adenosine Kinase’*
$R_{ATPu}$–Cellular ATP consumption. $R_{PReg}$–Inorganic Phosphate Regulation.	• *ATP* → *ADP* + *P*_*i*_ • *P*_*i*_ ↔ − • Periportal to pericentral ratio in both rate constant– 1:1*S1 Text–Sect. ‘Additional ATP Use’*
$R_{Lgen}$–Lipogenesis.	• *ACoA* + 7 *ATP* → *FFA* + 7 *ADP* + 7 *P*_*i*_ • Periportal to pericentral ratio in rate constant– 1:1.6 • *S1 Text–Sect. ‘Lipogenesis’*
$R_{TSyn}$–Triglyceride Synthesis.	• 3 *FFA* + *G*3*P*/*GADP* + 3 *ATP* → *TG* + 3 *AMP* + 7 *P*_*i*_ • Periportal to pericentral ratio in rate constant– 1:1 • *S1 Text–Sect. ‘Triglyceride Synthesis’*
$R_{Lply}$–Lipolysis.	• *TG* → *Glyc* + 3 *FFA* • Periportal to pericentral ratio in rate constant– 1:1 • *S1 Text–Sect. ‘Lipolysis’*
$R_{GConv}$–Glycerol Kinase.	• *Glyc* + *ATP* → *G*3*P*/*GADP* + *ADP* • Periportal to pericentral ratio in rate constant– 1:1 • *S1 Text–Sect. ‘Glycerol Kinase*
$R_{Lact}$–Lactate Output/Uptake.	• *Lac*_*B*_ ↔ *Lac*_*C*_ • Periportal to pericentral ratio in rate constant– 1:1 • *S1 Text–Sect. ‘Lactate Output/Uptake’*
$R_{FFAt}$–FFA Output/Uptake.	• ${FFA}_{B}\begin{array}{c} ⟷\\ ⟶ \end{array}{FFA}_{C}$ • Periportal to pericentral ratio in rate constant– 1.5:1 • *S1 Text–Sect. ‘Fatty Acid Output/Uptake’*
$R_{TGt}$–Triglyceride Output/Uptake.	• ${TG}_{B}\begin{array}{c} ⟷\\ ⟵ \end{array}{TG}_{C}$ • Periportal to pericentral ratio in rate constant– 1:1 • *S1 Text–Sect. ‘Triglyceride Output/Uptake’*
$R_{Glyt}$–Glycerol Output/Uptake.	• *Glyc*_*B*_ ↔ *Glyc*_*C*_ • Periportal to pericentral ratio in rate constant– 1:1 • *S1 Text–Sect. ‘Glycerol Output/Uptake’*
Hormone Reception	• Periportal to pericentral ratio in insulin reception– 1:1.35 • Periportal to pericentral ratio in glucagon reception– 1.35:1
Non Hepatic Processes	
Adipose Lipogenesis.	• $4\;G_{B}\begin{array}{c} ⟷\\ ⟶ \end{array}{FFA}_{B}$ • *S1 Text–Sect. ‘Adipose De Novo Synthesis’*
Adipose Lipolysis.	• ${TG}_{B}\begin{array}{c} ⟷\\ ⟶ \end{array}3{\;FFA}_{B}+{Glyc}_{B}$ • *S1 Text–Sect. ‘Adipose Lipolysis’*
Body (Predominantly Gut) Triglyceride Synthesis.	• $3\;{FFA}_{B}+{Glyc}_{B}\begin{array}{c} ⟷\\ ⟶ \end{array}{TG}_{B}$ • *S1 Text–Sect. ‘Non-Hepatic Triglyceride Synthesis’*
FFA and Glucose Consumption in Muscle and Other Non-Liver Cells.	• *FFA*_*B*_ → − • *G*_*B*_ → − • *S1 Text–Sect. ‘Consumption Terms’*
Glucose and Hormone Inputs and Degradation/Consumption.	• *Oxygen*,*Glucagon*,*Insulin* ↔ − • *S1 Text–Sect. ‘Oxygen and Hormone Inputs and Consumption’*

Full equations along with the values of constants, the experimental data used as references and a detailed discussion are provided in S1 Text.

### Adipose Tissue, Muscle and Inputs and Outputs

Since the focus of this study is on liver metabolism, the model includes only very simple representations of essential processes occurring elsewhere in the body. The representation of pancreatic hormone release developed by Hetherington *et al*. was used to calculate the rate of release of glucagon and insulin into the blood [47]. The two hormones are then degraded at constant rates (per unit of hormone) as blood passes through the sinusoid to match the experimentally measured changes in concentrations [59, 60].

A constant oxygen input was added to the body compartment along with a constant rate of consumption across the sinusoid. These were set such that the oxygen concentration falls from 65mmHg in the blood entering the proximal periportal compartment to 35mmHg in the blood leaving the distal pericentral compartment [59]. Since no changes in oxygen input or blood flow were simulated in this study, the oxygen concentrations across the sinusoid remained fixed at the experimentally measured gradient for a healthy liver. However, the inclusion of a dynamic oxygen calculation may allow the model to be used to study changes in oxygen supply and blood flow in developing liver disease in the future. The rate of oxidative phosphorylation is oxygen dependent in the model, with a K_M_ value based on the measurements by Matsumura *et al*. [61].

In addition to the liver, organs including the intestine, adipose tissue and muscle play important roles in FFA and glucose metabolism and consumption. Since the focus of this study is on liver metabolism, rather than including separate compartments with complex sets of pathways to represent these, single equation calculations for each key process dependent upon the plasma substrate and hormone concentrations were used. These retain the ability to calculate acceptable concentrations for the various plasma molecules entering the sinusoid in both metabolically healthy and insulin resistant individuals as discussed in S1 Text. The processes represented in the body compartment are adipose tissue FFA synthesis, adipose lipolysis, triglyceride production in organs other than liver (primarily intestine), and glucose and FFA uptake by muscle and other body cells.

### Modelling and Parameterisation Strategy

#### Level of detail

The model is limited to the major processes determining triglyceride levels, ATP concentrations and fatty acid oxidation rates in liver, calculating the overall rates of these processes rather than for each intermediate enzyme. A larger model may, for example, include cholesterol synthesis, include detailed adipose metabolism or individually simulate every enzyme mediating each process. Whilst these extra components would allow the model to simulate additional features of metabolism, for example regarding plasma cholesterol or hepatic intermediate concentrations, it is not expected that they would alter the data presented in this report which were focussed towards a specific aim of understanding lipid build-up across the sinusoid. Conversely, given that each of the processes included in the model are fundamental to energy metabolism and regulation across the sinusoid, exclusion of any of the processes in the model would be expected to significantly alter the observations presented in this report.

#### Representation of each process

For each conversion, a search of the literature, along with online databases such as BRENDA [62], was first performed to establish allosteric and hormone dependences of the key enzymes mediating the process. The allosteric dependences of each process on other molecules in the model were included in the form of hill functions with constants taken directly from the literature. The dependences on the various energy molecules in the model (ATP, GTP, UTP *etc*.) were also included as hill functions with constants taken from the literature for each process. Where possible, the hill function dependences on the various metabolic intermediates acting as substrates for each process were also taken directly from published experimental data. However, for some processes, e.g. glycolysis and gluconeogenesis, the rate limiting enzyme is not the first in the chain. As a result, in these cases, the substrate dependences were fitted by eye to experimental data from a number of sources (S1 Text). For each process, the rate constant and constants mediating the insulin and glucagon dependences were also fitted to experimental data.

Since the exact rates at which glucose and lipids enter the bloodstream after feeding in experimental studies are unknown, automated least-squares fitting parameters to any specific data set was not considered appropriate. Instead, the focus was placed on ensuring the simulated plasma and hepatic concentrations both quantitatively remained within the experimentally measured ranges and matched qualitative features in the data such as periods of increase or decrease, and appropriately timed peaks and troughs when simulating a wide range of different feeding conditions and insulin sensitivities using a number of data sets (see S1 Text and S2 Text). The fitting was performed by hand, first ensuring the model produced realistic hepatic and plasma concentrations and rates of oxidation when provided with constant inputs (comparing with concentration and rate ranges measured in the literature and with the simulations from an existing model of glycogen storage [47]), before extensively refining the parameter values by comparison with more complex data sets. Where possible, time-series measurements for variation in the concentrations of several molecules or rates of several processes before, during and after feeding were used, including data for both metabolically healthy and insulin resistant patients. Due to inter-individual variability in metabolism, there is inevitably non-uniqueness in parameter values and the model can be considered to represent a single near-average individual. The sensitivity analysis performed in this report aims to understand the consequences of variability in these parameters.

To allow for the inclusion of zonated enzyme expression, the base values of the rate constant (which are zone independent) were altered in each compartment according to whether the enzymes in each process are known to be upregulated or downregulated in that region of the sinusoid (Fig 2). Since continuous changes in enzyme expression are seen for all of the processes included in the model, rather than the step-wise changes in, for example, cholesterol synthesis, the zonation of each process could be set based on the experimentally measured periportal to pericentral ratio of the rate limiting enzymes with a continuous change between compartments. The periportal to pericentral ratio used is stated for each process in Table 2. The representation of zonation in the model along with the experimental data used to set the zonation in rate constants across the sinusoid is discussed in detail in S1 Text–section 1.6.

#### Simulations and testing

Simulations were run using the XPPAUT ODE solver using a 4^th^ order Runge-Kutta method. The concentrations of the various plasma and cytoplasmic molecules are calculated in μM (μmoles/L). To allow easier comparison with experimental data, hepatic triglyceride concentrations were converted to a percentage of total cell mass for this report as discussed in S1 Text. The model file is provided in S1 Code.

Due to this spatially discretised representation of blood flow and the discrete time steps used in simulations, several tests were performed to ensure the simulated data are stable and do not show numerical diffusion. Firstly, simulations were run with varying time steps and compartment numbers to assess stability in the model outputs. The time step was increased from 0.01 seconds to 1 second, and the simulated data were stable and consistent for time steps below 0.4 seconds. When running simulations for the data in the report, a time step of 0.05 seconds was used. Similarly, when the number of compartments was varied from 3 to 48, no numerical diffusion effects were seen for low compartment numbers. For greater than 6 compartments, no qualitative changes in the simulated data for concentrations and rates across the sinusoid occurred due to under-sampling (such as the appearance of missing maxima or minima) for either metabolically healthy or insulin resistant individuals. Finally, oxygen, insulin and glucagon are degraded at constant rates across the sinusoid in the model such that a theoretical concentration curve can be calculated and compared with the values produced by the model. In each case, when using 8 compartments and a time step of 0.05 seconds, the average difference between simulated and theoretical value was less than 0.1% of the average value.

### Inputs, Insulin Resistance, NAFLD and Sensitivity Analysis

#### Inputs

To simulate an input sequence roughly equivalent to that of a daily feeding cycle excluding sleep, spiked glucose and FFA inputs with a period of 4 hours were used providing both periods of post-prandial glycogen and triglyceride synthesis and periods of their breakdown to glucose and FFAs between meals. The additional changes to hormone release and energy metabolism occurring during sleep or prolonged starvation in addition to exercise were considered beyond the scope of the model at present. Carbohydrates are broken down to sugars before entering the digestive system whilst lipids are initially broken down to FAs (and monoacylglycerides) before they can be absorbed by enterocytes in the intestine. Long chain FAs are generally converted back into triglycerides and enter the blood stream through the lymph system. Short to medium chain FAs (and some long chain FAs) enter the blood stream directly through the portal vein [63]. In the model, an FFA input to the body compartment is used to represent lipid input from digestion, with a triglyceride synthesis term representing both the reforming of triglycerides in the gut and triglyceride synthesis by tissues other than liver around the body.

$$v_{input}*\sin^{6}\;\left(\frac{pi}{2(hours)}\right)\;\text{-}>Spiked\;inputs\;with\;4\;hour\;period$$

When using this input cycle, simulations were run until equilibrium was reached between subsequent cycles. Average concentrations and rates over each 4 hour cycle are then presented. For a moderate diet, *v*_*input*_ was set such that the carbohydrate and lipid inputs over each cycle matched the average input per meal in a study performed by Daly *et al*. [64]. The inputs correspond to a mixed meal in which the majority of carbohydrates are in the form of fast release starches with roughly 78.1g of carbohydrate and 22.2g of lipid inputted per cycle as discussed in S1 Text.

Because a separate adipose compartment was not included, adipose lipid storage and adipocyte proliferation could not be included in the model. As a result, the short and medium term effects of high fat diets on plasma FFA and triglyceride concentrations are exaggerated because the fats are not removed from circulation. To compensate, inputs representing high fat and very high fat diets were set by matching the resulting simulated plasma FFA and triglyceride concentrations to those seen in overweight and obese individuals, rather than matching the increase in dietary intake. A 12.5% increase in FFA input was used to represent high fat intake, which results in simulated plasma triglyceride and glucose concentrations close to the average of those measured in overweight individuals by Sindelka *et al*. (25kg.m^-2^<BMI<30kg.m^-2^) [65]. A 25% increase was used to represent very high fat intake, which results in triglyceride and FFA concentration towards the high end of the values measured in obese individuals (BMI>30kg.m^-2^), consistent with a severe increase in dietary fat content [65, 66]. High and very high carbohydrate intake diets were simulated using equivalent percentage increases in glucose input.

#### Insulin resistance

IR was simulated by multiplying the detected insulin concentration by an IR constant with a value of less than one (K_IR_ < 1). Throughout the report, severe IR corresponds to a value of K_IR_ = 0.015 and developing IR to K_IR_ = 0.05. On a moderate diet, developing IR leads to hyperinsulinaemia but only a rise of 1.25mM in average glucose concentration as raised insulin levels compensate for decrease sensitivity. Severe IR meanwhile causes postprandial hyperglycaemia consistent with the development of T2DM.

## Results

### Representing NAFLD in the Model

In the following section, IR with and without increased SREBP-1c expression and varying dietary intake are simulated to assess to what extent these account for the experimentally observed changes in lipid levels, glucose regulation, ATP levels and metabolic rates in NAFLD [1, 2, 6–9].

#### Insulin resistance and SREBP-1c

**Fig 3:**
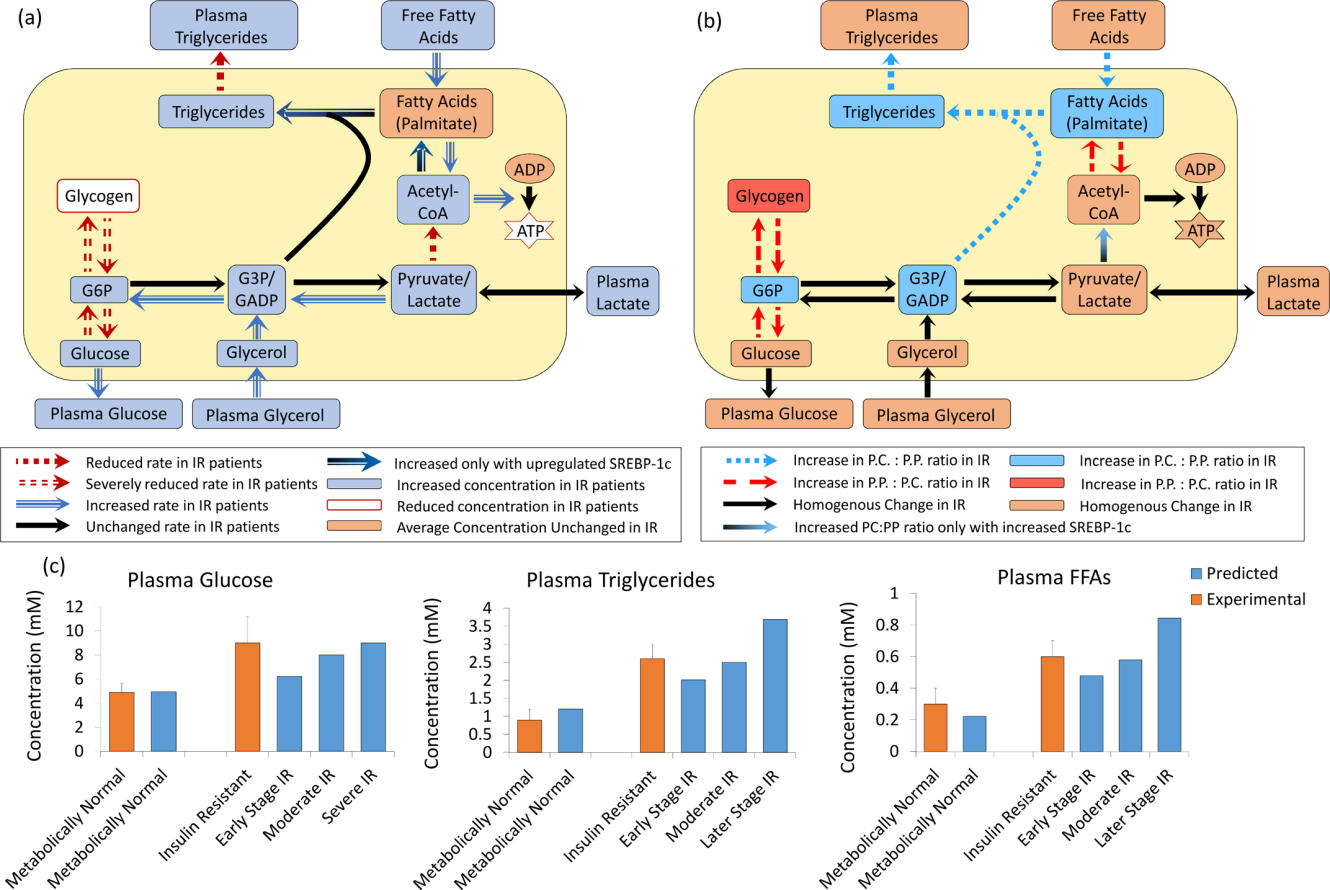
(a) The effects of simulating IR on total hepatic metabolism (averaged across the sinusoid). (b) The heterogeneity in the effects of simulating IR across the sinusoid. (c) The effects of simulating increasing severities of IR on plasma triglyceride, glucose and FFA concentrations compared with experimental data from Sindelka et al [65] and Burnt et al. [66].

**Fig 4:**
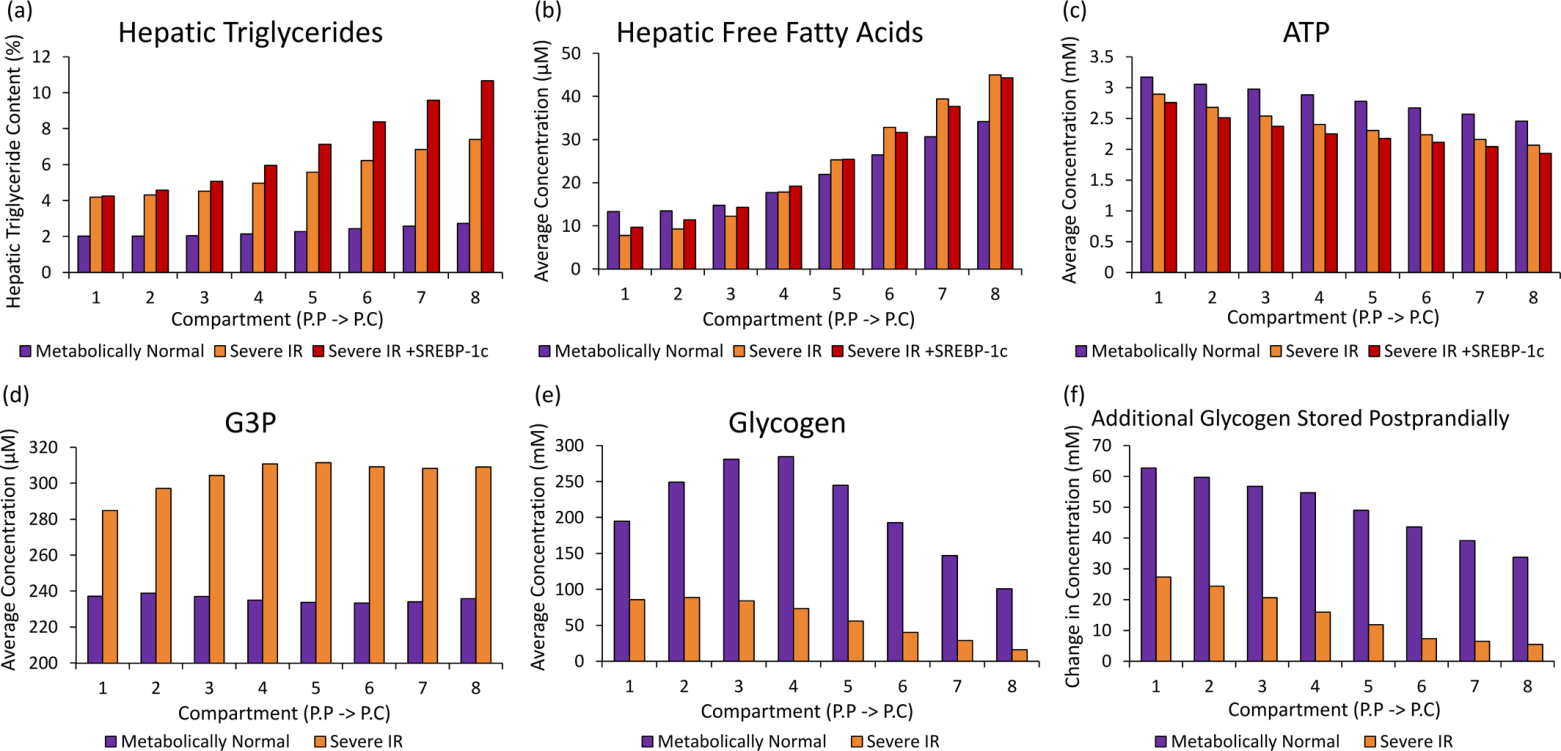
Metabolite concentrations when simulating NAFLD. (a) The average triglyceride (b) FFA, (c) ATP, (d) glycerol-3-phosphate and (e) glycogen concentration and (f) the difference between postprandial peak and pre-prandial trough glycogen concentrations in the different regions of the sinusoid when simulating (purple) a MH individual, (orange) IR alone and (red) IR with increase SREBP-1c expression using a moderate intake diet.

**Fig 5:**
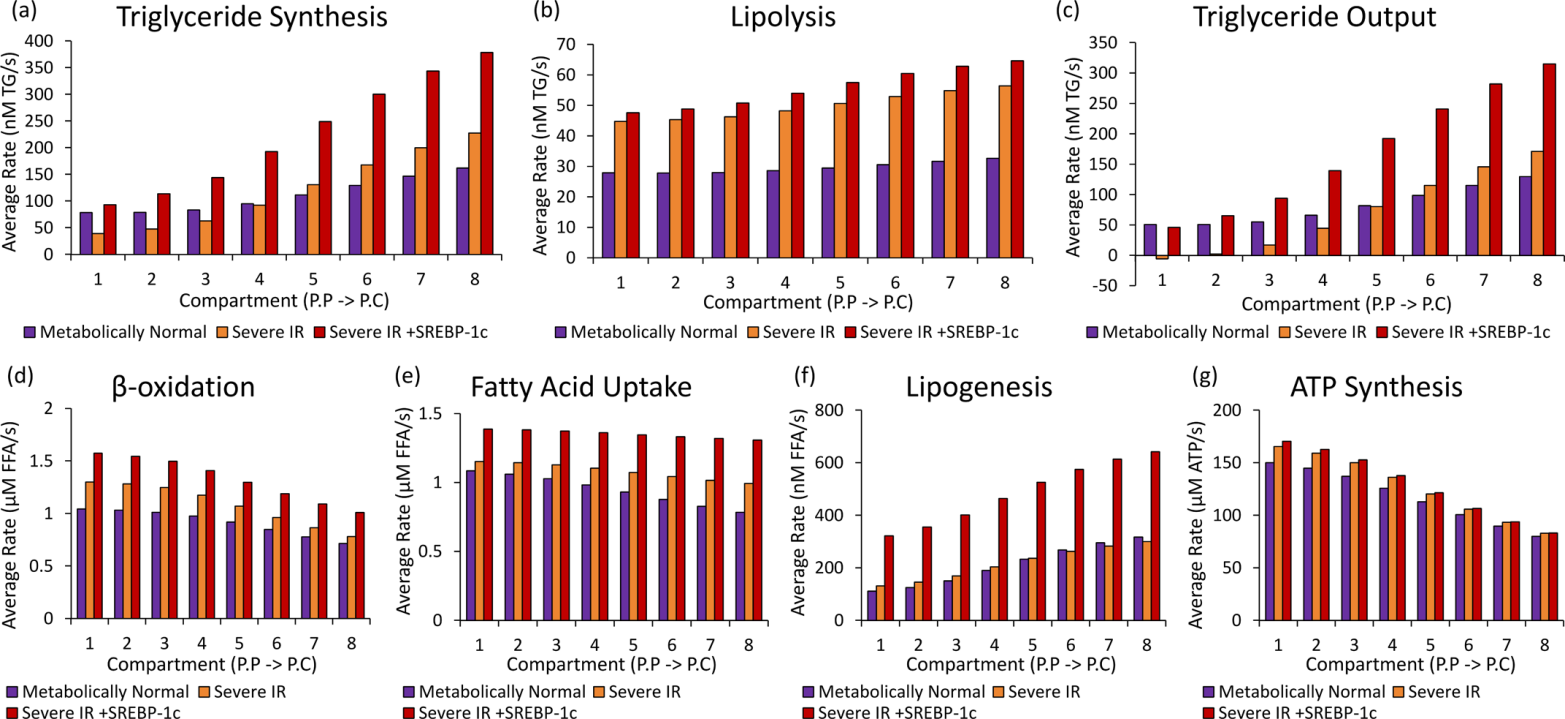
Metabolic rates when simulating NAFLD. The average rates of (a) triglyceride synthesis, (b) lipolysis, (c) triglyceride release as VLDL, (d) β-oxidation (e) FFA uptake, (f) lipogenesis and (g) ATP synthesis from acetyl-CoA in the different regions of the sinusoid when simulating (purple) a MH individual, (orange) IR alone and (red) IR with increase SREBP-1c expression using a moderate intake diet.

Figs 3–5 summarise the simulated effects of IR on liver metabolism and the simulated effects of increased expression of pro-lipogenic regulatory protein SREBP-1c in addition to IR. IR alone is simulated by reducing the detection by a factor *K*_*IR*_ < 1 such that cells effectively experience an insulin concentration lower than the real concentration. However, this fails to account for the increase in SREBP-1c expression seen in NAFLD patients *in vivo* [1, 2]. In metabolically healthy individuals, SREBP-1c is stimulated by insulin and, therefore, a fall in expression would be expected insulin resistant NAFLD patients [13, 67–69]. To account for the counter-intuitive increase in SREBP-1c seen experimentally, simulations were run for an insulin resistant individual with continuous stimulation (corresponding to a high 1nM insulin concentration) of lipogenesis and triglyceride synthesis.

Fig 3 summarises the simulated effects of IR with and without increased SREBP-1c expression on bulk hepatic metabolism (Fig 3A), on zonation in metabolism (Fig 3B) and on key plasma concentrations (Fig 3C). Figs 4 and 5 show the simulated effects of IR with and without increased SREBP-1c expression on concentrations of hepatic metabolites (Fig 4) and rates of key hepatic processes (Fig 5) across the sinusoid.

The majority of key metabolic changes known to occur in steatotic livers are seen in the model output data when simulating the direct effects of IR alone (without increased SREBP-1c expression). Firstly, pericentral-centered steatosis occurs when simulating system-wide IR (Fig 4A). For a severely insulin resistant individual on a moderate diet, the simulated average hepatic lipid content was more than doubled from 2.3% of total cell mass to 5.5%. The increase in concentration was largest in pericentral cells where the lipid content rose from 2.7% to 7.4%, consistent with the development of pericentral-centered steatosis. A 5% (50mg/g wet weight) liver triglyceride concentration is generally used as the criterion for diagnosing early-stage NAFLD [25].

Both experimentally and in the model simulations, insulin resistant livers are unable to store sufficient glycogen (Fig 4D), leading to post-prandial hyperglycaemia (Fig 3C). Consistent with experimental observations [70], pericentral cells show a more severe loss in their ability to store and release glucose throughout the meal cycle than periportal cells (Δ(compartment 1) = 27.4mM vs Δ(compartment 8) = 5.5mM (in units of glucose) (Fig 4F)).

However, in order for the model to fully recreate the metabolic changes seen *in vivo*, raised SREBP-1c expression must additionally be simulated. In particular, when simulating the direct effects of IR alone, the data differ from *in vivo* findings for NAFLD in the lipogenesis rates. *In vivo*, although the majority of hepatic lipids in fatty liver are known to originate from uptake, increases have also been measured in the rates of *de novo* lipogenesis and triglyceride synthesis [2, 36, 71, 72]. When simulating IR in the model, the average rates of these processes were roughly unchanged (Fig 5A and 5F) and hepatic steatosis arose as a result of increased lipid uptake alone (Fig 5C and 5E). However, when additionally simulating increased SREBP-1c expression, the rates of lipogenesis and triglyceride synthesis increased to 2.31 and 2.05 times the MH rate respectively (Fig 5A and 5F), consistent with *in vivo* NAFLD [2, 36, 71, 72]. Increased SREBP-1c expression led to more severe pericentral-centered steatosis, with the simulated compartment 8 (most pericentral) lipid content higher than 10% of total cell mass even, for the moderate intake cycle used (Fig 5A).

When measured experimentally, hepatic glucose oxidation is markedly reduced in NAFLD, with almost all energy produced through β-oxidation [73–79]. When simulating IR with increased SREBP-1c expression in the model, a 45% increase in β-oxidation (Fig 5D) and a 13.1% reduction in the average rate of acetyl-CoA production via glycolysis occurred, consistent with the experimental observations [73–80] (Fig 5D).

Despite the increase in FA oxidation, reduced ATP concentrations have been measured experimentally in NAFLD livers [74–77, 81–84]. Also, increased mitochondrial ROS production has been measured, suggesting overactive but dysfunctional ATP synthesis pathways [13, 83, 85–87]. These findings are likely to be, at least partially, due to a reduction in activity in electron transport chain (ETC) proteins (reviewed in [13]). When simulating IR with increased SREBP-1c expression in the model, a 19.5% fall in ATP concentration (Fig 4B) occurred despite a 9.3% increase in the rate of oxidation of acetyl-CoA in the citrate cycle (Fig 5G). This occurred even though the model does not include the reduction in ETC protein activity, and results from increased ATP consumption in lipogenesis, triglyceride synthesis and β-oxidation combined with reduced ATP production from glycolysis. As NAFLD develops *in vivo*, additional drops in ATP concentration would be seen due to reduced ETC protein activity [73–77].

Therefore, whilst pericentral-centered steatosis, reduced glycogenesis and key alterations to energy metabolism seen in NAFLD patients occur in the model when simulating IR alone, increased SREBP-1c expression is additionally required to fully replicate the metabolic changes occurring in NAFLD. In the following sections, both the effects of IR alone and IR combined with increased SREBP-1c expression are simulated.

#### Dietary intake and the development of steatosis

**Fig 6:**
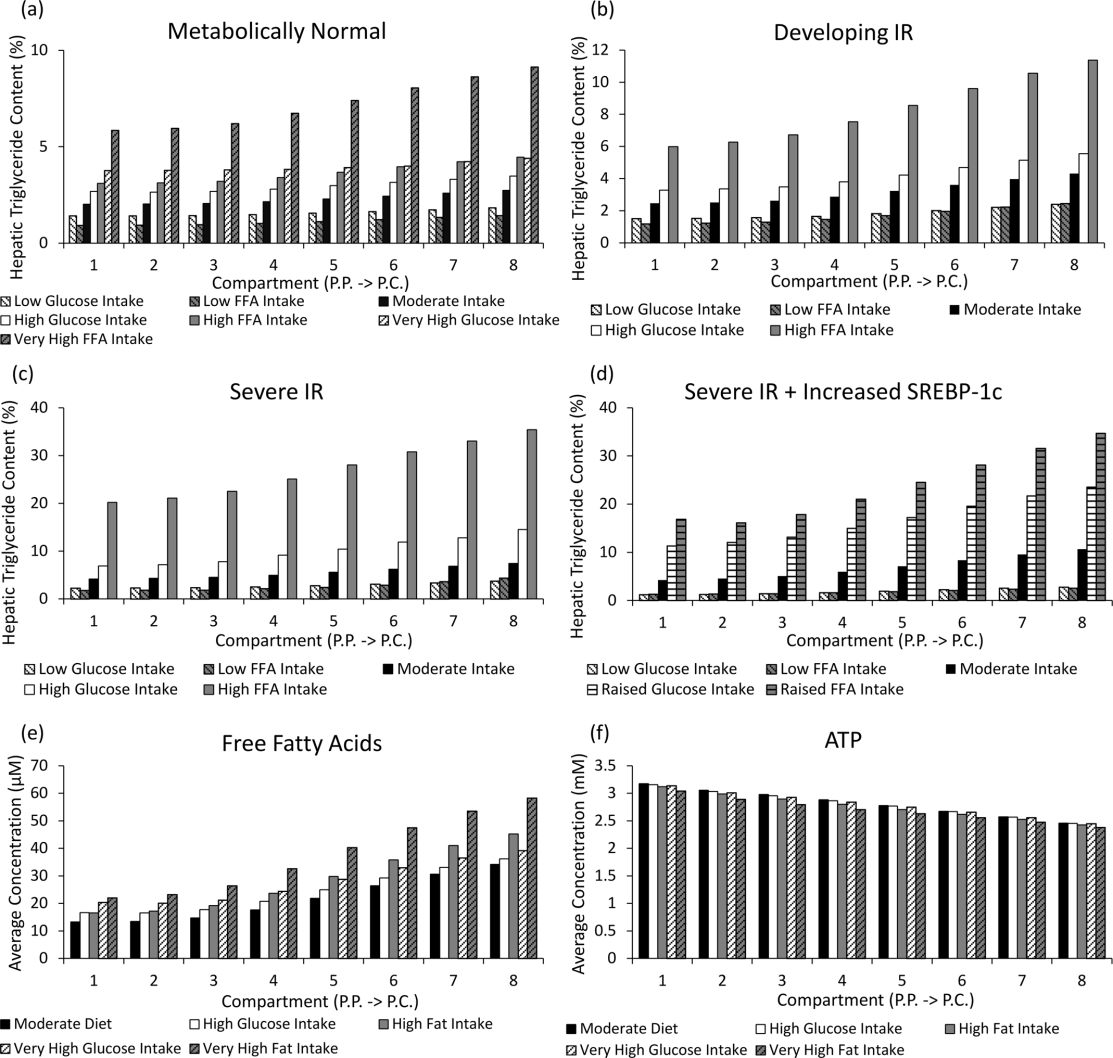
The simulated effects of varying dietary intake in the model. (a-d) The average hepatic lipid content across the sinusoid when simulating varying glucose and FFA intake diets in individuals with (a) MH insulin sensitivity (100%, K_IR_ = 1), (b) developing IR (5%, K_IR_ = 0.05), (c) severe IR (1.5%, K_IR_ = 0.015) and (d) severe IR in combination with increased SREBP-1c expression. (e-f) The effects of high fat and carbohydrate (glucose) intake on (e) ATP concentrations and (f) FFA concentrations across the sinusoid when simulating a MH, insulin sensitive individual. High and low intakes correspond to a sustained 12.5% difference in intake relative to the moderate intake det. Very high and raised intakes correspond to 25% and 5% increases respectively. The average concentrations over a 4 hours intake/output cycle are depicted.

Fig 6 shows the simulated effects of high fat intake and very high fat intake diets on hepatic triglyceride, FFA and ATP concentrations. When simulating insulin sensitive individuals, high intake and very high intake of fats caused relatively moderate increases in the average hepatic triglyceride content from 2.3% on a moderate diet to 3.6% and 7.2% respectively (Fig 6A). Therefore, hepatic lipid content is only higher than the 5% criteria at which early-stage NAFLD is diagnosed when simulating obese individuals with severe plasma hyperlipidaemia in the absence of IR. Hepatic FFA concentrations were increased from 21.5μM on a moderate diet to 28.6μM and 38.0μM when simulating high and very high fat diets respectively (Fig 6E). A 5% reduction in ATP concentration occurred when simulating a very high fat diet due to increased allosteric inhibition of glucose oxidation (Fig 6F). High and very high carbohydrate diets were also simulated. Smaller increases in the simulated hepatic triglyceride levels occurred than for increased lipid intake and even for very high intake, the hepatic triglyceride concentration remained less than 5% (Fig 6A).

When combined with IR, high dietary intake caused far more severe steatosis in the model. In the case of severe IR (K_IR_ = 0.015) (Fig 6B–6D), the average concentration increased from 5.5% when simulating a moderate intake diet to 10.0% and 27.0% when simulating high carbohydrate and high fat intake diets respectively (Fig 6C). Severe IR combined with increased SREBP-1c expression caused even more extreme increases in hepatic triglyceride concentrations due to additional hepatic lipogenesis (Fig 6D).

In reality, adipose uptake of plasma lipids and increased adipocyte proliferation are likely to prevent such rapid rises in hepatic steatosis concentrations. However, these severely raised values are consistent with the dangerous effects of high fat intake on metabolism in untreated insulin resistant individuals.

### The Causes of Pericentral Centered Steatosis in Insulin Resistant NAFLD Individuals

#### Triglyceride synthesis, output and lipolysis

The processes directly determining the triglyceride concentration are triglyceride synthesis (Fig 5A), lipolysis (Fig 5B) and the net rate of triglyceride output (Fig 5C) (release as VLDL minus the slow uptake from plasma). Lipolysis only occurs at a very slow rate in liver [88] and, although an increase in rate occurred when simulating IR, the process is not a major determinant of the hepatic triglyceride concentration. Triglyceride output and uptake, meanwhile, depend only on the plasma and hepatic triglyceride concentrations in the model, and cannot account for the variation in steatosis across the sinusoid. Instead, the zonal differences arise in the rate of synthesis.

Even when simulating MH individuals, the rate of triglyceride production was higher in the pericentral half of the sinusoid than in periportal cells. The simulated periportal to pericentral triglyceride synthesis ratio (pp:pc) was 1:1.64, consistent with the ratio of 1:1.58±0.34 seen experimentally (Fig 5A) [89]. This arises as a result of the upregulated lipogenic pathways in pericentral cells and upregulated β-oxidation in periportal cells. When simulating NAFLD (IR with increased SREBP-1c expression), the gradient in the rate of triglyceride synthesis across the sinusoid became steeper (pp:pc = 1:2.34), leading to greater steatosis in pericentral cells.

The enzymes mediating triglyceride synthesis have homogenous expression across the sinusoid in the model. The large heterogeneity in rate across the sinusoid instead results from differences in the concentrations of the two substrates of triglyceride synthesis: glycerol-3-phosphate (G3P) and FFAs.

#### FFA concentration: Lipogenesis, uptake and oxidation

When simulating MH individuals, the FFA concentration was higher in the pericentral compartments (pp:pc = 1:1.91) (Fig 4C), which is intuitive given that pericentral cells specialise in lipogenesis whilst periportal cells predominantly utilise fats in β-oxidation [90]. When simulating NAFLD, this gradient in FFA concentration becomes more pronounced (pp:pc = 1:2.54). The processes directly determining the hepatic FFA concentration in the model are *de novo* lipogenesis (Fig 5F), uptake and release (Fig 5E), and β-oxidation (Fig 5D) along with triglyceride synthesis (Fig 5A) and lipolysis (Fig 5B).

The majority of additional lipids in NAFLD arose from uptake in the simulations (ΔRate = 404nM FFA/s; Fig 5E), although *de novo* lipogenesis also increased as a result of SREBP-1c expression (ΔRate = 276nM FFA/s; Fig 5F). The largest increase in uptake occurred in pericentral cells where it increased by 471nM FFA/s compared with 337nM FFA/s in periportal cells. FA uptake proteins have higher expression in periportal cells and even when simulating IR, the total rate of uptake was still larger in periportal than pericentral cells. However, under conditions of insulin resistance, where passive uptake dominates rather than insulin-stimulated scavenging, the simulated gradient in the rate of FFA uptake across the sinusoid was less steep. The increase in *de novo* lipogenesis was also larger in pericentral cells, where it increased by 311nM FFA/s compared with 242nM FFA/s in periportal (Fig 5F). This is as a direct result of the pericentral zonation of lipogenesis enzymes.

The rate of β-oxidation is increased by 411nM FFA/s when simulating NAFLD providing a compensatory mechanism by which some of the extra fats are removed (Fig 5D). In the model this occurred due to increased substrate and reduced insulin inhibition, although *in vivo* it is thought that additional signalling pathways may further increase β-oxidation (reviewed in [13]). This increase was largest in oxygen rich periportal cells (ΔRate = 490nM FFA/s) where greater quantities of acetyl-CoA are required for the citrate cycle compared with pericentral cells (ΔRate = 332nM FFA/s) (Fig 5G).

Since the largest increase in uptake occurred in pericentral cells whilst the largest increase in lipid oxidation occurred in periportal cells, FFA availability for triglyceride synthesis increased in pericentral cells.

#### G3P concentration: Glycogen storage and carbohydrate metabolic intermediates

G3P, which is closely related to glyceraldehyde-3-phosphate (GADP), an intermediate of glycolysis, forms the carbon backbone in triglyceride synthesis. The G3P concentration was raised across the sinusoid when simulating insulin resistant individuals, and this increase was larger in the pericentral half of the sinusoid (75μM) than in the proximal periportal cells (48μM) (Fig 4E). Increased SREBP-1c expression affects triglyceride concentrations through changes in lipogenesis and triglyceride synthesis, and has little effect on the G3P concentration.

The increase in G3P concentration when simulating IR individuals occurred because hepatocytes, particularly towards the pericentral end of the sinusoid, are unable to properly clear glucose intermediates. When simulating IR, glycogen concentrations were severely reduced across the sinusoid (Fig 4D). However, as discussed above, pericentral cells showed a more severe reduction in glucose storage than periportal cells when simulating IR (Additional glucose stored postprandially: Δ(compartment 1) = 27.4mM, Δ(compartment 8) = 5.5mM) (Fig 4F).

In addition, pericentral cells consume less glucose in oxidative metabolism than periportal cells due to their low oxygen environment (8.1μM acetyl-CoA/s vs 13.0μM/s when simulating NAFLD; Fig 5G). These effects caused a build-up of carbohydrate metabolism intermediates, including G3P, particularly in pericentral cells.

This increase in G3P concentration, which remains lower than the K_M_ value for the glycerol backbone binding to glycerophosphate acyltransferase (460μM), means that FFAs are more rapidly converted to triglycerides, causing steatosis [91].

### The Effects of Metabolic Variation between Individuals on Steatosis Development in NAFLD

#### Key determinants of total hepatic triglyceride level

Sensitivity analysis was performed on the rate constants in the model to identify the hepatic processes most likely to account for differences in susceptibility to steatosis (Table 3). The rate constants determine the overall maximum activity of the enzymes mediating each process. Metabolic variations in numerous tissues around the body are likely to affect hepatic steatosis via plasma lipid concentrations. However, because the model presented here is primarily of liver metabolism, we focus specifically on variations in the expression and activities of enzymes mediating hepatic energy metabolism. Each rate constant was increased or reduced by 10% when simulating an otherwise metabolically normal individual. Variations of this size are large enough to have a notable effect on hepatic and plasma concentrations and allow us to identify the relative importance of processes, but remain in a range which would realistically be expected between individuals under non-pathological conditions. The model was provided with constant glucose and FFA inputs such that, when using the unaltered parameter values, the plasma glucose, triglyceride and FFA concentrations remained at 5.03mM, 1.05mM, and 432μM respectively. Simulations were run for 36 hours from these initial values after changing each rate constant.

**Table 3:** The effect of varying the baseline rate constants, v_b_, for the various hepatic metabolism processes included in the model on cellular and plasma FFA and triglyceride concentrations.

Process	Change in Cellular Fatty Acid (μM)		Change in Cellular Triglyceride (μM)		Change in Plasma FFA (μM)		Change in Plasma Triglyceride (μM)	
	Increased *v*_*b*_ (+10%)	Reduced *v*_*b*_ (-10%)	Increased *v*_*b*_ (+10%)	Reduced *v*_*b*_ (-10%)	Increased *v*_*b*_ (+10%)	Reduced *v*_*b*_ (+10%)	Increased *v*_*b*_ (-10%)	Reduced *v*_*b*_ (+10%)
β-oxidation	-2.7	5.4	-808.2	2782.5	-15.1	32.2	-36.6	125.7
ATP Synthesis	-1.9	2.1	-1271.9	1818.1	-12.2	13	-57.0	82.7
Glycolysis 1: (G6P to G3P) mediated by PFK	-0.6	0.6	562.6	-587.6	7.2	-7.7	17.9	-19.1
Gluconeogenesis 2 (G3P to G6P) mediated by FBP	0.0	-0.2	-517.1	599.3	-7.4	8.4	-18.5	21.1
Glycolysis 2: (G3P to Pyr) mediated by PK	2.3	-2.4	572.0	-588.9	12.0	-12.2	26.5	-27.2
Gluconeogenesis 2 (Pyr to G3P) mediated by PEPCK	-1.5	1.8	-464.9	550.4	-8.7	10.4	-21.0	24.8
Pyruvate Dehydrogenase	1.6	-1.6	526.4	-549.9	9.4	-9.7	23.2	-24.3
VLDL Synthesis and Release	0.3	-0.3	-362.9	373.6	7.0	-7.0	23.0	-23.4
Triglyceride Synthesis	-1.5	1.8	237.5	-255.9	1.0	-1	4.5	-4.6
FFA Uptake	3.1	-3.0	260.7	-143.0	-28.5	36.4	-53.9	64.2
Lipogenesis	0.5	-0.5	183.0	-183.4	3.1	-3.2	7.8	-9.0
Glucokinase	1.6	-1.3	-90.4	262.8	79	-68.2	-56	65.9
Triglyceride Cross-Membrane Transport	-0.1	0.1	102.5	-114.8	-1.9	2.2	-6.4	7.2
G6Pase	-0.7	1.0	100.6	-45.5	-35	43.0	30	-31.2
Lipolysis	0.3	-0.4	-69.0	70.3	-0.5	0.4	-1.7	1.7
Glycogen Synthase	0.4	-0.4	-39.4	62.8	13	-14.0	-11	13.0
Glucose Uptake	0.5	-0.4	-24.4	63.8	21	-22.7	-15.5	19.8
Glycogen Phosphorylase	-0.2	0.2	16.2	-10.1	-5.6	5.9	4.4	-4.4

Hepatic and plasma triglyceride concentrations were most sensitive to the rate constants for β-oxidation and for ATP synthesis through the citrate cycle (Table 3), particularly when these processes were suppressed. Reducing the rate constants for β-oxidation and ATP synthesis caused increases in the simulated hepatic triglyceride concentration of 2.78mM and 1.82mM over the 36 hours respectively. Increasing the rate constants, meanwhile, caused reductions of 0.81mM and 1.27mM respectively. These large changes suggest that metabolic variations affecting the rate of oxidation of fats are likely to be a major determinant of inter-individual susceptibility to the development of steatosis. Consistent with this, hepatic triglyceride concentrations were also sensitive to changes in the rate constants mediating glycolysis, gluconeogenesis and acetyl-CoA synthesis. All of these processes alter the production of acetyl-CoA from glucose, and hence, the allosteric inhibition of β-oxidation. A 10% variation in the any of the five rate constants mediating these processes caused a 0.45–0.6mM variation in simulated hepatic triglyceride content after 36 hours (Table 3).

In addition to the oxidation of fatty acids, hepatic triglyceride levels also showed moderate sensitivity to the rate constant for triglyceride release as VLDL. A 10% variation in rate constant caused a 0.36–0.37mM change in simulated hepatic triglyceride levels after 36 hours (Table 3).

Hepatic lipid concentrations showed only a relatively weak sensitivity to changes in the rate constant for lipogenesis. Although *de novo* lipogenesis contributes less to hepatic lipid levels than FFA uptake, its contribution is not insignificant and changes in the rate of lipogenesis would be expected to impact upon hepatic triglyceride levels. However, lipogenesis is strongly allosterically regulated by the concentration of its product and alterations in maximum enzyme activity (rate constant) are partially compensated by changes in allosteric inhibition. Similarly, the rate constant for triglyceride synthesis only had a moderate effect on hepatic and plasma lipid levels. Due to the low concentration of cellular FFAs, the rate of triglyceride synthesis is predominantly determined by the availability of FFAs, rather than the enzyme activity.

Finally, hepatic triglyceride levels also showed a comparatively weak sensitivity to variations in the rate constant for FFA uptake. However, changes in FFA uptake did have a larger effect on plasma lipid levels.

#### Key determinants of sinusoidal steatosis location

10% variations in the rate constants of the processes in the model had little effect on the predominant location of steatosis across the sinusoid. Instead, to determine the hepatic metabolic variations most likely to account for differences in the location of steatosis, sensitivity analysis was performed on the zonation constants in the model (Table 4). These constants determine the difference between periportal and pericentral activity of enzymes mediating each process. Positive values correspond to periportal zonation whilst negative values correspond to pericentral zonation. The constants were increased or reduced by 0.2 corresponding to a 20% increase in periportal and 20% reduction in pericentral cells or *vice versa*, which is well within the range of variation expected non-pathologically (S1 Text–Table 2). The effects of these increases and reductions on the pericentral to periportal triglyceride ratio are presented when simulating a severely insulin resistant individual (K_IR_ = 0.015) with increased SREBP-1c expression on a moderate intake diet.

**Table 4:** The effect of altering the zonation constants k_n_ of lipid metabolism processes on steatosis location (compartment1: compartment 8 triglyceride ratio) when simulating for IR patients on a moderate diet.

Process Altered	Periportal: Pericentral Triglyceride Ratio		Difference in the change in triglyceride concentration between pericentral and periportal cells when varying k_n_
	Unchanged Metabolism: 1:1.80		
	20% More Periportal (Δk_n_ = + 0.2)	20% More Pericentral (Δk_n_ = - 0.2)	${\frac{\left(\Delta {TG}_{pc}-\Delta {TG}_{pp}\right)}{{TG}_{av}}}_{\Delta kn=-0.2\to +0.2}^{†}$
Fatty Acid Uptake	1:1.27	1:2.62	0.91
Triglyceride Release as VLDL	1:2.22	1:1.47	-0.43
Acetyl-CoA Synthesis	1:1.64	1:1.98	0.22
β-oxidation	1:2.01	1:1.64	`-0.22
Glycolysis 1 (G6P-> GADP)	1:1.73	1:1.86	0.08
Lipogenesis	1:1.75	1:1.85	0.08
Gluconeogenesis (GADP -> G6P)	1:1.88	1:1.74	-0.07
Gluconeogenesis 1 (Pyr -> GADP)	1:1.80	1:1.80	0.06
Glycolysis 2 (GADP -> Pyr)	1:1.79	1:1.80	-0.06
Triglyceride Synthesis	1:1.75	1:1.85	0.05
Triglyceride Uptake	1:1.75	1:1.89	0.04
Glucokinase	1:1.80	1:1.80	0.02
G6Pase	1:1.81	1:1.80	-0.00
Lipolysis	1:1.82	1:1.79	-0.00

^†^ the change in compartment 8 triglyceride concentration minus the change in compartment 1 concentration (normalised against the average triglyceride concentration) when simulating a 0.2 decrease in the zonation constant compared with a 0.2 increase to provide a measure of the sensitivity of triglyceride distribution to each rate constant.

While total hepatic triglyceride levels were most sensitive to changes in the rate of lipid oxidation, the location of steatosis is most strongly sensitive to changes in the zonation constants mediating FFA uptake and triglyceride output (Table 4). Variations in the zonation constant of FFA uptake had the largest effect, causing the periportal and pericentral triglyceride concentrations to change from 1:1.80 to 1:1.27 when increased (20% more periportal expression) and 1:2.62 when reduced (20% more pericentral expression). Increasing the zonation constant for triglyceride release as VLDL caused more strongly pericentral steatosis with a ratio of 1:2.22, whilst reducing the constant gave a ratio of 1:1.47. This suggests that the inter-individual variation in the location of steatosis seen *in vivo* is likely to be at least partially accounted for by differences in the zonation of lipid uptake and output proteins.

The zonation constants for β-oxidation and acetyl-CoA synthesis (mediated by the pyruvate dehydrogenase complex) from pyruvate also had a notable effect on the location of steatosis (Table 4). Alterations to the zonation constant for β-oxidation directly affect the rate at which fats are oxidised and removed from the cells in each region of the sinusoid. Similarly, alterations to the zonation constant for acetyl-CoA synthesis from pyruvate alter the relative amount of acetyl-CoA produced from glucose in each region and, as a result, the allosteric inhibition of β-oxidation.

Consistent with the results when altering the rate constants, variations in the zonation constants for *de novo* lipogenesis and triglyceride synthesis have little effect on steatosis location in the model. This is despite the continuous stimulation of lipogenesis and triglyceride synthesis through increased SREBP-1c expression. Therefore, non-pathological inter-individual variations in the expression of lipogenesis enzymes are not predicted to account for differences in either susceptibility to steatosis or the distribution of fats across the sinusoid seen *in vivo*.

## Discussion

### A Computational Model of Hepatic Metabolism across the Sinusoid Allows Study of the Heterogeneous Effects of Metabolic Dysregulation

A computational model of hepatic glucose and lipid metabolism across the sinusoid was presented and used to study the heterogeneity in metabolic dysfunction across the sinusoid in NAFLD. The substrate, allosteric and hormonal dependences of each process were based upon existing literature. Zonation in the model was based on measured differences in the activities of key enzymes published in the literature and validated against data for the rates of key processes in different regions of the sinusoid.

Using a computational model of this form allows us to simulate for the changes in the rates of metabolic processes and concentrations of intermediates in specific regions of the sinusoid under different feeding conditions and disease states which would not be feasible experimentally. Here the model has been used to understand the initial metabolic changes occurring in NAFLD, and the causes of heterogeneity in lipid build-up across the sinusoid. These predictions will allow for more focussed future experimental study, minimising the amount of complex, time-consuming and expensive experimentation required to understand the zone specific changes in disease.

### Insulin Resistance and Increased SREBP-1c Expression Are Required to Represent NAFLD in the Model

When simulating increased lipid intake in a metabolically healthy, insulin sensitive individual, the hepatic triglyceride content was raised but this increase was fairly moderate. Unless a very high fat diet (resulting in plasma lipid concentrations at the high end of those seen in obese individuals) was simulated, the hepatic lipid concentration remained lower than the 5% cut off at which NAFLD is diagnosed. Similarly, high glucose intake primarily caused increased glycogenesis rather than steatosis in insulin sensitive individuals. For more serious steatosis to develop in the model, IR was required. Early stage steatosis arose even when simulating a healthy (moderate intake) diet in an insulin resistant individual. Severe build-up of lipids in the liver occurred when high lipid intake, or to a lesser extent high glucose intake, was simulated in addition to IR. Across the range of insulin sensitivities, low FFA or glucose intake returned simulated plasma triglyceride levels to a healthy range.

Therefore, loss of insulin sensitivity in addition to excessive calorie intake is predicted to be required for more than early stage steatosis to arise. Given that fats in liver are known to cause both hepatic and peripheral desensitivity to insulin, sustained excessive calorie intake will strongly increase the chance of developing hepatic steatosis over time. However, these data highlight the effectiveness of a low fat diet as a treatment for NAFLD, even in insulin resistant patients.

In addition to the direct effects of IR, increased expression of SREBP-1c was required for the model to fully reproduce the metabolic changes seen in the early stages of NAFLD *in vivo*. In particular, the inclusion of increased SREBP-1c expression was required to replicate the increases in lipogenic rates seen *in vivo*. Additionally, both IR and increase SREBP-1c expression contributed to mitochondrial dysfunction, increased β-oxidation and reduced ATP levels in the simulations, consistent with *in vivo* observation.

SREBP-1c is a transcription factor which upregulates lipogenesis and triglyceride synthesis. Under normal conditions, its expression is stimulated by insulin. However, increased expression has been shown to occur in insulin resistant NAFLD patients. It is thought that insulin retains the ability to directly stimulate SREBP-1c expression, despite the loss of insulin sensitivity [13, 92]. Alternatively, it is possible that SREBP-1c is instead stimulated by hyperglycaemia or by the fats themselves [13, 92], similar to the effects of carbohydrate responsive element binding protein (ChREBP) [32]. These results highlight the importance of the resulting increase in lipogenesis and triglyceride synthesis in the pathology of the disease. However, the presence of steatosis was seen when simulating the direct effects of IR alone.

### Pericentral Centered Steatosis in Insulin Resistant Livers Results from Increases in FFA and G3P Concentrations in Pericentral Cells

One aim of this study was to understand the metabolic changes leading to the development of steatosis in NAFLD, with a particular focus on understanding the increased susceptibility of pericentral cells to lipid build-up and the resulting damage. Changes in the rate of triglyceride synthesis, rather than output or lipolysis accounted for the pericentral zonation in steatosis in model simulations. A reduction in net triglyceride output also contributed to overall increased triglyceride levels but did not show zone specific differences. The enzymes involved in triglyceride synthesis are not zonated in the model. The pericentral increase in triglyceride synthesis instead arose due to increases in the concentrations of G3P and FFAs.

Defective postprandial glycogen storage results when simulating IR and caused build-up of glucose metabolism intermediates, including G3P across the sinusoid. Pericentral cells show lower glycogen synthase activity than periportal cells [60]. When simulating insulin sensitive individuals, this was compensated by increased insulin receptor [93] and reduced glucagon receptor expression [94] in pericentral cells. However, when simulating IR, the zonation in hormone receptors no longer affects glycogen synthesis, and glycogen depletion is most severe in pericentral cells. Additionally, pericentral cells have fewer mitochondria and downregulated oxidative phosphorylation due to their low oxygen environment [60]. As a result, pericentral cells are able to metabolise glucose intermediates less rapidly.

Since glucose oxidation is suppressed in insulin resistant NAFLD patients and β-oxidation is upregulated, the periportal zonation of oxidative phosphorylation enzymes, along with those mediating β-oxidation, had an even larger effect on the simulated rate of oxidation of hepatic FFAs. Excess FFAs were metabolised more rapidly in periportal cells than pericentral.

Increased availability of hepatic FFAs arose from both uptake and *de novo* lipogenesis. The enzymes involved in glycolysis and lipogenesis show pericentral zonation and the increase in *de novo* lipogenesis was largest in pericentral cells. The enzymes mediating FFA uptake, meanwhile, show periportal zonation. When simulating MH individuals, FFA uptake is dominated by insulin stimulated scavenging leading to strongly periportal uptake. However, when simulating insulin resistant individuals, FFA uptake occurs due to the high plasma FFA concentration rather than insulin stimulation, and passive uptake dominates. Under these conditions, the periportal zonation of FA uptake proteins had a smaller effect on the rate of uptake. Therefore, although total uptake was still higher in periportal cells, a larger increase in rate occurred in pericentral cells.

Together these results suggest that the major differences between pericentral cells and periportal cells accounting for increased pericentral susceptibility to steatosis are lower expression of oxidative phosphorylation enzymes, β-oxidation enzymes and glycogen synthase along with higher expression of lipogenic enzymes in pericentral cells. These differences across the sinusoid account for a larger increase in FFA and G3P concentrations in pericentral cells in NAFLD and, therefore, result in higher triglyceride synthesis in these cells. Future experimental validation of these simulated data could be performed through the addition of radiolabelled substrates to measure the rates of conversion within individual regions of the sinusoid. As discussed S2 Text (section 1.1.5), data of this form has previously been published for metabolically healthy individuals, and the model outputs are consistent with experimental data in this case. Similar studies comparing metabolically healthy and non-alcoholic fatty liver disease patients are required. Studies destroying specific regions of the sinusoids and measuring the remaining activity for various processes could also be used to provide insight [95].

### Inter-individual Variation in Fatty Acid Oxidation Rates Most Strongly Affect Susceptibility to Steatosis

Sensitivity analysis was performed on the rate constants in the model to determine the processes most likely to account for variation in susceptibility to steatosis seen between individuals *in vivo*. Sensitivity analysis on the zonation constants for each process was also used to predict the key processes accounting for differences in the predominant location of steatosis seen *in vivo*.

The model simulations suggest that any inter-individual variations in the rate of metabolism of hepatic fats will have a large effect on susceptibility to NAFLD development. Variations in the rate constants for β-oxidation and acetyl-CoA consumption in the citrate cycle had notably larger effects on hepatic lipid levels than equivalent variations in the rate constants of other processes in the model. Furthermore, total hepatic lipid levels showed the next highest sensitivity to the rate constants for glucose uptake, glycolysis and acetyl-CoA synthesis from pyruvate all of which play a role in the allosteric suppression of β-oxidation by acetyl-CoA. Therefore, susceptibility to steatosis is predicted to be most strongly determined by the rate at which hepatocytes can metabolise fats.

As validation of this prediction, there is considerable evidence to suggest mitochondrial function, aerobic capacity and capacity for β-oxidation inversely correlate with liver fat percentage and prevalence of NAFLD [67, 96–104]. Furthermore, consistent with the high sensitivity of fat storage to oxidation rates, global knockout of ACC2 (resulting in reduced allosteric inhibition of β-oxidation) has been shown to reduce T2DM risk, obesity and adipose fat storage [105–107]. Liver specific knockout of ACC1 and ACC2 has been shown to reduce hepatic steatosis, although this resulted from both increased β-oxidation and reduced lipogenesis [108]. However, additional directed studies are required to determine whether higher β-oxidation and oxidative phosphorylation capacities protect against hepatic steatosis independent of confounding factors such as exercise or caloric intake.

Whilst the predominant location of steatosis showed some dependence to the zonation of enzymes mediating β-oxidation, it was far more sensitive to changes in the zonation constants for FA uptake and triglyceride release as VLDL. Therefore, inter-individual variation in the distribution of steatosis is predicted to be accounted for by differences in the zonation of proteins mediating lipid uptake and triglyceride release. At present, little experimental data exists to validate this prediction due to the difficulty involved in measuring the distributed activities of large numbers of enzymes. However, the model simulations allow for targeted potential future experiments, comparing the zonation in the activities of the proteins mediating lipid uptake and triglyceride release with the distribution of steatosis across a range of samples.

### Conclusions

Due to the large heterogeneity in metabolism across the sinusoid, a clear description of the metabolic changes occurring in each zone is required to fully understand NAFLD development and to optimise potential pharmacological interventions. In this study a computational model of sinusoidal metabolism was presented and used to simulate the development of NAFLD, focussing on the metabolic changes in individual zones. Consistent with experimental observation, both IR and increased SREBP-1c expression were required for the model to fully replicate the metabolic changes seen in NAFLD *in vivo*.

Simulations were run to identify the key differences between periportal and pericentral cells which account for higher pericentral susceptibility to steatosis. The majority of additional FFAs in NAFLD arise from fatty acid uptake rather than *de novo* lipogenesis both in the simulated data and experimentally [30, 34–36]. Although fatty acid uptake enzymes show periportal zonation, the switch from predominantly insulin stimulated fatty acid scavenging to passive diffusion reduced the effect of this heterogeneity on the rate of uptake across the sinusoid in the model simulations. Instead, the model simulations highlight the periportal zonation of oxidative phosphorylation and β-oxidation enzymes, along with the pericentral expression of lipogenesis enzymes as the key differences leading to a raised FFA concentration in pericentral cells when simulating insulin resistant NAFLD patients. Additionally, reduced insulin stimulation of glycogenesis caused the build-up of glucose intermediates, including G3P across the sinusoid. A more severe increase in pericentral and intermediate cells occurred due to the periportal zonation of glycogen synthase and of oxidative phosphorylation.

Sensitivity analysis was performed on the rate and zonation constants in the model to determine likely inter-individual differences in enzyme activities accounting for variation in susceptibility to NAFLD and steatosis distribution seen *in vivo*. Hepatic triglyceride levels are predicted to be most sensitive to inter-individual variations in the rate of FFA oxidation, either through differences in the overall rate of oxidation of acetyl-CoA or differences in the relative contribution of FFAs and glucose to oxidation. The predominant location of steatosis across the sinusoid meanwhile was most sensitive to changes in the zonation of proteins mediating FFA uptake or VLDL synthesis and release.

## Supporting Information

S1 Text Model Development. (DOCX)

S2 Text Comparisons with Experimental Data. (DOCX)

S1 Table Data for graphs. (XLSX)

S1 Code XPPAUT Model File. (ODE)
