# Supplementary material for: A Computational Model of Hepatic Energy Metabolism: Understanding Zonated Damage and Steatosis in NAFLD
Source: PLoS Comput Biol. 2016 Sep 15;12(9):e1005105. doi: 10.1371/journal.pcbi.1005105 (PMC5025084; doi:10.1371/journal.pcbi.1005105)
Supplement: S1 Text — (DOCX) [file pcbi.1005105.s001.docx]

# S1 Text. Model Development

## Model Structure, Blood Flow

Since blood is compartmentalized with average concentrations of variables in each compartment, rather than represented as a continuous change in plasma concentrations, a simplified representation of blood flow was used. In each time step, a proportion of the blood in each compartment moves to the subsequent compartment and is replaced by blood from the previous compartment. The plasma concentration of each metabolite or hormone (M) in each compartment changes according to the equations below. In the notation below, compartments $1\to n$ correspond to the proximal periportal to distal pericentral sinusoidal compartments while compartment $0$ is the body compartment. $n$ is the number of compartments used ( = 8 in this report). The constant $s$ is included since the body compartment is much larger than each liver compartment.

The blood flow and relative size of the hepatic and body compartments were set such that blood takes around a minute to make a circuit of the body and so that the liver blood volume is equal to roughly 0.8L with a total blood volume of 5L [[1-4](#_ENREF_1)].

$$\frac{dM_{i=1:n}}{dt}=bf*M_{i-1}-{bf*M}_{i}=bf*(M_{i-1}-M_{i})$$

$$\frac{dM_{0}}{dt}=\frac{bf*\left( M_{n}{-M}_{0} \right)}{s}$$

Rate of blood flow: $bf=0.15*n$ s^-1^ -> ($bf=$1.2s^-1^ for the 8 compartments used in this report.)

Rest of body to hepatic compartment ratio: $s=5*n$ -> ($s=$40 for the 8 compartments used in this report.)

## Representation of Processes in the Model

The concentrations of the various plasma and hepatic metabolites in the model are calculated in units of µM (µMoles/L). The concentrations of hormones are calculated in pM (pMoles/L).

The concentration of triglycerides, roughly equal to the total lipid concentration, was converted to a percentage of total cell mass before being presented to allow easier comparison with experimental data. This was calculated firstly assuming that in a cell containing no fat, the combined protein and cytoplasm concentration is 1000g/L. Secondly, that the protein and cytoplasm content of each cell remain constant independent of the lipid content, such that the cell volume (and total liver volume) expands as the lipid content increases, rather than the protein or cytoplasm content falling. In this case, the combined cytoplasm and triglyceride concentration becomes 1000g/v where v is the increased volume after including the lipid. An average molecular mass of 0.807 was used for triglyceride (equal to that of tripalmitin) $\frac{TG(g/v)}{TG\left( g/v \right)+Cyto\left( g/v \right)+Protein(g/v)}$=$\frac{\left( 100\% \right)*0.81*TG(g/v)}{0.81*TG\left( g/v \right)+1000}$.

Conversions are represented by Hill function dependences on the substrates and allosteric activators and inhibitors with a hormone dependent rate constant.

$$v=\frac{V_{max}{[S]}^{n_{Hill}}}{{K_{M}}^{n_{Hill}}+{[S]}^{n_{Hill}}}$$

Where $K_{M}$(mM) determines the substrate concentration at which enzymes are saturated, $V_{max}$(s^-1^) is the maximum rate and $n_{Hill}$ quantifies deviation from Michaelis-Menten behaviour.

Many of the equations correspond to processes with a number of intermediate enzymes rather than a single enzyme. However, Hill functions are used to represent the substrate dependence of the processes with K_M­_ values (and n_Hill_ where applicable) based on the literature for the rate limiting enzyme in the process.

Multiple substrates are represented in the model by multiplying the hill function substrate dependences. E.g. for three substrate S1, S2 and S3.

$$v=\frac{V_{max}{[S2]}^{n_{S1}}}{{K_{M}^{S1}}^{n_{S1}}+{[S1]}^{n_{S1}}}*\frac{{[S2]}^{n_{S2}}}{{K_{M}^{S2}}^{n_{S2}}+{[S2]}^{n_{S2}}}*\frac{{[S3]}^{n_{S3}}}{{K_{M}^{S3}}^{n_{S3}}+{[S2]}^{n_{S3}}}$$

Allosteric inhibition and activation are represented using a similar dependence on the inhibiting or activating molecule with inhibition or activation constants taken from the literature. E.g. for an inhibitory molecule, $i$, with concentration $[i]$:

$$v=\frac{V_{max}{[S]}^{n_{Hill}}}{{K_{M}}^{n_{Hill}}+{[S]}^{n_{Hill}}}\left( 1-\propto_{inh}\frac{{[i]}^{n_{inh}}}{{K_{i}}^{n_{inh}}+{[i]}^{n_{inh}}} \right)$$

Where $\propto_{inh}\leq1$ determines the maximum inhibition by $i$, ${K_{i}}^{n_{inh}}$ determines the concentration of $i$ at which inhibition is at half maximum, and $n_{inh}$ acts as the equivalent of the hill function.

In addition to the enzymatic conversions, the transport of molecules across the cell is represented in the model. A conversion factor $ctob=4$ is used to account for the difference in sinusoidal plasma and sinusoid volume. For uni-directional active uptake, Hill functions are used, treating the plasma molecule as the substrate and the cytoplasmic molecule as the product. For bidirectional facilitated diffusion, a Hill-type equation dependent on the difference in concentration between the cytoplasm and plasma is used. It is assumed that molecules from both inside and outside the cell contribute to the saturation of the reaction (a cross membrane transport protein is blocked by molecules moving both into and out of the cell such that the reaction is represented by an equation of the form:

$$v_{plasma\to cyto}=\frac{V_{max}\left( \left[ S_{plasma} \right]-\left[ S_{cyto} \right] \right)}{K_{M}+\left[ S_{plasma} \right]+\left[ S_{cyto} \right]}$$

Although these are heuristic representations of cross-membrane transport based on the stated assumptions, previous experimental studies have used hill-function equations with effective Michaelis-Menten constants to represent uptake [[5-7](#_ENREF_5)], and these forms have been used in previous models of liver metabolism [[8](#_ENREF_8)].

Hormonal regulation is represented in slightly different ways depending on how strongly and rapidly hormones are known to act on the process. In each case, hormonal regulation is based on the plasma concentration rather than modelling the receptors and downstream signalling in detail, as has been performed in previous models (e.g. [[9](#_ENREF_9)]). The hormonal regulation of each process is discussed in the following sections.

## Hepatic Metabolism

### Summary

#### Glucose Metabolism

The liver is the major organ responsible for the control of blood glucose concentrations. When blood glucose levels are high, glucose enters hepatocytes through the transmembrane carrier protein glucose transporter 2 (GLUT2) before being converted to glycogen via glucose-6-phosphate (G6P) under the influence of insulin (glycogenesis). When blood glucose levels are low, glucagon stimulates the release of glucose from glycogen stores (glycogenolysis). Insulin also stimulates the breakdown of glucose to two pyruvate molecules through glycolysis. This pyruvate can either be released into the blood as lactate or converted to acetyl-CoA for use in oxidative phosphorylation or in lipid metabolism. Glucagon, meanwhile, stimulates the reverse reaction converting pyruvate and lactate to glucose through gluconeogenesis. Glyceraldehyde-3-phosphate (GADP), an intermediate of glycolysis and gluconeogenesis, can be rapidly converted to glycerol-3-phosphate (G3P) which forms the glycerol backbone in triglyceride synthesis. Additionally, glycerol released in lipolysis is converted to G3P and GADP via the enzyme glycerol kinase. The majority of lipolysis occurs outside of the liver in adipose tissue. However, very little glycerol kinase activity is seen in adipose tissue and instead glycerol released in adipose tissue is recycled in the liver [[10](#_ENREF_10)].

#### Lipid and Energy Metabolism

Liver plays an important role in lipid metabolism and there exists a strong link between lipid metabolism and liver disease. Hepatic steatosis is seen in a range of liver conditions most commonly linked with alcohol, viruses such as hepatitis c or metabolic dysregulation and is known to play a role in the development of IR.

Pyruvate is converted to acetyl-CoA by a complex of three enzymes known as the pyruvate dehydrogenase complex (PDHC). These acetyl-CoA molecules can either enter the citrate cycle to provide fuel for oxidative phosphorylation of ADP to ATP or can be joined to form FA chains through *de novo* lipogenesis. Numerous FAs are produced *in vivo* of varying chain lengths. However, to reduce the computation required, the model assumes all FAs to be palmitate. Palmitate is the most common FA in animals, plants and microorganisms and corresponds to a chain of 8 acetyl-CoA molecules (16 carbons). Both the production of acetyl-CoA and lipogenesis are stimulated by insulin whilst the breakdown of palmitate back to 8 acetyl-CoA molecules (β-oxidation) is stimulated by glucagon.

When blood glucose levels peak, it is beneficial to remove alternative cellular energy sources from the blood including FAs. Insulin stimulates the synthesis of triglycerides, in which three FAs are attached to a glycerol backbone for storage. While FFAs can be used as an energy source by the majority cells in the body, most cell types are unable to break down triglycerides. The liver has only a very small capacity to breakdown triglycerides through lipolysis [[11](#_ENREF_11)]. Although the liver plays a role in lipid metabolism, adipose tissue is the primary regulator of blood lipid levels. As a result, it was also necessary to include a simple representation of adipose tissue in the model for it to provide valid blood FFA and triglyceride concentrations.

FFA uptake through the FATP proteins is included through two terms corresponding to passive concentration dependent uptake and the insulin dependent pumping of FFAs into the cytoplasm. Triglyceride release as very low density lipoproteins (VLDL) is included along with a term representing triglyceride uptake from the blood.

### Glucose Uptake and Output

#### Glucose uptake by GLUT2

**_
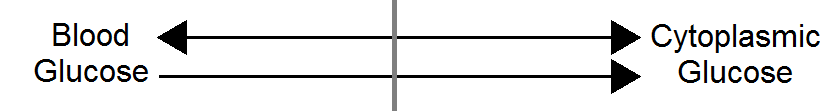
_**

The major glucose transporter in liver GLUT2 [[12](#_ENREF_12)]. Two terms represent pumping and diffusion of glucose into the cytoplasm. Simple uni- and bi- direction MM equations are used to represent these terms respectively. GLUT2 has a higher K_M_ value than other common glucose transporters allowing rapid uptake by hepatocytes when blood glucose levels rise.

$$\frac{v_{pump}*G_{B}}{\left( K_{M}^{pump}+G_{B} \right)}+\frac{v_{diff}*\left( G_{B}-G_{C} \right)}{\left( K_{M}^{diff}+G_{B}+G_{C} \right)}$$

$K_{M}^{pump}=17mM$ [[5](#_ENREF_5)],$K_{M}^{diff}=17mM$ [[5](#_ENREF_5)]

#### Glucokinase

**
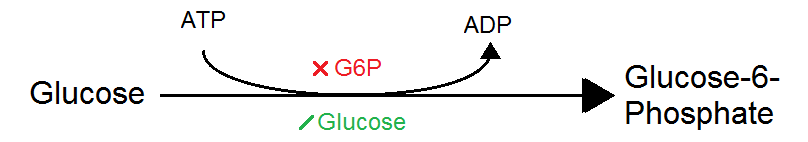
**

Glucokinase (GK) mediates the conversion of glucose to G6P taking a phosphate from ATP in the process. GK is allosterically dependent on the concentration of various molecules, either directly or indirectly via GK regulatory protein (GKRP). GKRP competitively binds to GK and moves the enzyme into the nucleus when glucose concentrations are low or when the concentrations of glycolysis intermediates such as F-6-P and F-1,6-P are high. G6P has a direct inhibitory effect. These dependences ensure increased uptake when glucose concentrations are high or when increased glycolysis is required to meet cellular energetic requirements. In the model, the effects glucose on GKRP (causing the release of GK) are included with constants taken from experimental data. Furthermore, the allosteric inhibition by G6P is included. F-6-P and F-1,6-P are not represented as independent variables in the model and their inhibitory effects (through GKRP) could not be included directly. However, conversion between G6P and F-6-P is rapid relative to the processes occurring in the model and so the two can be considered to be in constant equilibrium. In the model, a single G6P dependent inhibition term represents the allosteric inhibition of both G6P and F-6-P. The allosteric inhibition of F-1,6-P could not be included.

$$\frac{v_{gk}*{G_{c}}^{n_{free}}}{\left( {K_{M}^{gkrp}}^{n_{free}}+{G_{c}}^{n_{free}} \right)}*\frac{{G_{c}}^{n_{g}}}{\left( {K_{M}^{G}}^{n_{g}}+{G_{c}}^{n_{g}} \right)}*\frac{ATP}{\left( K_{M}^{ATP}+ATP \right)}*\left( 1-\frac{{G6P}^{n_{inh}}}{\left( {K_{i}^{G6P}}^{n_{inh}}+{G6P}^{n_{inh}} \right)} \right)$$

$K_{M}^{G}$= 7.5mM [[13](#_ENREF_13), [14](#_ENREF_14)], $K_{M}^{gkrp}$= 15mM [[13](#_ENREF_13), [15](#_ENREF_15)]

$n_{free}$= 2 [[13](#_ENREF_13), [15](#_ENREF_15)], $n_{g}$ = 1.4 [[13](#_ENREF_13), [15](#_ENREF_15)]

$K_{i}^{G6P}$= 240µM - [[16](#_ENREF_16), [17](#_ENREF_17)]

$K_{M}^{ATP}$= 240µM – a fairly wide range of values measured in the literature (e.g. 140µM [[18](#_ENREF_18)], 410µM [[14](#_ENREF_14)]). The value chosen for model is roughly in the middle of these. However since the values are much lower than the average ATP concentration under all but severe pathological conditions, small changes in the K_M_ value will not affect results significantly.

$n_{inh}$= 4 – As discussed, the activity of GK is strongly inhibited both directly by G6P and indirectly by intermediates of glycolysis such as F6P [[19](#_ENREF_19)]. F6P is not included in the model as an independent variable. However, conversion between F6P and G6P occurs rapidly relative to other processes included in the model. Therefore, the two can be expected to remain in constant equilibrium and a single allosteric dependence is included to represent both. A high n_inh_ of 4 is used to provide strong allosteric inhibition when G6P (/F6P) concentrations rise and a rapid reduction in inhibition when the concentration falls [[9](#_ENREF_9)].

#### G6Pase

**
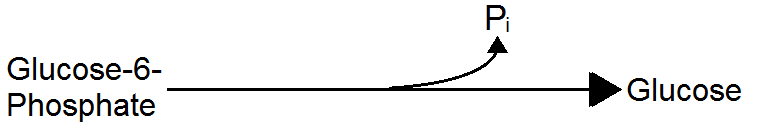
**

G6P is converted to glucose by the enzyme G6Pase releasing the phosphate. No strong allosteric or hormonal regulation has been demonstrated for this enzyme.

$$\frac{v_{G6Pase}*G6P}{K_{M}^{G6P}+G6P}$$

$K_{m}^{G6P}$ = 2.41mM [[20](#_ENREF_20), [21](#_ENREF_21)]

### Glycogen Synthesis and Breakdown

In order to control the rates of glycogenesis and glycogenolysis, insulin and glucagon bind to surface receptors causing a downstream cascade which determines the phosphorylation state, and hence activity, of glycogen synthase (GS) and glycogen phosphorylase (GP). In the model, a hormone dependent maximum activity is calculated for each enzyme before multiplying by MM-type substrate dependences.

#### Glycogen Synthase


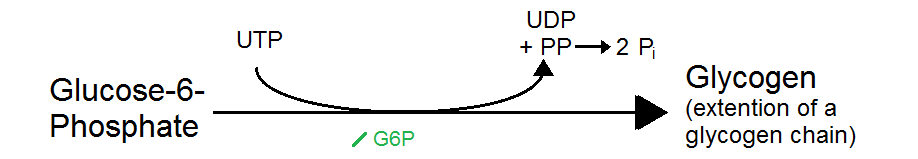


G6P is converted to glucose-1-phosphate (G1P) before binding with a uridine triphosphate (UTP) molecule to give UDP-glucose (and pyrophosphate (PP) which is rapidly broken down to give two phosphates (P_i_)). Glycogen synthase, the rate limiting enzyme in glycogen synthesis, uses this UDP-glucose to extend a glycogen chain. As well as the hormonal regulation, glycogen synthase is allosterically activated by an increased G6P concentration.

$$K_{syn}^{max}=\frac{\left( Ins+K_{IS} \right)}{\left( Glgn+K_{LS} \right)}$$

$$\frac{v_{syn}*K_{syn}^{max}*{G6P}^{n_{syn}}}{\left( {G6P}^{n_{syn}}+{K_{m}^{G6P}}^{n_{syn}} \right)}*\frac{UTP}{K_{M}^{UTP}+UTP}$$

$K_{m}^{UTP}$ = 48µM [[22](#_ENREF_22), [23](#_ENREF_23)]

$K_{IS}$ = 13.33pM, $K_{LS}$ = 62.5pM, $K_{M}^{G6P}$= 50µM, $n_{syn}$ = 4 set such that the model simulations match experimental data from [[24-30](#_ENREF_24)] for plasma concentrations of key glucose metabolism molecules throughout a daily feeding cycle [[24](#_ENREF_24)], for hepatic glycogen and plasma concentrations after a mixed meal [[27](#_ENREF_27)], and for average glycogen and hepatic glucose metabolism intermediate concentrations [[25](#_ENREF_25), [26](#_ENREF_26), [28](#_ENREF_28), [29](#_ENREF_29)] along with simulations from the model by Hetherington *et al.* [[9](#_ENREF_9), [31](#_ENREF_31)] (see S2 Text). A low K_M_ value but high hill coefficient ($n_{syn}$) was used for the G6P dependence. This rapidly reduces glycogen synthesis when G6P concentrations fall below their metabolically normal range due to the dependence on G6P both as a substrate (via G1P and UDP glucose) and as an allosteric activator (glycogen synthase activity nearly doubles in the presence of high concentrations of G6P compared with no G6P [[32](#_ENREF_32)]), whilst ensuring that the rate of synthesis is primarily determined by the effects of hormones under metabolically normal conditions. When G6P dependences that were less steep but reached saturation at higher G6P concentrations were tested, glycogen stores failed to empty when simulating insulin resistance.

#### Glycogen Phosphorylase


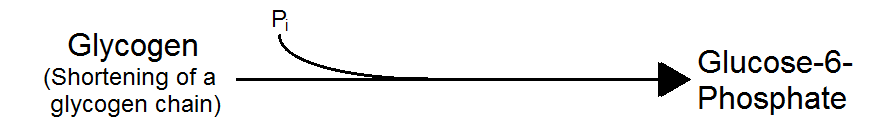


Glycogen phosphorylase depolymerises glycogen adding a phosphate to give G1P. This G1P is then rapidly converted to G6P by phosphoglucomutase.

$$K_{phos}^{max}=\frac{\left( Glgn+k_{LP} \right)}{\left( Ins+k_{IP} \right)}$$

$$\frac{{v_{brk}*K}_{Phos}^{max}*{Gly}^{n_{brk}}}{\left( {Gly}^{n_{brk}}+{K_{m}^{Gly}}^{n_{brk}} \right)}*\frac{Phos}{K_{M}^{phos}+Phos}$$

$K_{M}^{Gly}-$100mM (units of glucose), $K_{M}^{phos}$= 4000µM [[33](#_ENREF_33)] – for the active form of glycogen phosphorylase

$K_{IP}$ = 26.66pM, $K_{LP}$ = 45pM, $n_{brk}$= 4 – set such that the simulated data matches experimental data from [[24-30](#_ENREF_24)] for plasma concentrations of key glucose metabolism molecules throughout a daily feeding cycle [[24](#_ENREF_24)], for hepatic glycogen and plasma concentrations after a mixed meal [[27](#_ENREF_27)], and for average glycogen and hepatic glucose metabolism intermediate concentrations [[25](#_ENREF_25), [26](#_ENREF_26), [28](#_ENREF_28), [29](#_ENREF_29)] along with simulations from the model by Hetherington *et al.* [[9](#_ENREF_9), [31](#_ENREF_31)] (see S2 Text).

### Gluconeogenesis and Glycolysis

The rates of gluconeogenesis and glycolysis are strongly affected by the concentrations of insulin and glucagon (e.g. [[30](#_ENREF_30)]). As with glycogenesis and glycogenolysis, when calculating the rates of the glycolytic and gluconeogenic processes a maximum enzyme activity is first calculated based on the blood hormone concentrations. This is then multiplied by MM-type substrate dependences.

#### Glycolysis 1: G6P to GADP

**
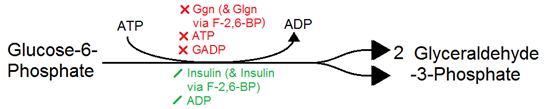
**

G6P is broken down to two glyceraldehyde-3-phosphate (GADP) molecules via a sequence of enzymes. The rate limiting enzyme in this conversion is phosphofructokinase (PFK) which is highly allosterically regulated. It is also the only unidirectional enzyme in the chain, with the reverse conversion mediated by fructose-bisphosphatase (FBPase). PFK requires the removal of a phosphate molecule from ATP, releasing ADP. The enzyme is allosterically activated by ADP and inhibited by ATP such that the rate of glycolysis is increased or slowed depending on the energy (ATP) requirements of the cell. The effects of ATP and ADP are included with K_M_, activation and inhibition constants from the literature. The subsequent enzyme in the chain, fructose-bisphosphate aldolase splits its substrate, fructose-1,6-bisphosphate into one GADP molecule and one dihydroxyacetone phosphate molecule, which is converted into a second GADP.

In addition, PFK is allosterically inhibited by GADP and phosphoenolpyruvate, products further down the glycolysis chain. It is also inhibited by intermediates of the citrate cycle, citrate and malate. As a result, glycolysis is not only slowed when ATP levels are high but also when there is a surplus of substrate for the citrate cycle. Of these effectors, only GADP is included as an independent variable in the model and, therefore, the inhibition of the molecules further down cannot be directly included. Instead, the inhibition of GADP included in the model is assumed to represent the inhibition of all glycolytic intermediates. Finally, PFK is also allosterically activated by fructose-2,6-bisphosphate (F-2,6-BP). The concentration of F-2,6-BP is predominantly determined by the action of insulin and glucagon, such that the PFK activity increases when plasma glucose, and hence insulin, concentrations are raised. As a result, although F-2,6-BP is also not included in the model as an independent variable, this allosteric dependence is included in the fitted hormone dependences.

$$K_{PFK}^{max}=\frac{\left( Ins+K_{IPFK} \right)}{\left( Glgn+K_{LPFK} \right)}$$

$$\frac{v_{PFK}*K_{PFK}^{max}*G6P}{K_{M}^{G6P}+G6P}*\frac{ATP}{K_{M}^{ATP}+ATP}*\frac{ADP}{K_{a}^{ADP}+ADP}\left( 1-\beta_{ATP}\frac{ATP}{K_{i}^{ATP}+ATP} \right)\left( 1-\beta_{GAdP}\frac{GADP}{K_{i}^{GADP}+GADP} \right)$$

$K_{IPFK}=2666.7pM$, $K_{LPFK}=1250pM$, - set so that the simulated data matches the data in references [[24-30](#_ENREF_24), [34](#_ENREF_34)] for plasma concentrations of glucose and lactate throughout a daily feeding cycle [[24](#_ENREF_24)], after a glucose load [[34](#_ENREF_34)] and after a mixed meal [[27](#_ENREF_27)], for average hepatic glucose metabolism intermediate concentrations including glucose, G6P, pyruvate, lactate, G3P and GADP [[25](#_ENREF_25), [26](#_ENREF_26), [28](#_ENREF_28), [29](#_ENREF_29)], and for the rates of glucose use in the various pathways after intake in the presence or absence of hormones [[30](#_ENREF_30)]. Additionally set so that the simulated data matches the data in references [[24](#_ENREF_24), [35](#_ENREF_35), [36](#_ENREF_36)] for the fatty acid and triglyceride concentrations throughout a mixed meal [[24](#_ENREF_24)] and for average concentrations of FFAs and triglycerides in individuals of varying weight and with varying insulin sensitivities [[35](#_ENREF_35), [36](#_ENREF_36)] due to the role of GADP/G3P in triglyceride synthesis (see S2 Text).

$K_{M}^{G6P}$= 5 µM – Since PFK doesn’t act directly from G6P, a K_M_ value couldn’t be taken from the literature. A very low value was used based on the observation that the rate of glycolysis does not increase when the glucose concentration is increased in insulin resistant patients [[34](#_ENREF_34)]. This suggests that in metabolically normal individual’s glycolysis increases due to the effects of insulin rather than increased substrate when plasma glucose concentrations are raised.

$K_{M}^{ATP}$= 42.5µM [[37](#_ENREF_37)], $\beta_{ATP}$= 1, $K_{a}^{ADP}$= 83.6μM [[37](#_ENREF_37), [38](#_ENREF_38)], $K_{i}^{ATP}$= 2.1mM [[37](#_ENREF_37), [38](#_ENREF_38)], $\beta_{GADP}=0.75$, $K_{i}^{GADP}$= 20.7 µM [[39](#_ENREF_39)]

#### Glycolysis 2: GADP to Lactate/Pyruvate


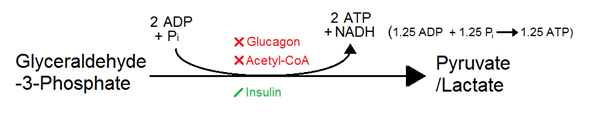


In the second half of glycolysis, each GADP molecule is converted to pyruvate (and lactate). Since conversion between pyruvate and lactate is reversible and relatively rapid, they are represented by a single variable in the model. Here we assume a constant pyruvate to lactate ratio. *In vivo*, the ratio is altered under conditions which effect the NAD:NADH ratio such as after heavy ethanol intake. Therefore, if in future work the model is used to study the metabolism of ethanol (or other drugs) across the sinusoid, modifications would be required to represent pyruvate and lactate as separate variables and to include NAD/NADH as separate variables rather than just through their effects on ATP concentrations.

The rate limiting enzyme in the conversion of GADP to pyruvate is Pyruvate Kinase (PK). Both the phosphate contained in the GADP and an additional free inorganic phosphate are combined with ADP molecules to produce two ATP molecules in this part of glycolysis. One ATP molecule is released by phosphoglycerate kinase and the second by pyruvate kinase. Additionally an NAD+ molecules is converted to NADH. Note that because 2 GADP molecules are produced per glucose in the previous stage of glycolysis, when considering the glycolysis of each glucose molecule, 4 ATP, 2 NADH and 2 pyruvate/lactate molecules in this stage.

NADH can produce a theoretical maximum of 3 ATP when entering the electron transport chain. However, a ratio of closer to 2.5 ATP per NADH is achieved for mitochondrial NADH. In the case of PK, the NADH is cytosolic and cannot cross the mitochondrial membrane. Instead a shuttle reactor of NADH_cyto_ + NAD^+^_mito_-> NADH_mito_ + NAD^+^_cyto_ occurs to transport NADH into the mitochondria which requires the consumption of 1 ATP molecule. Here a relatively low rate of 1.25 ATP per cytosolic NADH is assumed to account for these difficulties in transport and further inefficiency in the use of NADH. Pyruvate kinase is allosterically inhibited by acetyl-CoA.

$$K_{PK}^{max}=\frac{\left( Ins+K_{IPK} \right)}{\left( Glgn+K_{LPK} \right)}$$

$$\frac{v_{PK}*K_{PK}^{max}*GADP}{K_{m}^{GADP}+GADP}*\frac{ADP}{K_{M}^{ADP}+ADP}\left( 1-\beta_{allos}\frac{aCoA}{K_{i}^{aCoA}+aCoA} \right)$$

$K_{IPK}=1066.6pM$, $K_{LPK}=500pM, K_{m}^{GADP}$ = 250µM – set so that the simulated data matches the data in references [[24-30](#_ENREF_24), [34](#_ENREF_34)] for plasma concentrations of glucose and lactate throughout a daily feeding cycle [[24](#_ENREF_24)], after a glucose load [[34](#_ENREF_34)] and after a mixed meal [[27](#_ENREF_27)], for average hepatic glucose metabolism intermediate concentrations including glucose, G6P, pyruvate, lactate, G3P and GADP [[25](#_ENREF_25), [26](#_ENREF_26), [28](#_ENREF_28), [29](#_ENREF_29)], and for the rates of glucose use in the various pathways after intake in the presence or absence of hormones [[30](#_ENREF_30)]. Additionally set so that the simulated data matches the data in references [[24](#_ENREF_24), [35](#_ENREF_35), [36](#_ENREF_36)] for the fatty acid and triglyceride concentrations throughout a mixed meal [[24](#_ENREF_24)] and for average concentrations of FFAs and triglycerides in individuals of varying weight and with varying insulin sensitivities [[35](#_ENREF_35), [36](#_ENREF_36)] due to the role of GADP/G3P in triglyceride synthesis (see S2 Text).

$K_{M}^{ADP}$ = 240µM [[40](#_ENREF_40)], $K_{i}^{aCoA}$ = 30µM [[41](#_ENREF_41)], $\beta_{allos}$ = 0.8 [[41](#_ENREF_41)]

#### Gluconeogenesis 1: Lactate/Pyruvate to GADP (Glyceroneogenesis)

**
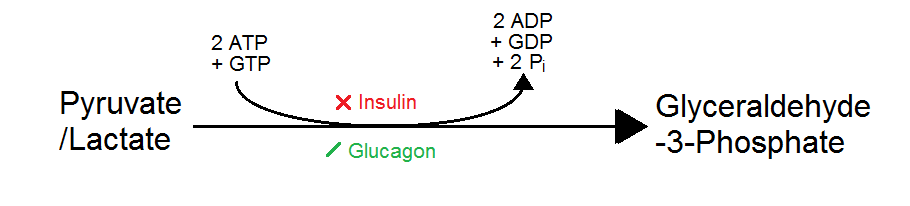
**

The conversion of pyruvate to GADP is highly energy intensive, requiring the removal of phosphates from 2 ATPs and a GTP. The rate limiting enzyme in this conversion is phosphoenolpyruvate carboxykinase (PEPCK) which hydrolyses GTP to provide a phosphate, allowing the production of phosphoenolpyruvate. PEPCK is not known to be strongly allosterically regulated.

$$K_{PEPCK}^{max}=\frac{\left( Glgn+k_{LPEPCK} \right)}{\left( Ins+k_{IPEPCK} \right)}$$

$$\frac{v_{PEPCK}*K_{PEPCK}^{max}*Lac}{K_{M}^{Lac}+Lac}*\frac{ATP}{K_{M}^{ATP}+ATP}*\frac{GTP}{K_{M}^{GTP}+GTP}$$

$K_{IFBP}=2266.6$, $K_{LFBP}=1062.5$, $K_{M}^{Lac}$= 500mM – set so that the simulated data matches the data in references [[24-30](#_ENREF_24), [34](#_ENREF_34)] for plasma concentrations of glucose and lactate throughout a daily feeding cycle [[24](#_ENREF_24)], after a glucose load [[34](#_ENREF_34)] and after a mixed meal [[27](#_ENREF_27)], for average hepatic glucose metabolism intermediate concentrations including glucose, G6P, pyruvate, lactate, G3P and GADP [[25](#_ENREF_25), [26](#_ENREF_26), [28](#_ENREF_28), [29](#_ENREF_29)], and for the rates of glucose use in the various pathways after intake in the presence or absence of hormones [[30](#_ENREF_30)]. Additionally set so that the simulated data matches the data in references [[24](#_ENREF_24), [35](#_ENREF_35), [36](#_ENREF_36)] for the fatty acid and triglyceride concentrations throughout a mixed meal [[24](#_ENREF_24)] and for average concentrations of FFAs and triglycerides in individuals of varying weight and with varying insulin sensitivities [[35](#_ENREF_35), [36](#_ENREF_36)] due to the roles of GADP/G3P in triglyceride synthesis and pyruvate in lipogenesis (see S2 Text).

$K_{M}^{ATP}$= 10 µM – set very low since, although ATP is required for the conversion of lactate to GADP, the ATP is required by enzymes which, under normal conditions, are not rate-limiting. Using a low K_M_ value means that large drops in ATP concentration will inhibit the process but variation in the physiological range will not affect the rate.

$K_{M}^{GTP}$= 64µM - [[42](#_ENREF_42)]

#### Gluconeogenesis 2: GADP to G6P


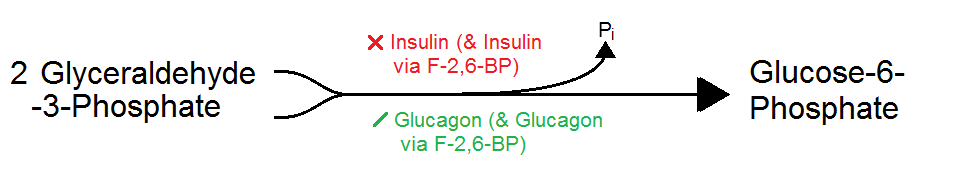


The rate limiting enzyme in the conversion of two GADP molecules to G6P is fructose-1,6-bisphosphatase (FBPase). FBPase is allosterically inhibited by F-2,6-BP. The F-2,6-BP concentration is primarily determined by the action of insulin and glucagon, such that hepatic glucose production is reduced when plasma glucose (and hence insulin) concentrations are high. Therefore, although F-2,6-BP is not included as an independent variable, its allosteric effects are included in the fitted hormone dependences.

$$K_{FBP}^{max}=\frac{\left( Glgn+k_{LFBP} \right)}{\left( Ins+k_{IFBP} \right)}$$

$$\frac{v_{FBP}*K_{FBP}^{max}*GADP}{K_{m}^{GADP}+GADP}$$

$K_{IFBP}=2666.6$, $K_{LFBP}=1250$, $K_{m}^{GADP}$= 250µM – set so that the simulated data matches the data in references [[24-30](#_ENREF_24), [34](#_ENREF_34)] for plasma concentrations of glucose and lactate throughout a daily feeding cycle [[24](#_ENREF_24)], after a glucose load [[34](#_ENREF_34)] and after a mixed meal [[27](#_ENREF_27)], for average hepatic glucose metabolism intermediate concentrations including glucose, G6P, pyruvate, lactate, G3P and GADP [[25](#_ENREF_25), [26](#_ENREF_26), [28](#_ENREF_28), [29](#_ENREF_29)], and for the rates of glucose use in the various pathways after intake in the presence or absence of hormones [[30](#_ENREF_30)]. Additionally set so that the simulated data matches the data in references [[24](#_ENREF_24), [35](#_ENREF_35), [36](#_ENREF_36)] for the fatty acid and triglyceride concentrations throughout a mixed meal [[24](#_ENREF_24)] and for average concentrations of FFAs and triglycerides in individuals of varying weight and with varying insulin sensitivities [[35](#_ENREF_35), [36](#_ENREF_36)] due to the roles of GADP/G3P in triglyceride synthesis and pyruvate in lipogenesis (see S2 Text).

### Acetyl-CoA Production

#### Pyruvate Oxidation

**
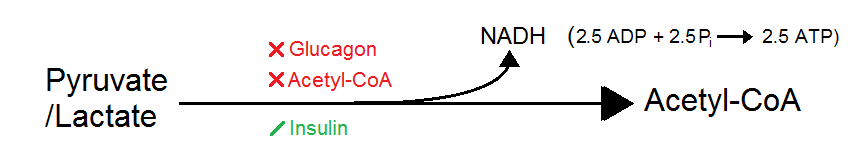
**

The conversion of pyruvate to acetyl-CoA is mediated by the pyruvate dehydrogenase complex (PDHC). PDHC is allosterically inhibited by acetyl-CoA ensuring that excessive acetyl-CoA does not enter the citrate cycle causing mitochondrial stress. PDHC also converts a mitochondrial NAD+ to NADH allowing for the production of 2.5 ATP molecules through the electron transport chain.

$$K_{PDH}^{max}=\left( 1+\frac{Ins}{{Ins}_{ref}^{asyn}}-\frac{Glcgn}{{Glcgn}_{ref}^{asyn}} \right)$$

$$\frac{v_{asyn}*K_{PDH}^{max}*Lac}{K_{M}^{Lac}+Lac}\left( 1-\frac{ACoA}{ACoA+k_{i}^{CoA-inhib}} \right)$$

$k_{i}^{CoA-inhib}$= 35 µM [[43](#_ENREF_43)]

$K_{M}^{Lac}$= 540, ${Ins}_{ref}^{asyn}$= 1.33nM, ${Glcgn}_{ref}^{asyn}$= 375pM - set so that the simulated data matches the data in references [[24-30](#_ENREF_24), [34-36](#_ENREF_34), [44](#_ENREF_44)] for plasma concentrations of key glucose and lipid metabolism molecules throughout a daily feeding cycle [[24](#_ENREF_24)], after a glucose load [[34](#_ENREF_34)] and after a mixed meal [[27](#_ENREF_27)], for average hepatic glucose metabolism intermediate concentrations including acetyl-CoA, pyruvate, glucose, G6P, lactate, G3P and GADP [[25](#_ENREF_25), [26](#_ENREF_26), [28](#_ENREF_28), [29](#_ENREF_29)], for average concentrations of FFAs and triglycerides in individuals of varying weight and with varying insulin sensitivities [[35](#_ENREF_35), [36](#_ENREF_36)], and for the rates of glucose use in the various pathways after intake in the presence or absence of hormones [[30](#_ENREF_30), [44](#_ENREF_44)] (see S2 Text).

#### β-oxidation

**
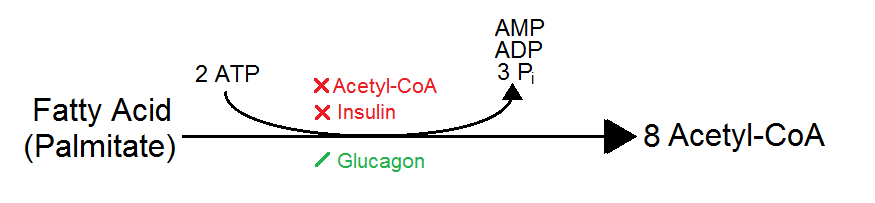
**

In β-oxidation, acetyl-CoA molecules are produced from the breakdown of fatty acids. *In vivo*, numerous fatty acids are found with varying chain lengths and with varying properties. To reduce the computation performed by the model, all fatty acids are synthesized from 8 acetyl-CoA molecules corresponding to palmitate, the most common fatty acid in animals. Initially the fatty acid is combined with a CoA molecule requiring the conversion of an ATP to AMP. An additional ATP molecule is required to transport the resulting acyl-CoA into the mitochondria where it is sequentially broken down. The transfer across the mitochondrial membrane, mediated by carnitine palmitoyltransferase 1 (CPT1), is considered to be rate-limiting in this process. β-oxidation is allosterically inhibited by its product acetyl-CoA to ensure a steady acetyl-CoA supply for the citrate cycle.

$$K_{\beta oxi}^{max}=\left( 1-\frac{Ins}{{Ins}_{ref}^{\beta oxi}}+\frac{Glcgn}{{Glcgn}_{ref}^{\beta oxi}} \right)$$

$$\frac{v_{\beta oxi}*K_{\beta oxi}^{max}*FA}{K_{M}^{FA}+FA}\left( \frac{ATP}{\left( K_{M}^{ATP}+ATP \right)} \right)\left( 1-\beta_{inh}\frac{ACoA}{ACoA+k_{i}^{CoA-inhib}} \right)$$

$K_{M}^{FA}$=5 µM – it is difficult to define a single K_M_ value for all fatty acids. K_M_ values for palmitate and a few additional fatty acids are provided below for fatty acid synthetase, the enzyme responsible for the initial activation of the fatty acid with CoA (from rats). All K_M_ values are in the range 1-10µM.

(5-8.6 µM Palmitate (16:0) – [[45](#_ENREF_45)]; 3.6-8.6 µM Palmitate (16:0) , 3-8.6 µM Oleate (18:1), 6.5-10 µM Arachidonate (20:4) [[46](#_ENREF_46)]; 2.78 µM Palmitate (16:0), 2.04 µM Palmitoleate (16:1), 1.39 µM Oleate (18:1), 2.22 µM Linoleate (18:2), 1.64 µM Linolenate (18:3) [[47](#_ENREF_47)])

$K_{M}^{ATP}$= 87µM [[48](#_ENREF_48)]

$k_{i}^{CoA-inhib}$= 47.8µM, [[49](#_ENREF_49)] $\beta_{inh}$= 0.4 [[49](#_ENREF_49)]

${Ins}_{ref}^{\beta oxi}$= 666.7pM, ${Glcgn}_{ref}^{\beta oxi}$= 875pM - set so that the simulated data matches the data in references [[24-30](#_ENREF_24), [34-36](#_ENREF_34), [44](#_ENREF_44), [50](#_ENREF_50), [51](#_ENREF_51)] for plasma concentrations of key glucose and lipid metabolism molecules throughout a daily feeding cycle [[24](#_ENREF_24)], after a glucose load [[34](#_ENREF_34)] and after a mixed meal [[27](#_ENREF_27)], for average hepatic glucose metabolism intermediate concentrations including acetyl-CoA, pyruvate, glucose, G6P, lactate, G3P and GADP [[25](#_ENREF_25), [26](#_ENREF_26), [28](#_ENREF_28), [29](#_ENREF_29)], for average concentrations of FFAs and triglycerides in individuals varying weight and with varying insulin sensitivities [[35](#_ENREF_35), [36](#_ENREF_36)], and for the rates of lipid and glucose use in the various pathways after intake in the presence or absence of hormones [[30](#_ENREF_30), [44](#_ENREF_44), [50](#_ENREF_50), [51](#_ENREF_51)] (see S2 Text).

### Oxidative phosphorylation and the energy molecules

#### Oxidative Phosphorylation/The Citrate Cycle

**
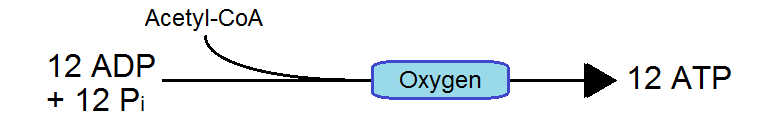
**

Rather than modelling the citrate cycle and electron transport chain in detail, they are represented by a single equation. For each acetyl-CoA molecule that enters the citrate cycle, 12 ATP molecules are produced from ADP and inorganic phosphate (based on the system working slightly below its maximum efficiency yield of 14 ATP per acetyl-CoA). An oxygen dependence is included based on the plasma oxygen concentration reaching the cells.

$$\frac{v_{ATPS}*ACoA}{K_{M}^{ACoA}+ACoA}*\frac{{Oxy}_{B}}{K_{M}^{{Oxy}_{B}}+{Oxy}_{B}}*\frac{Phos}{K_{M}^{Phos}+Phos}*\frac{ADP}{K_{M}^{ADP}+ADP}$$

$K_{M}^{ACoA}$= 0.4 µM – [[52](#_ENREF_52)] (measured in heart)

$K_{M}^{{Oxy}_{B}}$= 28mmHg for the oxygen concentration in the blood near the cell based on the value of 14mmHg in [[53](#_ENREF_53)] (doubled since it is estimated the the blood concentration of oxygen reaching cells is half of the average concentration in the sinusoid [[54](#_ENREF_54)])

$K_{M}^{Phos}$= 3830mM based on the average phosphate concentration in the model – The constant hasn’t been measured for humans in the literature and widely ranging values have been measured for other organisms (8.9mM [[55](#_ENREF_55)], 0.55mM [[56](#_ENREF_56)], ~10mM [[57](#_ENREF_57)]). However, when simulating normal conditions, less than a 5% change in phosphate concentration is seen throughout the day so ATP production is not expected to be strongly affected by phosphate concentration.

$K_{M}^{ADP}$= 410 µM – not measured in human or other mammals, based on data for thermophilic Bacillus PS3 [[55](#_ENREF_55)].

#### Nucleoside Diphosphate Kinases

Nucleoside diphosphate kinases (NDKs) mediate the exchange of phosphate groups between various nucleoside di-(and tri-) phosphates. K_M_ values were taken from the literature whilst the rate constants were fitted to average values for the 3 sets of di- and tri- phosphates included in the model.


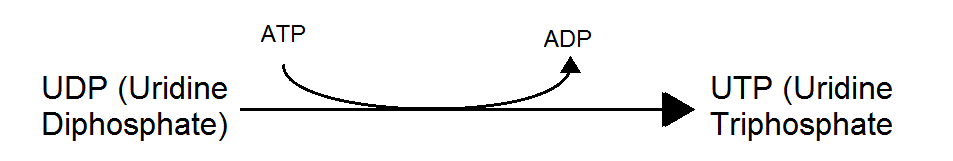

$$v_{NDKG}\left( \frac{ATP*GDP}{\left( K_{M}^{ATP}+ATP \right)\left( K_{M}^{GDP}+GDP \right)}-\frac{ADP*GTP}{\left( K_{M}^{ADP}+ADP \right)\left( K_{M}^{GTP}+GTP \right)} \right)$$

**
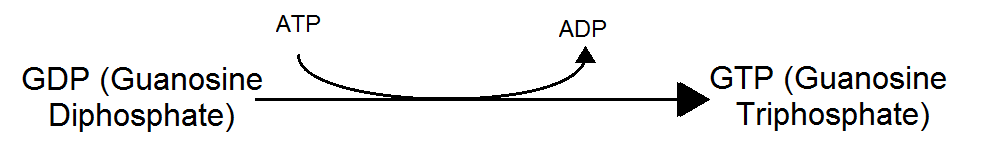
**

$$v_{NDKU}\left( \frac{ATP*UDP}{\left( K_{M}^{ATP}+ATP \right)\left( K_{M}^{UDP}+UDP \right)}-\frac{ADP*UTP}{\left( K_{M}^{ADP}+ADP \right)\left( K_{M}^{UTP}+UTP \right)} \right)$$

$K_{M}^{ATP}$= 290µM (200-380µM [[58](#_ENREF_58)]), $K_{M}^{GDP}$= 33.5µM (31-36 [[59](#_ENREF_59)]), $K_{M}^{ADP}$= 24µM [[58](#_ENREF_58)], $K_{M}^{GTP}$= 120µM [[58](#_ENREF_58)], $K_{M}^{UDP}$= 175µM (160-190 [[59](#_ENREF_59)]), $K_{M}^{UTP}$ = 21.5mM (16-27mM [[60](#_ENREF_60)])

#### Adenosine Kinase

Adenosine kinase (AK) mediates the bi-directional transfer of a phosphate from ATP to AMP providing two ADP. K_M_ values were taken from the literature whilst the rate constant was fitted to experimental data for average hepatic ATP, AMP and ADP concentrations

**
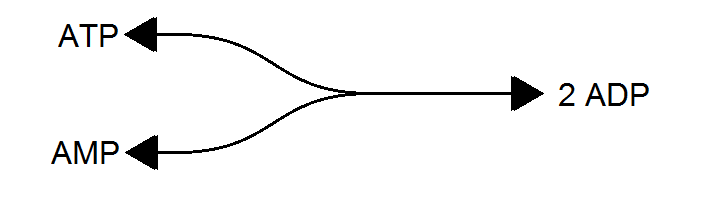
**$v_{AK}\left( \frac{ATP*AMP}{\left( K_{M}^{ATP}+ATP \right)\left( K_{M}^{AMP}+AMP \right)}-\frac{{ADP}^{2}}{\left( {K_{M}^{ADP}}^{2}+{ADP}^{2} \right)} \right)$

$K_{M}^{ATP}$= 90µM [[61](#_ENREF_61)], $K_{M}^{AMP}$= 80µM [[61](#_ENREF_61)], $K_{M}^{ADP}$= 110µM [[61](#_ENREF_61)]

#### Additional ATP Use

##### Cellular ATP usage term

ATP is consumed by processes other than glucose and lipid metabolism in hepatocytes. A single MM-type equation was introduced to represent this consumption of ATP. The rates of ATP production and use were fitted so that the average ATP, ADP and inorganic phosphate concentrations matched that measured in [[28](#_ENREF_28)]. Additionally, Ainscow and Brand performed a study in which they determined the relative rates of various processes under conditions of rapid glycogen breakdown in cultured hepatocytes [[44](#_ENREF_44)]. Under these conditions, 10% of G6P derived from glycogen breakdown entered glycolysis but was not released as lactate (46% overall entered glycolysis). Of this 10%, the vast majority would be expected to have entered the citrate cycle. This allows an estimation of the rate of use of glucose for ATP production. Lastly, Mandarino *et al.* studied the rates of glucose disposal and glucose oxidation and β-oxidation in muscle cells experiencing different insulin concentrations [[30](#_ENREF_30)]. While the liver derives a higher percentage of its energy from plasma FFAs that muscle, this gives an idea of the effects of hormonal stimulation (see S2 Text).

$$\frac{v_{atpuse}*ATP}{\left( K_{M}^{ATP}+ATP \right)}$$

$K_{M}^{ATP}$ = 2500 – roughly equal to the average ATP concentration in pericentral compartment.

##### Control of cellular phosphate levels

The rate of cytosolic phosphate production or usage in glucose metabolism is strongly dependent on the feeding state and, in initial simulations, the concentration varied massively over time. As a result an additional term was added representing the control of the cytosolic phosphate concentration.

$$v_{con}\left( P_{i}-{ref}_{P_{i}} \right)$$

$v_{con}$= 0.1, ${ref}_{P_{i}}$= 4.15mM fitted so that the average phosphate concentration matched that in [[28](#_ENREF_28)] and didn’t fluctuate significantly from this value under different feeding conditions.

### Lipogenesis

**
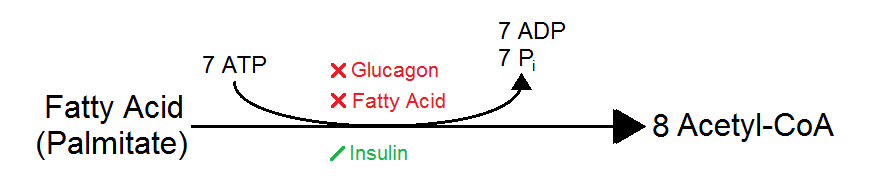
**

An acetyl-CoA molecule is converted to malonyl-CoA by acetyl-CoA carboxylase (ACC) before combining with a second acetyl-CoA molecule under the influence of fatty acid synthase (FAS). FAS then mediates the addition of subsequent malonyl-CoA molecules forming the fatty acid chain. Of the two ACC isoforms, only ACC1 contributes to lipogenesis. The malonyl-CoA produced by mitochondrial ACC2 is physically separated from FAS under normal physiological conditions and instead is involved in the allosteric inhibition of β-oxidation [[62](#_ENREF_62)]. In the model, all fatty acids are produced from 8 acetyl-CoA molecules corresponding to palmitate. Fatty acid production is allosterically inhibited by various fatty acid molecules.

$$K_{lgen}^{max}=\left( 1+\frac{Ins}{{Ins}_{ref}^{lgen}}-\frac{Glcgn}{{Glcgn}_{ref}^{lgen}} \right)$$

$$\frac{v_{lgen}*K_{lgen}^{max}*ACoA}{K_{M}^{ACoA}+ACoA}*\frac{ATP}{K_{M}^{ATP}+ATP}\left( 1-\frac{FA}{FA+k_{i}^{FA-inhib}} \right)$$

$K_{M}^{ACoA}$ = 58µM [[63](#_ENREF_63)] for ACC. FAS is requires acetyl-CoA (in the initial step an acetyl-CoA molecules is joined with a malonyl-CoA molecule before additional malonyl-CoA molecules are added to the fatty acid chain). However, the K_M_ value for FAS is very low such that under normal physiological conditions further increase in acetyl-CoA will not increase the rate. Instead, fatty acid synthase is rate limited by the malonyl-CoA produced by ACC [[64](#_ENREF_64), [65](#_ENREF_65)].

$K_{m}^{FA-inhib}$ = 300µM, ${Ins}_{ref}^{lgen}$ = 8000pM, ${Glcgn}_{ref}^{lgen}$ = 875pM – set so that the simulated data matches experiment data for plasma concentrations of key glucose and lipid metabolism molecules throughout a daily feeding cycle [[24](#_ENREF_24), [66](#_ENREF_66)], after a glucose load [[34](#_ENREF_34)] and after a mixed meal [[27](#_ENREF_27)], for average hepatic glucose metabolism intermediate concentrations including acetyl-CoA, pyruvate, glucose, G6P, lactate, G3P and GADP [[25](#_ENREF_25), [26](#_ENREF_26), [28](#_ENREF_28), [29](#_ENREF_29)], for average plasma concentrations of FFAs and triglycerides in individuals varying weight and with varying insulin sensitivities [[35](#_ENREF_35), [36](#_ENREF_36), [67](#_ENREF_67)], for the hepatic triglyceride concentration [[68](#_ENREF_68)], and for the effects of insulin and glucagon on the rate of lipogenesis and activity of acetyl-CoA carboxylase (in adipose tissue rather than liver) [[69-71](#_ENREF_69)] (see S2 Text).

$K_{M}^{ATP}$= 120µM [[63](#_ENREF_63)].

### Triglyceride Synthesis and Breakdown

#### Triglyceride Synthesis


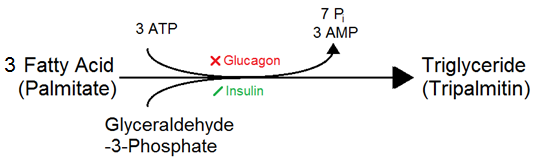


Each fatty acid must be joined with a coenzyme A (CoA) to form acyl-CoA before being used for triglyceride synthesis, requiring the conversion of 3 ATP molecules to AMP. An acyl-CoA is attached to a glycerol backbone derived from glycerol-3-phosphate (G3P) by G3P-acyltransferase forming a monoacylglyceride. Since conversion between G3P and GADP is rapid and reversible they are represented by a single combined variable in the model. Another two acyl-CoA molecules are sequentially added by acyltransferases forming diacylglycerides followed by triglycerides. In the model these conversions are currently represented by one equation rather than as individual reactions. Triglyceride synthesis is promoted by insulin and inhibited by glucagon. Note that diacylglycerides are also used to produce various phospholipids not currently included in the model.

$$K_{tsyn}^{max}=\left( 1+\frac{Ins}{{Ins}_{ref}^{tsyn}}-\frac{Glcgn}{{Glcgn}_{ref}^{tsyn}} \right)$$

$$\frac{v_{tsyn}*K_{tsyn}^{max}*FA}{K_{M}^{FA}+FA}*\frac{GADP}{K_{M}^{GADP}+GADP}$$

$K_{M}^{\mathrm{GADP}}$ = 460µM [[72](#_ENREF_72)].

$\mathrm{Glcgn}_{\mathrm{ref}}^{\mathrm{TGsyn}}$ = 500pM, $\mathrm{Ins}_{\mathrm{ref}}^{\mathrm{TGsyn}}$ = 1.066nM – set so that the simulated data matches experiment data for plasma concentrations of key glucose and lipid metabolism molecules throughout a daily feeding cycle [[24](#_ENREF_24), [66](#_ENREF_66)], after a glucose load [[34](#_ENREF_34)] and after a mixed meal [[27](#_ENREF_27)], for average hepatic glucose metabolism intermediate concentrations including acetyl-CoA, pyruvate, glucose, G6P, lactate, G3P and GADP [[25](#_ENREF_25), [26](#_ENREF_26), [28](#_ENREF_28), [29](#_ENREF_29)], for average plasma concentrations of FFAs and triglycerides in individuals varying weight and with varying insulin sensitivities [[35](#_ENREF_35), [36](#_ENREF_36), [67](#_ENREF_67)], for the hepatic triglyceride concentration [[68](#_ENREF_68)], and for the effects of hormones on the rate of triglyceride synthesis and the activities of various triglyceride synthesis enzymes (in adipose tissue rather than liver) [[69](#_ENREF_69), [73](#_ENREF_73)] (see S2 Text).

$K_{M}^{\mathrm{FA}}$ = 645µM – As discussed for β-oxidation, it is not possible to measure a single K_M_ value for all fatty acids. The value used is high relative to the low cellular fatty acid concentration such that the dependence of the rate on the concentration is almost linear. This ensures that triglyceride synthesis increases and falls when hepatic fatty acid concentrations fluctuate throughout the day.

#### Lipolysis

**
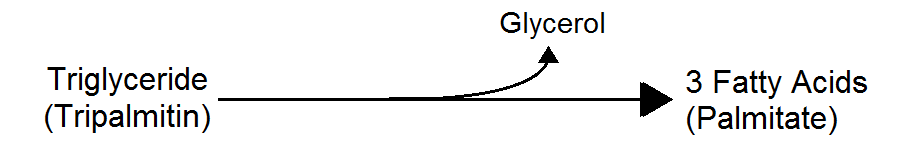
**

Each triglyceride is broken down to 3 fatty acids and a glycerol molecule by a sequence of lipases (rate limited by triacylglycerol lipase). Hepatic lipolysis is stimulated by glucagon and suppressed by insulin.

$$K_{lply}^{max}=\left( 1-\frac{Ins}{{Ins}_{ref}^{lply}}+\frac{Glcgn}{{Glcgn}_{ref}^{lply}} \right)$$

$$\frac{v_{lply}*K_{lply}^{max}*TG}{K_{M}^{TG}+TG}$$

$K_{M}^{TG}$ = 50.715mM – It is difficult to base a K_M_ value on experimentally measured values since numerous triglycerides exist and widely ranging K_M_ values are measured for both different triglycerides and in different studies of the same triacylglycerol (e.g. tripalmitin (three palmitic acids attached to a glycerol backbone) [[74](#_ENREF_74), [75](#_ENREF_75)]). A relatively high value was chosen relative to the average hepatic concentrations such that increases in hepatic triglyceride levels cause increased lipolysis. However, since the rate of lipolysis is very slow in hepatocytes, hepatic lipolysis is not a major determinant of the hepatic or plasma triglyceride concentrations and has very little effect on the rest of hepatic metabolism. When triglyceride breakdown is required this predominantly occurs in adipose tissue.

${Ins}_{ref}^{lply}$= 1.067nM, ${Glcgn}_{ref}^{lply}$ = 625pM – set so that the simulated data matches experiment data for plasma concentrations of key glucose and lipid metabolism molecules throughout a daily feeding cycle [[24](#_ENREF_24), [66](#_ENREF_66)], after a glucose load [[34](#_ENREF_34)] and after a mixed meal [[27](#_ENREF_27)], for average plasma concentrations of FFAs and triglycerides in individuals varying weight and with varying insulin sensitivities [[35](#_ENREF_35), [36](#_ENREF_36), [67](#_ENREF_67)], for the hepatic triglyceride concentration [[68](#_ENREF_68)], and for the effects of hormonal regulation on triglyceride synthesis [[69](#_ENREF_69)] (see S2 Text).

#### Glycerol Kinase

**
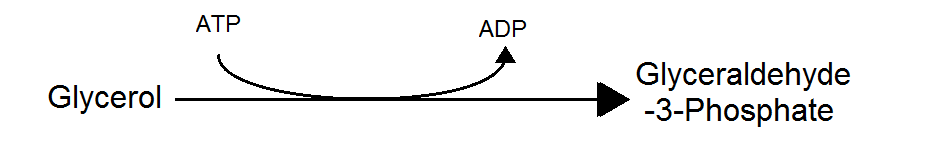
**

Glycerol is converted G3P by glycerol kinase requiring the conversion of ATP to ADP. G3P is then rapidly and reversible converted to GADP.

$$\frac{v_{gconv}*Glycerol}{K_{M}^{Glycerol}+Glycerol}*\frac{ATP}{K_{M}^{ATP}+ATP}$$

$K_{M}^{Gly}$= 41µM – (36-46µM for rat [[76](#_ENREF_76)])

$K_{M}^{ATP}$= 15µM – no data for human, ranging from 6µM to 3mM for different bacteria (BRENDA enzyme database). Changes in the glycerol concentration are very small compared to the glucose, lactate and lipid concentrations so changes in the parameters defining this term do not have a large effect on the system as a whole.

### Membrane Transport

#### Lactate Output/Uptake


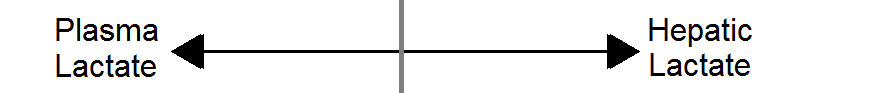


Lactate uptake/output is represented through a diffusion term dependent on the relative blood and cytoplasmic lactate concentrations.

$$\frac{v_{lact}*\left( {Lac}_{B}-{Lac}_{C} \right)}{\left( K_{M}^{Lac}+{Lac}_{B}+{Lac}_{C} \right)}$$

$K_{M}^{Lac}$ = 1.2mM – Roughly equal to the average lactate concentration. $K_{M}^{Lac}$ and $v_{lact}$ were set so that the simulated data matches the experimental data for plasma concentrations of glucose and lactate throughout a daily feeding cycle [[24](#_ENREF_24)], after a glucose load [[34](#_ENREF_34)] and after a mixed meal [[27](#_ENREF_27)], for average hepatic glucose metabolism intermediate concentrations including glucose, G6P, pyruvate, lactate, G3P and GADP [[25](#_ENREF_25), [26](#_ENREF_26), [28](#_ENREF_28), [29](#_ENREF_29)], and for the rates of glucose use in the various pathways after intake in the presence or absence of hormones [[30](#_ENREF_30)] (see S2 Text).

#### Fatty Acid Output/Uptake


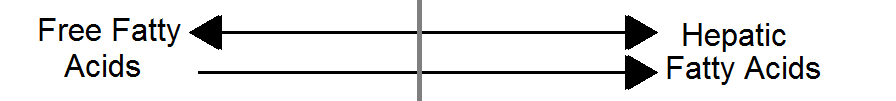


Fatty acid uptake is complex and not fully understood [[77](#_ENREF_77)]. It is known fatty acid transport proteins FATP2 and FATP5 play an important role with uptake reduced in mice lacking either protein [[77-79](#_ENREF_77)]. Additionally, overexpression of scavenger receptor CD36 (known to occur in NAFLD) promotes increased uptake [[80-82](#_ENREF_80)]. Knockout of liver-type fatty acid binding protein also reduces fatty acid uptake [[83](#_ENREF_83)]. Passive unfacilitated diffusion also contributes to hepatic uptake although this process is relatively slow [[84](#_ENREF_84), [85](#_ENREF_85)]. Once FFAs enter the liver they are rapidly bound to a CoA molecule to form acyl-CoA preventing efflux. Cellular fatty acids are rapidly utilized in β-oxidation or attached to a glycerol backbone to form di- and triacylglycerol. As a result, the hepatic concentration is low (<50µM).

Insulin causes increased fatty acid uptake, stimulating fatty acid transport proteins [[86-88](#_ENREF_86)]. In the model, fatty acid uptake is represented by two terms. Firstly, an insulin-dependent, unidirectional uptake term corresponding to transport proteins actively pulling fats into the cell. Secondly, a non-hormone dependent, bidirectional term accounting for both unfacilitated diffusion and bidirectional facilitated diffusion. Given that the cellular concentration is very small compared to the plasma concentration, these both act strongly as uptake terms, even when simulating insulin resistance.

$$\frac{v_{active}*F{FA}_{B}}{\left( K_{M}^{active}+F{FA}_{B} \right)}\left( 1+\frac{Ins}{{Ins}_{ref}^{active}} \right)+\frac{v_{diff}*\left( F{FA}_{B}-F{FA}_{C} \right)}{\left( K_{M}^{diff}+F{FA}_{B}+{FFA}_{C} \right)}$$

$K_{M}^{active}$ = 2µM, $K_{M}^{diff}$ = 200µM, ${Ins}_{ref}^{active}$ = 21.333pM – set so that the simulated data matches experiment data for plasma concentrations of key glucose and lipid metabolism molecules throughout a daily feeding cycle [[24](#_ENREF_24), [66](#_ENREF_66)], after a glucose load [[34](#_ENREF_34)] and after a mixed meal [[27](#_ENREF_27)], for average hepatic glucose metabolism intermediate concentrations including acetyl-CoA, pyruvate, glucose, G6P, lactate, G3P and GADP [[25](#_ENREF_25), [26](#_ENREF_26), [28](#_ENREF_28), [29](#_ENREF_29)], for average plasma concentrations of FFAs and triglycerides in individuals varying weight and with varying insulin sensitivities [[35](#_ENREF_35), [36](#_ENREF_36), [67](#_ENREF_67)], for the rates of lipid and glucose use in the various pathways after intake in the presence or absence of hormones [[30](#_ENREF_30), [44](#_ENREF_44), [50](#_ENREF_50), [51](#_ENREF_51)], for the hepatic triglyceride concentration [[68](#_ENREF_68)], and for the effects of hormones on the rate of lipogenesis and release, and the activities of various lipogenic enzymes (in adipose tissue rather than liver) [[69](#_ENREF_69), [73](#_ENREF_73)] (see S2 Text).

#### Triglyceride Output/Uptake


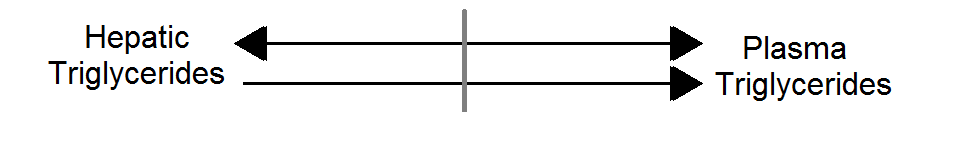


Two terms represent movement of triglycerides between the cytoplasm and the blood stream. The first term represents the production and release of VLDL. Liver is the major organ responsible for packaging triglycerides into VLDL such that this is the main triglyceride output from the cell [[89](#_ENREF_89)]. It should be noted that, in the model, once triglycerides are in the blood, free triglycerides along with triglycerides contained in lipoproteins are represented by a single variable. The second term represents very slow uptake of triglycerides from transporter molecules in the plasma and the slow output in forms other than VLDL. It has been shown in numerous species that hepatocytes are able to uptake triglycerides from plasma lipoproteins, although the proteins involved in the transport have yet to be fully characterised [[90-92](#_ENREF_90)]. Therefore, a simple bidirectional Hill function with K_M_ value based on the average plasma and hepatic triglyceride concentration was used. Since the hepatic triglyceride concentration is much higher than the plasma concentration (healthy livers store up to 5% fat), a conversion constant ${TG}_{ref}$ was included to ensure this bi-directions transfer term did not result in constant output.

$$-\frac{v_{VLDL}*{TG}_{C}}{\left( K_{M}^{VLDL}+{TG}_{C} \right)}+\frac{v_{diff}*\left( {TG}_{B}-\frac{{TG}_{C}}{{TG}_{ref}} \right)}{\left( K_{M}^{diffi}+{TG}_{B}+\frac{{TG}_{C}}{{TG}_{ref}} \right)}$$

$K_{M}^{VLDL}$ = 33.81mM, $K_{M}^{diffi}$= 1mM, ${TG}_{ref}$=33.81 –Roughly equal to experimental data for average plasma [[35](#_ENREF_35), [36](#_ENREF_36), [67](#_ENREF_67)], and hepatic TG concentrations [[68](#_ENREF_68)]. Due to the slow rate of lipogenesis, short-term variations in the hepatic concentration have little effect on the rest of metabolism.

#### Glycerol Output/Uptake


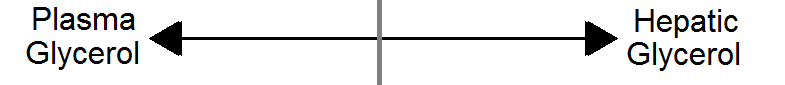


Glycerol released primarily by WAT during lipolysis is cleared from the blood by hepatocytes. Uptake is represented by a simple diffusion term.

$$\frac{v_{Glyct}*\left( {Glyc}_{B}-{Glyc}_{C} \right)}{\left( K_{M}^{Glyc}+{Glyc}_{B}+{Glyc}_{C} \right)}$$

$K_{M}^{Glyc}$ = 270µM (250-402µM measured in rat [[76](#_ENREF_76)]) – In simulations, glycerol kinase is rate-limiting in glycerol clearance rather than uptake such that moderate changes in $K_{M}^{Glyc}$ do not have a large effect on the overall rate of uptake.

## Adipose Tissue, Muscle and Dietary Inputs and Outputs

In the model, all dietary carbohydrates enter the blood stream as glucose. Dietary lipids are broken down to fatty acids before being absorbed by the enterocytes of the intestinal wall. Short to medium chain fatty acids can enter directly into the portal vein. Longer chain fatty acids are converted back into triglycerides and released into the bloodstream as chylomicrons via the lymph system, although some long chain fatty acids have been shown to enter directly to the portal vein. Triglycerides are broken down to FFAs predominantly in adipose tissue. Similarly, adipose tissue plays a large role in regulating FFA concentration by removing them from the blood stream, esterifying them and storing as triglycerides. These triglycerides are converted back to fatty acids before being released into the blood when required between meals.

Given that the focus of this report is on hepatic metabolism rather than the gut or adipose tissue, only a very minimal representation of these lipid metabolism processes was included. This was fitted to experimental data for lipid levels in metabolically normal and insulin resistant individuals. Lipids enter the body compartment of the model as fatty acids. A single equation then calculates the synthesis of triglycerides in the gut (and other organs in the body excluding liver). Two equations represent adipose lipogenesis and adipose lipolysis. A final equation represents insulin dependent FFA uptake by muscle and other body cells. Since a separate adipose tissue compartment was not included in the model, adipose lipid storage could not be included. Instead, the adipose and muscle equations act directly on the blood concentrations of glucose, FFAs and triglycerides. The constants in these equations were fitted by eye such that the FFA and triglyceride concentrations matched those measured throughout the day for healthy patients by Daly *et al* [[24](#_ENREF_24)] and such that the average FFA and triglyceride concentrations matched those measured for diabetic and non-diabetic patients by Sindelka *et al.* [[35](#_ENREF_35)] and Berndt *et al.* [[36](#_ENREF_36)].

### Adipose and Gut Equations

#### Adipose De Novo Synthesis

De novo fatty acid synthesis is represented by a single equation in which 4 glucose molecules are converted to a fatty acid.

$$\frac{v_{dnWAT}*G_{B}}{K_{M}^{G_{B}}+G_{B}}\left( 1+\frac{Ins}{{Ins}_{ref}^{dnWAT}}-\frac{Glcgn}{{Glcgn}_{ref}^{dnWAT}} \right)$$

$K_{M}^{G_{B}}$= 4.5mM, ${Ins}_{ref}^{dnWAT}$=1.87nM, ${Glcgn}_{ref}^{dnWAT}$=250pM

#### Triglyceride Synthesis and Lipolysis

When representing gut triglyceride synthesis and adipose lipolysis, time constants needed to be added to slow the effect of hormones. This is to compensate for the fact that adipose triglyceride storage was not included in the model. In initial model building, the rates of triglyceride synthesis and lipolysis were set to depend only the current plasma insulin and glucagon concentrations (as with the rest of the equations in the model). However, this led to the rapid breakdown of triglycerides and a large spike in fatty acid concentration as soon as glucose concentrations dropped between meals. Instead, i*n vivo*, the fatty acid concentration increases more steadily over time between meals, and fatty acids continue to be released until the next meal (or lipid stores begin to empty). The difference between the simulated data and the experimental data is likely to be accounted for by the lack of adipose triglyceride storage in the model. To compensate for this, rate constants with a slower dependence on the plasma hormone concentration were used. Once this had been implemented, the simulated data more accurately matched those measured by Daly *et al* [[24](#_ENREF_24)], Sindelka *et al.* [[35](#_ENREF_35)] and Berndt *et al.* [[36](#_ENREF_36)] without the need to include adipose lipid storage.

##### Non-Hepatic Triglyceride Synthesis

Three FFAs attached to a G3P backbone corresponding to half of a blood glucose molecule to form a triglyceride.

$If\left( \frac{Glcgn}{{Glcgn}_{ref}^{trisyn}}-v_{G} \right)>0$; then: $\frac{dv_{G}}{dt}=\frac{\left( \frac{Glcgn}{{Glcgn}_{ref}^{trisyn}}-v_{G} \right)}{{\tau_{G}}^{up}}$; else: $\frac{dv_{G}}{dt}=\frac{\left( \frac{Glcgn}{{Glcgn}_{ref}^{trisyn}}-v_{G} \right)}{{\tau_{G}}^{down}}$

$If\left( \frac{Ins}{{Ins}_{ref}^{trisyn}}-v_{I} \right)>0$; then: $\frac{dv_{I}}{dt}=\frac{\left( \frac{Ins}{{Ins}_{ref}^{trisyn}}-v_{I} \right)}{{\tau_{I}}^{up}}$; else: $\frac{dv_{I}}{dt}=\frac{\left( \frac{Ins}{{Ins}_{ref}^{trisyn}}-v_{I} \right)}{{\tau_{I}}^{down}}$

$$v_{trisynH}=v_{trisyn}*\left( 1+v_{I}-v_{G} \right)$$

$$\frac{v_{trisynH}*G_{B}}{K_{M}^{G_{B}}+G_{B}}\frac{FFA}{K_{M}^{FFA}+FFA}$$

$K_{M}^{G_{B}}$=10mM, $K_{M}^{FFA}$=645mM, ${Ins}_{ref}^{trisyn}$=800pM, ${Glcgn}_{ref}^{trisyn}$=37.5pM

${\tau_{I}}^{up}$ =1000s^-1^, ${\tau_{I}}^{down}$ =15000s^-1^, ${\tau_{G}}^{up}$=10000s^-1^, ${\tau_{G}}^{down}$=700s^-1^

##### Adipose Lipolysis

A triglyceride is removed from the blood and broken down into three fatty acids and a glycerol.

$If\left( \frac{Glcgn}{{Glcgn}_{ref}^{lipoly}}-v_{G} \right)>0$; then: $\frac{dv_{G}}{dt}=\frac{\left( \frac{Glcgn}{{Glcgn}_{ref}^{lipoly}}-v_{G} \right)}{{\tau_{G}}^{up}}$; else: $\frac{dv_{G}}{dt}=\frac{\left( \frac{Glcgn}{{Glcgn}_{ref}^{lipoly}}-v_{G} \right)}{{\tau_{G}}^{down}}$

$If\left( \frac{Ins}{{Ins}_{ref}^{lipoly}}-v_{I} \right)>0$; then: $\frac{dv_{I}}{dt}=\frac{\left( \frac{Ins}{{Ins}_{ref}^{lipoly}}-v_{I} \right)}{{\tau_{I}}^{up}}$; else: $\frac{dv_{I}}{dt}=\frac{\left( \frac{Ins}{{Ins}_{ref}^{lipoly}}-v_{I} \right)}{{\tau_{I}}^{down}}$

$$v_{lipolyH}=v_{lipolysis}*\left( 1-v_{I}+v_{G} \right)$$

$$\frac{v_{lipolyH}*{TG}_{B}}{K_{M}^{{TG}_{B}}+{TG}_{B}}$$

$K_{M}^{{TG}_{B}}$= 2mM, ${Ins}_{ref}^{lipolysis}$= 800pM, ${Glcgn}_{ref}^{lipolysis}$=37.5pM

${\tau_{I}}^{up}$=1000s^-1^, ${\tau_{I}}^{down}$=15000s^-1^, ${\tau_{G}}^{up}$=10000s^-1^, ${\tau_{G}}^{down}$=700s^-1^

### Input

Glucose and FFA inputs of any form can be inputted to the model. For simulating an input sequence roughly equivalent to that of a daily feeding cycle spiked glucose and FFA inputs with a period of 4 hours was used.

$v_{input}*\sin^{6} \left( \frac{pi}{2(hours)} \right)$ -> Spiked inputs with 4 hour period

For a moderate diet, inputs were based on the average meal values in Daly *et al.* [[24](#_ENREF_24)].

$v_{input}\left( Glucose \right)$=19.275μM/s - (78.1g/cycle assuming 5L of blood in the body compartment)

$v_{input}(FFA)$ =3.5μM/s - (20.1g/cycle assuming 5L of blood in the body compartment and the molecular mass of palmitate).

### Consumption Terms (Muscle and Body cells)

In the model glucose is consumed by cells according to a simple hill function with a low K_M_ constant such that, under normal conditions, glucose is absorbed at a relatively constant rate by muscle and other body cells. In reality, other organs show hormone dependent uptake. However, since liver is the major determinant of plasma glucose concentration, this effect was not included.

$$\frac{v_{Gbuse}*G_{B}}{K_{M}^{G_{B}}+G_{B}}$$

$K_{M}^{G_{B}}$=1mM, $v_{G_{B}use}$= 4.93µM/s

The rate of fatty acid consumption elsewhere in the body was represented by a similar term with the inclusion of an insulin dependence.

$$\frac{v_{FFAuptake}*FFA}{K_{M}^{FFA}+FFA}\left( \frac{\left( Ins+k_{Iup} \right)}{\left( Glgn+k_{Lup} \right)} \right)$$

$K_{M}^{FFA}$= 100 µM, $k_{Iup}$= 250pM, $k_{Lup}$= 125pM, $v_{FFAuptake}$= 0.982µM/s

## Oxygen and Hormone Inputs and Consumption

### Hormone Release by the Pancreas

The representation of pancreatic hormone release developed by Hetherington *et al.* was used to calculate the rate of release of glucagon and insulin into the blood [[9](#_ENREF_9)]. In this, insulin is only released when the blood glucose is above a threshold. Above this threshold, insulin is released according to a hill function acting on the logarithm of the glucose concentration relative to a reference concentration. Similarly, glucagon is only released when the blood glucose concentration is below the threshold. In this case glucagon is released according to a hill function acting on the inverse logarithm of the blood glucose concentration.

$$if G_{B}<G_{ref, Glucagon release =}\frac{1}{\tau_{glgn}}\frac{{\ln\frac{G_{ref}}{G_{B}}}^{n_{glgn}}}{\left( {K_{m}^{glgn}}^{n_{glgn}}+{\ln\frac{G_{ref}}{G_{B}}}^{n_{glgn}} \right)}$$

$$if G_{B}>G_{ref, insulin release =}\frac{1}{\tau_{ins}}\frac{{\ln\frac{G_{ref}}{G_{B}}}^{n_{ins}}}{\left( {K_{m}^{ins}}^{n_{ins}}+{\ln\frac{G_{ref}}{G_{B}}}^{n_{ins}} \right)}$$

Constants as in [[9](#_ENREF_9)].

### Hormone Degradation Across the Sinusoid

In the model the hormones are degraded at a constant rate (per unit of hormone) as blood passes through the sinusoid. Experimentally, the concentration of glucagon has been measured to fall by around 50% between the blood entering the sinusoid and the blood exiting it [[93](#_ENREF_93)]. To match this, the rate of degradation of glucagon was set to 0.03858 per unit of glucagon per second. *In vivo*, the insulin concentration falls by 50% across the sinusoid between meals but by only 15% post-prandially [[93](#_ENREF_93)]. Since insulin is only released in the model when blood glucose levels are high, only the post-prandial rate of degradation was included and a rate of 0.01389 per unit of insulin per second was used. Therefore an increase in the insulin to glucagon ratio is seen as blood passes through the sinusoid.

### Oxygen Input and Consumption

A simple representation of oxygen input and consumption across the sinusoid is implemented in the model with a constant input of 1.35mmHg s^-1^ in the ‘body’ blood compartment and a constant output of 0.03525s^-1^ per unit of oxygen in each compartment of blood across sinusoid. This provides a gradient in oxygen concentration across the sinusoid falling from 65mmHG in the blood entering the proximal periportal compartment to 35mmHG in the blood leaving the distal pericentral compartment.

## Rate Constants and Zonation

Each term representing a pathway in the model contains a rate constant dictating the speed at which conversions occur. The rate constant for each process has a base-value (v_b_) which is then modified in each compartment according to the zonation of the process. These base-values for each process were set such that the plasma and average sinusoidal concentration of variables in the model matched the experimentally measured values (see Table 1). Where possible, experimental data showing the concentrations under different conditions (e.g. fed, starved, diabetic) or time series data looking at the variation in concentrations after feeding and during starvation were used.

To allow for the inclusion of zonated enzyme expression, these base values were next altered in each compartment according to whether the enzymes in each process are known to be up-regulated or down-regulated in that region of the sinusoid.

Zonation is known to be primary regulated by the blood oxygen concentration [[94](#_ENREF_94)]. As a result, the blood oxygen concentration is used to assign each compartment a value, z, dictating how periportal-like or pericentral-like the enzyme expression in that compartment is. The following function was used to define z.

$$If Oxy>46.5mmHg then (z=tanh \left( \frac{\left( Oxy-46.5 \right)}{12} \right)else (z=2*tanh\left( \frac{(oxy-46.5)}{24} \right)$$

This function is plotted in Fig 1 and was based on the data in Nauck *et al.* [[95](#_ENREF_95)] and Wolfle and Jungermann [[96](#_ENREF_96)] in which the concentrations of key periportal and pericentral enzymes were measured at a range of physiological oxygen concentration. A value of roughly z=1 is given at periportal oxygen concentrations (65-75mmHG) and a value of roughly z=-1 is given at pericentral oxygen concentrations (30-35mmHg). These were used as reference points for setting periportal-type and pericentral-type expression. As shown in the experimental studies [[95](#_ENREF_95), [96](#_ENREF_96)], further induction of periportal-type enzymes does not occur at oxygen concentrations above that seen by periportal cells. However, in hypoxic conditions, additional induction of pericentral-type enzymes and suppression of periportal-type enzymes does occur.


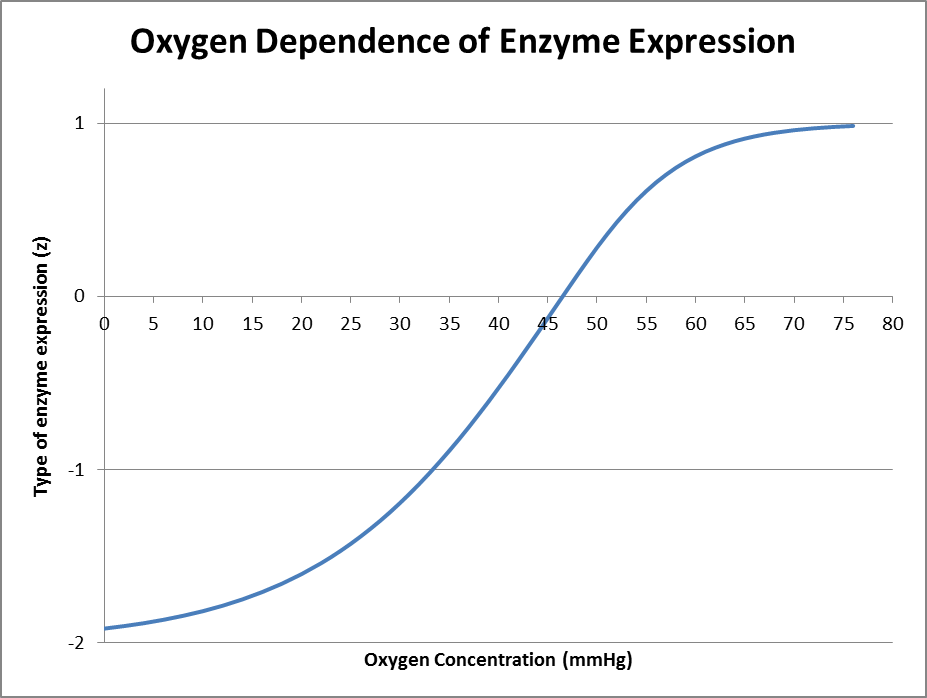


~Periportal Oxygen Concentration

~Perivenous Oxygen Concentration

Hypoxia

z=-1 – Perivenous Enzyme Expression

z=1 - Periportal Enzyme Expression

Fig 1: The dependence of enzyme expression type (pericentral -> periportal) on oxygen concentration in the model.

For a process where the base-value of the rate constant is $v_{b}$ and the experimentally measured ratio of periportal expression to pericentral expression is k_pp_:1, the altered rate constant in compartment $x$, ($vx$) is calculated by:

$$v_{x}=(1+z_{x}*k_{n})v_{b}$$

Where:

$$k_{n}=1-\frac{2*k_{pp}}{\left| k_{pp} \right|+1}$$

This equation gives the experimentally measured periportal: pericentral ratio between compartments with z=1 and z=-1 whilst leaving the average rate constant across the sinusoid unchanged at physiological oxygen concentrations. Table 2 shows the experimentally measured periportal to pericentral ratio in activity for the enzymes mediating each process along with the constant $k_{n}$ used in the model.

| Rate Constant | Base Value in Model, $v_{b}$ | Data Used For Fitting –  (See S2 Text for comparisons with experimental data) |
| --- | --- | --- |
| Glucose Uptake | $v_{pump}=118$ µM s^-1^,  $v_{diff}^{glu}=224$ µM s^-1^, | [[24-30](#_ENREF_24), [44](#_ENREF_44), [67](#_ENREF_67)] – fitted by eye to data for the concentrations in blood and in liver of various glucose metabolism molecules under different conditions.  [[24](#_ENREF_24)] – time series data for plasma concentrations throughout a daily meal cycle.  [[27](#_ENREF_27)] – time series data for glucose and glycogen concentrations after a single meal.  [[25](#_ENREF_25), [26](#_ENREF_26), [28](#_ENREF_28), [29](#_ENREF_29)] – hepatic concentrations of several energy metabolism intermediates.  [[30](#_ENREF_30), [44](#_ENREF_44)] – the relative rates at which lipids and glucose are oxidised and the relative rates of glucose release, glycolysis, lactate release and acetyl-CoA synthesis under conditions of glycogenolysis. Further comparison with the postprandial rates of glucose and fatty acid oxidation is presented in [[50](#_ENREF_50), [51](#_ENREF_51)] in S2 Text. |
| Glucokinase | $v_{gk}$ = 112 s^-1^ |  |
| G6Pase | $v_{G6Pase}$ = 370 µM s^-1^ |  |
| Glycogen Synthase | $v_{syn}$ = 55 µM s^-1^ |  |
| Glycogen Phosphorylase | $v_{brk}$=5 µM s^-1^ |  |
| Glycolysis 1 (PFK) | $v_{PFK}$ = 160 µM s^-1^ |  |
| Glycolysis 2 (PK) | $v_{PK}$ = 87 µM s^-1^ |  |
| Gluconeogenesis 1 (PEPCK) | $v_{PEPCK}$= 35 µM s^-1^ |  |
| Gluconeogenesis 2 (FBPase) | $v_{FBP}$=68 µM s^-1^ |  |
| Pyruvate Oxidation(PDHC) | $v_{asyn}$= 15 µM s^-1^ | – set using [[25](#_ENREF_25), [26](#_ENREF_26), [28](#_ENREF_28)] as a reference for the average cytosolic acetyl-CoA concentration, along with [[24-30](#_ENREF_24), [34](#_ENREF_34)] for the glucose and lactate concentrations under different conditions, [[24](#_ENREF_24), [35](#_ENREF_35), [36](#_ENREF_36)] for the FFA and triglyceride concentrations under different conditions and [[30](#_ENREF_30), [44](#_ENREF_44), [50](#_ENREF_50), [51](#_ENREF_51)] for the relative rates of glucose and fatty acid oxidation. |
| Β-oxidation | $v_{\beta oxi}$= 3.3 µM s^-1^ |  |
| ATP Production through the citrate cycle | $v_{ATPS}$=520 µM s^-1^ | – set such that the concentrations of the mono- di- and tri- phosphate molecules matched to the following average values/ranges:  Inorganic Phosphate – 3.81 (3.55-4.07)mM [[28](#_ENREF_28)];  ATP – 2.78 (2.71-2.85)mM [[28](#_ENREF_28)];  ADP – .885 (.794-.976)mM [[97](#_ENREF_97)];  AMP – .237 (.200-.272)mM [[28](#_ENREF_28)];  UTP – .285 (.255-.315)mM [[97](#_ENREF_97)];  UDP – .108 (.096-.120)mM [[97](#_ENREF_97)];  GTP – .277 (.266-.288)mM [[97](#_ENREF_97)];  GDP – .098 (.091-.105)mM [[97](#_ENREF_97)]  Rates of ATP production and consumption were estimated based on the data in [[30](#_ENREF_30), [44](#_ENREF_44)]. |
| Nucleoside Diphosphate kinases | $v_{NDKG}$=3000 µM s^-1^  $v_{NDKU}$=30 µM s^-1^ |  |
| Adenosine Kinase | $v_{AK}$= 100 µM s^-1^ |  |
| ATP consumption | $v_{atpuse}$ = 173 µM s^-1^ |  |
| Lipogenesis | $v_{lgen}$ = 5.5 µM s^-1^ | Overall lipogenesis (hepatic +WAT) was set such that the plasma concentrations and total lipogenesis rates matched to the data in [[24](#_ENREF_24), [27](#_ENREF_27), [66](#_ENREF_66), [67](#_ENREF_67), [69](#_ENREF_69)].  [[25](#_ENREF_25), [26](#_ENREF_26), [28](#_ENREF_28), [29](#_ENREF_29)] used as references for hepatic concentrations.  [[98](#_ENREF_98)] provides data for the relative contribution of liver to overall lipid metabolism. |
| Triglyceride Synthesis | $v_{\mathrm{TGsyn}}$= 10 µM s^-1^ | Set such that the plasma triglyceride and FFA concentration and overall (hepatic + WAT + gut etc.) triglyceride production and degradation matched data in [[24](#_ENREF_24), [27](#_ENREF_27), [30](#_ENREF_30), [35](#_ENREF_35), [36](#_ENREF_36), [67](#_ENREF_67), [69](#_ENREF_69), [73](#_ENREF_73)] as closely as possible. However, since adipose triglyceride storage is not included in the model, larger variation in plasma triglyceride concentration occurred in the simulated data throughout each intake/output cycle than is seen *in vivo.* Given that short term variations in plasma triglyceride concentration have little effect on hepatic metabolism due to the slow rate of triglyceride uptake and lipolysis, emphasis was placed on ensuring the FFA concentration matched the experimentally measured at each time point, whilst only the time-averaged plasma triglyceride value matched the experimental data. See S2 Text for further discussion. The contribution of liver to triglyceride synthesis was based on the data in [[98](#_ENREF_98)]. Hepatic triglyceride concentration set to match [[99](#_ENREF_99)]. |
| Lipolysis | $v_{lply}$= 0.085 µM s^-1^ |  |
| FFA Uptake | $v_{active}^{FFA}$=0.08µM s^-1^  $v_{diff}^{FFA}$= 1.2µM s^-1^ |  |
| Triglyceride Uptake/Output | $v_{VLDL}$= 0.3µM s^-1^  $v_{diff}^{TG}$= 0.4µM s^-1^ |  |
| Adipose Lipid Metabolism | $v_{dnWAT}$= .22µM s^-1^ |  |
|  | $v_{trisyn}$= 8.5µM s^-1^ |  |
|  | $v_{lipolysis}$= 2µM s^-1^ |  |
| Glycerol Kinase | $v_{gconv}$= 5 µM s^-1^ | The average blood glycerol concentration was matched to the data in [[24](#_ENREF_24)]. |
| Lactate Uptake | $v_{lact}$= 200 µM s^-1^ | Changes in lactate concentration are largely determined by the effects of hormonal and allosteric regulation on glycolysis. [[24-29](#_ENREF_24), [67](#_ENREF_67)] were used as reference values blood/hepatic concentrations of lactate and glycolysis intermediates. |
| Glycerol Uptake | $v_{Glyct}$= 100µM s^-1^ | Membrane transport is not considered rate limiting in the use of glycerol by hepatocytes. However, the plasma concentration was matched to the experimental data measured by Daly *et al.* [[24](#_ENREF_24)] |

Table 1. The baseline rate constants used in the model and the experimental data used for fitting.


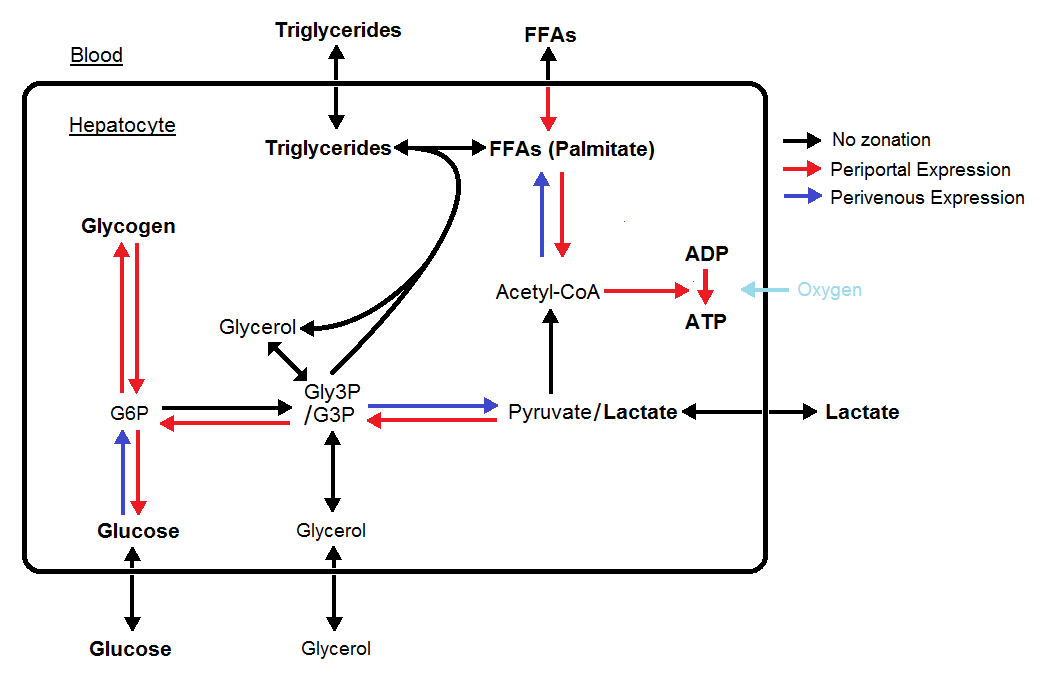


Fig 2. The zonation of enzymes as represented in the model.

(11).001 1.5

| Process | Periportal to pericentral ratio in the model. | Zonation of key enzymes (experimental) | References | Zonation Constant ($k_{n}$) k_n_=0 – no zonation  0<k_n_<1 – periportal  -1<k_n_<0 – pericentral |
| --- | --- | --- | --- | --- |
| Glucose Uptake  (Glucose -> G6P) | 1:2.5 | Glucokinase (GK)  1 : 1.5-3.5 | [[100-109](#_ENREF_100)] | $-0.429$ |
| Glucose Output  (G6P -> Glucose) | 1.9:1 | Glucose-6-Phosphatase (G6Pase)  2.3-1.5 : 1 | [[102](#_ENREF_102), [110-112](#_ENREF_110)] | $0.31$ |
| Glycogenesis  (G6P-> Glycogen) | 3:1 | Glycogen Synthase  Periportal – no quantitative data found | [[113](#_ENREF_113)] | 0.5 |
| Glycogenolysis  (Glycogen -> G6P) | 1:1 | Conflicting data in literature – perhaps dependent on feeding conditions. | [[113](#_ENREF_113), [114](#_ENREF_114)] | $0$ (no zonation) |
| Glycolysis 1  (Glucose –> GADP) | 1:1 | 6-Phosphofructo-kinase_L_ (PFK_L_)  1 : 1-1.3** | [[115](#_ENREF_115)]** reported as statistically insignificant difference between sections | $0$ (no zonation) |
| Glycolysis 2  (GADP –> Pyruvate/Lactate) | 1:2.1 | Pyruvate Kinase_L_ (PK_L_)  1 : 1.66-2.5 | Cited in [[93](#_ENREF_93)]  [[116](#_ENREF_116)] | $-0.355$ |
| Gluconeogenesis 2  (GADP -> Glucose) | 1.75:1 | Fructose-bisphosphatase (FBPase)  1.5-2:1 | [[100](#_ENREF_100), [101](#_ENREF_101), [117](#_ENREF_117)] | $0.273$ |
| Gluconeogenesis 1  (Pyruvate/Lactate –> GADP) | 2.4:1 | Phosphoenolpyruvate carboxykinase (PEPCK)  1.9-2.9 : 1 | Cited in [[93](#_ENREF_93)]  [[95](#_ENREF_95), [109](#_ENREF_109), [113](#_ENREF_113), [118-120](#_ENREF_118)] | $0.412$ |
|  |  | Pyruvate Carboxylase  1.72:1 | [[121](#_ENREF_121)] |  |
| Pyruvate Oxidation | 1:1 | Pyruvate dehydrogenase  1 : 1.69 (insignificant due to large variability, especially in the pericentral compartment) | [[121](#_ENREF_121)] | $0$ (no zonation) |
| Oxidative phosphorylation and the citrate cycle | 1.5:1 | Cristae volume:2.38:1 | [[122](#_ENREF_122)] | $0.2$ |
|  |  | Cristae Area: 2.05:1 | [[122](#_ENREF_122)] |  |
|  |  | Succinate Dehydrogenase:1.9:1  Malate Dehydrogenase 1.7:1 | [[123](#_ENREF_123)] |  |
|  |  | Cytochrome c oxidase >1:1 | [[124](#_ENREF_124)] |  |
| Lipogenesis | 1:1.6 | Acetyl-CoA Carboxylase  Fed : 1 : 1.58 (f), 1 : 1.64 (m).  Starved: 1 : 1.13 (f), 1 : 1.32 (m).  Refed: 1 : 1.61 (f), 1 : 1.60 (m). | [[125](#_ENREF_125)] | $-0.23$ |
|  |  | Fatty Acid Synthase  Fed 1 : 1.91 (f), 1 : 1.04* (m).  Starved: 1 : 1.26* (f), 1.27 : 1* (m).  Refed: 1 : 2.39 (f)1 : 1.34 (m). | [[126](#_ENREF_126)] |  |
|  |  | ATP-dependent dependent citrate lyase  Fed: 1 : 2.4 (f), 1 : 1.82 (m).  Starved: 1 : 1.47 (f), 1 : 1.64 (m).  Refed: 1 : 2.5 (f), 1 : 2.78 (m). | [[127](#_ENREF_127)] |  |
|  |  | G6P dehydrogenase,  6-phosphogluconate dehydrogenase, Malic enzyme, Isocitrate dehydrogenase, Alcohol dehydrogenase,  As a general trend 1 : >1 (pericentral) | For review see [[93](#_ENREF_93)] |  |
| β-oxidation | 1.6:1 | β-Hydroxybutyryl-CoA hydrogenase  Fed: 1.52 : 1 (f), 1.59 : 1 (m)  Starved: 1.58 : 1 (f), 1.80 : 1 (m) | [[125](#_ENREF_125), [128](#_ENREF_128)] | $0.23$ |
|  |  | Carnitine palmitoyltransferase-1  1.26 : 1 (in the absence of hormones) | [[129](#_ENREF_129)] |  |
|  |  | Liver fatty acid binding protein  1.6 : 1 | [[130](#_ENREF_130), [131](#_ENREF_131)] |  |
| Triglyceride Synthesis | 1:1 | No data demonstrating zonation in triglyceride synthesis enzymes found |  | $0$ (no zonation) |
| Triglyceride Breakdown | 1:1 | No data demonstrating zonation in lipolysis enzymes found |  | $0$ (no zonation) |
| Insulin Reception | 1:1.35 | Insulin Receptor proteins  1:2 estimated from *in vitro* test using physiological oxygen concentrations.  P.C. distribution demonstrated in vivo. | [[132](#_ENREF_132)] | $-0.15$ |
| Glucagon Reception | 1.35:1 | Glucagon Receptor Proteins  1-3.5:1 (for mRNA) | [[133](#_ENREF_133)] | $0.15$ |
| FFA membrane transport uptake | 1.5:1 | L-FABP expression - ~1.5:1 | [[134](#_ENREF_134)] | $0.2$ |

Table 2. The zonation of key enzymes in the model.

## Supplementary Material 1 References

1. Eipel, C., K. Abshagen, and B. Vollmar, *Regulation of hepatic blood flow: the hepatic arterial buffer response revisited.* World J Gastroenterol, 2010. **16**(48): p. 6046-57.

2. Davy, K.P. and D.R. Seals, *Total blood volume in healthy young and older men.* J Appl Physiol (1985), 1994. **76**(5): p. 2059-62.

3. Critchley, L.A. and J.A. Critchley, *A meta-analysis of studies using bias and precision statistics to compare cardiac output measurement techniques.* J Clin Monit Comput, 1999. **15**(2): p. 85-91.

4. Arias, B., Fausto, Jakoby, Schachter, Shafritz, *The Liver Biology and Pathology*. Third Edition ed. 1994, New York: Raven Press.

5. Johnson, J.H., et al., *The high Km glucose transporter of islets of Langerhans is functionally similar to the low affinity transporter of liver and has an identical primary sequence.* J Biol Chem, 1990. **265**(12): p. 6548-51.

6. Jung, C.Y. and A.L. Rampal, *Cytochalasin B binding sites and glucose transport carrier in human erythrocyte ghosts.* J Biol Chem, 1977. **252**(15): p. 5456-63.

7. Buschiazzo, H., J.H. Exton, and C.R. Park, *Effects of glucose on glycogen synthetase, phosphorylase, and glycogen deposition in the perfused rat liver.* Proc Natl Acad Sci U S A, 1970. **65**(2): p. 383-7.

8. Konig, M., S. Bulik, and H.G. Holzhutter, *Quantifying the Contribution of the Liver to Glucose Homeostasis: A Detailed Kinetic Model of Human Hepatic Glucose Metabolism.* Plos Computational Biology, 2012. **8**(6).

9. Hetherington, J., et al., *A composite computational model of liver glucose homeostasis. I. Building the composite model.* J R Soc Interface, 2012. **9**(69): p. 689-700.

10. Reshef, L., et al., *Glyceroneogenesis and the triglyceride/fatty acid cycle.* J Biol Chem, 2003. **278**(33): p. 30413-6.

11. Dolinsky, V.W., et al., *Regulation of the enzymes of hepatic microsomal triacylglycerol lipolysis and re-esterification by the glucocorticoid dexamethasone.* Biochem J, 2004. **378**(Pt 3): p. 967-74.

12. Zheng, Q., et al., *Glucose regulation of glucose transporters in cultured adult and fetal hepatocytes.* Metabolism, 1995. **44**(12): p. 1553-8.

13. Agius, L., *Glucokinase and molecular aspects of liver glycogen metabolism.* Biochemical Journal, 2008. **414**: p. 1-18.

14. Gloyn, A.L., et al., *Insights into the structure and regulation of glucokinase from a novel mutation (V62M), which causes maturity-onset diabetes of the young.* Journal of Biological Chemistry, 2005. **280**(14): p. 14105-14113.

15. Brocklehurst, K.J., R.A. Davies, and L. Agius, *Differences in regulatory properties between human and rat glucokinase regulatory protein.* Biochemical Journal, 2004. **378**: p. 693-697.

16. Ahn, K.J., et al., *Enzymatic properties of the N- and C-terminal halves of human hexokinase II.* Bmb Reports, 2009. **42**(6): p. 350-355.

17. Goward, C.R., et al., *The purification and characterization of glucokinase from the thermophile Bacillus stearothermophilus.* Biochem J, 1986. **237**(2): p. 415-20.

18. Heredia, V.V., et al., *Glucose-induced conformational changes in glucokinase mediate allosteric regulation: transient kinetic analysis.* Biochemistry, 2006. **45**(24): p. 7553-62.

19. van Schaftingen, E., et al., *The regulatory protein of liver glucokinase.* Adv Enzyme Regul, 1992. **32**: p. 133-48.

20. van Schaftingen, E. and I. Gerin, *The glucose-6-phosphatase system.* Biochem J, 2002. **362**(Pt 3): p. 513-32.

21. Henry-Vitrac, C., et al., *Contribution of chlorogenic acids to the inhibition of human hepatic glucose-6-phosphatase activity in vitro by Svetol, a standardized decaffeinated green coffee extract.* J Agric Food Chem, 2010. **58**(7): p. 4141-4.

22. Turnquist, R.L., T.A. Gillett, and R.G. Hansen, *Uridine diphosphate glucose pyrophosphorylase. Crystallization and properties of the enzyme from rabbit liver and species comparisons.* J Biol Chem, 1974. **249**(23): p. 7695-700.

23. Turnquist, R.L.H., R.G.; , *Uridine diphosphoryl glucose pyrophosphorylase*, in *The Enzymes, 3rd. Ed (Boyer, P.D., ed.)* 1973. p. 8.

24. Daly, M.E., et al., *Acute effects on insulin sensitivity and diurnal metabolic profiles of a high-sucrose compared with a high-starch diet.* Am J Clin Nutr, 1998. **67**(6): p. 1186-96.

25. Albe, K.R., M.H. Butler, and B.E. Wright, *Cellular Concentrations of Enzymes and Their Substrates.* Journal of Theoretical Biology, 1990. **143**(2): p. 163-195.

26. Rawat, A.K., *Effects of ethanol infusion on the redox state and metabolite levels in rat liver in vivo.* Eur J Biochem, 1968. **6**(4): p. 585-92.

27. Taylor, R., et al., *Direct assessment of liver glycogen storage by 13C nuclear magnetic resonance spectroscopy and regulation of glucose homeostasis after a mixed meal in normal subjects.* J Clin Invest, 1996. **97**(1): p. 126-32.

28. Veech, R.L., D. Veloso, and M.A. Mehlman, *Thiamin deficiency: liver metabolite levels and redox and phosphorylation states in thiamin-deficient rats.* J Nutr, 1973. **103**(2): p. 267-72.

29. Faupel, R.P., et al., *The problem of tissue sampling from experimental animals with respect to freezing technique, anoxia, stress and narcosis. A new method for sampling rat liver tissue and the physiological values of glycolytic intermediates and related compounds.* Arch Biochem Biophys, 1972. **148**(2): p. 509-22.

30. Mandarino, L.J., et al., *Effects of insulin infusion on human skeletal muscle pyruvate dehydrogenase, phosphofructokinase, and glycogen synthase. Evidence for their role in oxidative and nonoxidative glucose metabolism.* J Clin Invest, 1987. **80**(3): p. 655-63.

31. Sumner, T., et al., *A composite computational model of liver glucose homeostasis. II. Exploring system behaviour.* J R Soc Interface, 2012. **9**(69): p. 701-6.

32. Baskaran, S., et al., *Structural basis for glucose-6-phosphate activation of glycogen synthase.* Proc Natl Acad Sci U S A, 2010. **107**(41): p. 17563-8.

33. Assaf, S.A. and A.A. Yunis, *Physicochemical and Catalytic Properties of Crystallized Human Muscle Glycogen-Phosphorylase.* Annals of the New York Academy of Sciences, 1973. **210**(Feb9): p. 139-152.

34. Prando, R., et al., *Blood Lactate Behavior after Glucose-Load in Diabetes-Mellitus.* Acta Diabetologica Latina, 1988. **25**(3): p. 247-256.

35. Sindelka, G., et al., *Association of obesity, diabetes, serum lipids and blood pressure regulates insulin action.* Physiol Res, 2002. **51**(1): p. 85-91.

36. Berndt, J., et al., *Fatty acid synthase gene expression in human adipose tissue: association with obesity and type 2 diabetes.* Diabetologia, 2007. **50**(7): p. 1472-80.

37. Bruser, A., J. Kirchberger, and T. Schoneberg, *Altered allosteric regulation of muscle 6-phosphofructokinase causes Tarui disease.* Biochemical and Biophysical Research Communications, 2012. **427**(1): p. 133-137.

38. Bruser, A., et al., *Functional linkage of adenine nucleotide binding sites in mammalian muscle 6-phosphofructokinase.* J Biol Chem, 2012. **287**(21): p. 17546-53.

39. Mediavilla, D., I. Meton, and I.V. Baanante, *Purification and kinetic properties of 6-phosphofructo-1-kinase from gilthead sea bream muscle.* Biochim Biophys Acta, 2007. **1770**(4): p. 706-15.

40. Dombrauckas, J.D., B.D. Santarsiero, and A.D. Mesecar, *Structural basis for tumor pyruvate kinase M2 allosteric regulation and catalysis.* Biochemistry, 2005. **44**(27): p. 9417-29.

41. Weber, G., M.A. Lea, and N.B. Stamm, *Inhibition of pyruvate kinase and glucokinase by acetyl CoA and inhibition of glucokinase by phosphoenolpyruvate.* Life Sciences, 1967. **6**(22): p. 2441–2452.

42. Dharmarajan, L., et al., *Tyr235 of human cytosolic phosphoenolpyruvate carboxykinase influences catalysis through an anion-quadrupole interaction with phosphoenolpyruvate carboxylate.* FEBS J, 2008. **275**(23): p. 5810-9.

43. Kiselevsky, Y.V., S.A. Ostrovtsova, and S.A. Strumilo, *Kinetic characterization of the pyruvate and oxoglutarate dehydrogenase complexes from human heart.* Acta Biochim Pol, 1990. **37**(1): p. 135-9.

44. Ainscow, E.K. and M.D. Brand, *Top-down control analysis of ATP turnover, glycolysis and oxidative phosphorylation in rat hepatocytes.* Eur J Biochem, 1999. **263**(3): p. 671-85.

45. Kim, J.H., T.M. Lewin, and R.A. Coleman, *Expression and characterization of recombinant rat Acyl-CoA synthetases 1, 4, and 5. Selective inhibition by triacsin C and thiazolidinediones.* J Biol Chem, 2001. **276**(27): p. 24667-73.

46. Van Horn, C.G., et al., *Characterization of recombinant long-chain rat acyl-CoA synthetase isoforms 3 and 6: identification of a novel variant of isoform 6.* Biochemistry, 2005. **44**(5): p. 1635-42.

47. Marcel, Y.L. and G. Suzue, *Kinetic studies on the specificity of long chain acyl coenzyme A synthetase from rat liver microsomes.* J Biol Chem, 1972. **247**(14): p. 4433-6.

48. Stinnett, L., T.M. Lewin, and R.A. Coleman, *Mutagenesis of rat acyl-CoA synthetase 4 indicates amino acids that contribute to fatty acid binding.* Biochim Biophys Acta, 2007. **1771**(1): p. 119-25.

49. Zierz, S. and A.G. Engel, *Different sites of inhibition of carnitine palmitoyltransferase by malonyl-CoA, and by acetyl-CoA and CoA, in human skeletal muscle.* Biochem J, 1987. **245**(1): p. 205-9.

50. Daly, M.E., et al., *Acute fuel selection in response to high-sucrose and high-starch meals in healthy men.* Am J Clin Nutr, 2000. **71**(6): p. 1516-24.

51. Seal, C.J., et al., *Postprandial carbohydrate metabolism in healthy subjects and those with type 2 diabetes fed starches with slow and rapid hydrolysis rates determined in vitro.* Br J Nutr, 2003. **90**(5): p. 853-64.

52. Mukherjee, A., et al., *Studies on human heart citrate synthase.* Adv Myocardiol, 1980. **1**: p. 329-37.

53. Matsumura, T., et al., *O2 uptake in periportal and pericentral regions of liver lobule in perfused liver.* Am J Physiol, 1986. **250**(6 Pt 1): p. G800-5.

54. Kessler, M., J. Hoper, and B.A. Krumme, *Monitoring of tissue perfusion and cellular function.* Anesthesiology, 1976. **45**(2): p. 184-97.

55. Bald, D., et al., *ATP synthesis by F0F1-ATP synthase independent of noncatalytic nucleotide binding sites and insensitive to azide inhibition.* J Biol Chem, 1998. **273**(2): p. 865-70.

56. Soga, N., et al., *Efficient ATP synthesis by thermophilic Bacillus FoF1-ATP synthase.* FEBS J, 2011. **278**(15): p. 2647-54.

57. Richard, P., B. Pitard, and J.L. Rigaud, *ATP synthesis by the F0F1-ATPase from the thermophilic Bacillus PS3 co-reconstituted with bacteriorhodopsin into liposomes. Evidence for stimulation of ATP synthesis by ATP bound to a noncatalytic binding site.* J Biol Chem, 1995. **270**(37): p. 21571-8.

58. Lam, S.C. and M.A. Packham, *Isolation and kinetic studies of nucleoside diphosphokinase from human platelets and effects of cAMP phosphodiesterase inhibitors.* Biochem Pharmacol, 1986. **35**(24): p. 4449-55.

59. Kimura, N. and N. Shimada, *Membrane-associated nucleoside diphosphate kinase from rat liver. Purification, characterization, and comparison with cytosolic enzyme.* J Biol Chem, 1988. **263**(10): p. 4647-53.

60. Fukuchi, T., et al., *Recombinant rat nucleoside diphosphate kinase isoforms (alpha and beta): purification, properties and application to immunological detection of native isoforms in rat tissues.* Biochim Biophys Acta, 1994. **1205**(1): p. 113-22.

61. Tsuboi, K.K. and C.H. Chervenka, *Adenylate kinase of human erythrocyte. Isolation and properties of the predominant inherited form.* J Biol Chem, 1975. **250**(1): p. 132-40.

62. Abu-Elheiga, L., et al., *The subcellular localization of acetyl-CoA carboxylase 2.* Proc Natl Acad Sci U S A, 2000. **97**(4): p. 1444-9.

63. Cheng, D., et al., *Expression, purification, and characterization of human and rat acetyl coenzyme A carboxylase (ACC) isozymes.* Protein Expr Purif, 2007. **51**(1): p. 11-21.

64. Carlisle-Moore, L., et al., *Substrate recognition by the human fatty-acid synthase.* J Biol Chem, 2005. **280**(52): p. 42612-8.

65. Jayakumar, A., et al., *Human fatty acid synthase: properties and molecular cloning.* Proc Natl Acad Sci U S A, 1995. **92**(19): p. 8695-9.

66. Reaven, G.M., et al., *Measurement of plasma glucose, free fatty acid, lactate, and insulin for 24 h in patients with NIDDM.* Diabetes, 1988. **37**(8): p. 1020-4.

67. Monti, L.D., et al., *Myocardial insulin resistance associated with chronic hypertriglyceridemia and increased FFA levels in Type 2 diabetic patients.* Am J Physiol Heart Circ Physiol, 2004. **287**(3): p. H1225-31.

68. Szczepaniak, L.S., et al., *Magnetic resonance spectroscopy to measure hepatic triglyceride content: prevalence of hepatic steatosis in the general population.* Am J Physiol Endocrinol Metab, 2005. **288**(2): p. E462-8.

69. Saggerson, E.D. and A.L. Greenbaum, *The regulation of triglyceride synthesis and fatty acid synthesis in rat epididymal adipose tissue.* Biochem J, 1970. **119**(2): p. 193-219.

70. Zammit, V.A. and C.G. Corstorphine, *Inhibition of acetyl-CoA carboxylase activity in isolated rat adipocytes incubated with glucagon. Interactions with the effects of insulin, adrenaline and adenosine deaminase.* Biochem J, 1982. **208**(3): p. 783-8.

71. Halestrap, A.P. and R.M. Denton, *Hormonal regulation of adipose-tissue acetyl-Coenzyme A carboxylase by changes in the polymeric state of the enzyme. The role of long-chain fatty acyl-Coenzyme A thioesters and citrate.* Biochem J, 1974. **142**(2): p. 365-77.

72. Vancura, A. and D. Haldar, *Purification and characterization of glycerophosphate acyltransferase from rat liver mitochondria.* J Biol Chem, 1994. **269**(44): p. 27209-15.

73. Farese, R.V., et al., *Insulin-induced activation of glycerol-3-phosphate acyltransferase by a chiro-inositol-containing insulin mediator is defective in adipocytes of insulin-resistant, type II diabetic, Goto-Kakizaki rats.* Proc Natl Acad Sci U S A, 1994. **91**(23): p. 11040-4.

74. Kaplan, A. and M.H. Teng, *Interaction of beef liver lipase with mixed micelles of tripalmitin and Triton X-100.* J Lipid Res, 1971. **12**(3): p. 324-30.

75. Chakraborty, K. and R.P. Raj, *An extra-cellular alkaline metallolipase from Bacillus licheniformis MTCC 6824: Purification and biochemical characterization.* Food Chemistry, 2008. **109**(4): p. 727-736.

76. Westergaard, N., P. Madsen, and K. Lundgren, *Characterization of glycerol uptake and glycerol kinase activity in rat hepatocytes cultured under different hormonal conditions.* Biochim Biophys Acta, 1998. **1402**(3): p. 261-8.

77. Mashek, D.G., *Hepatic fatty acid trafficking: multiple forks in the road.* Adv Nutr, 2013. **4**(6): p. 697-710.

78. Falcon, A., et al., *FATP2 is a hepatic fatty acid transporter and peroxisomal very long-chain acyl-CoA synthetase.* American Journal of Physiology-Endocrinology and Metabolism, 2010. **299**(3): p. E384-E393.

79. Doege, H., et al., *Targeted deletion of FATP5 reveals multiple functions in liver metabolism: Alterations in hepatic lipid Homeostasis.* Gastroenterology, 2006. **130**(4): p. 1245-1258.

80. Koonen, D.P.Y., et al., *Increased hepatic CD36 expression contributes to dyslipidemia associated with diet-induced obesity.* Diabetes, 2007. **56**(12): p. 2863-2871.

81. Greco, D., et al., *Gene expression in human NAFLD.* American Journal of Physiology-Gastrointestinal and Liver Physiology, 2008. **294**(5): p. G1281-G1287.

82. Mitsuyoshi, H., et al., *Analysis of hepatic genes involved in the metabolism of fatty acids and iron in nonalcoholic fatty liver disease.* Hepatology Research, 2009. **39**(4): p. 366-373.

83. Newberry, E.P., et al., *Decreased hepatic triglyceride accumulation and altered fatty acid uptake in mice with deletion of the liver fatty acid-binding protein gene.* Journal of Biological Chemistry, 2003. **278**(51): p. 51664-51672.

84. Rajaraman, G., et al., *Membrane binding proteins are the major determinants for the hepatocellular transmembrane flux of long-chain fatty acids bound to albumin.* Pharmaceutical Research, 2005. **22**(11): p. 1793-1804.

85. Stump, D.D., et al., *Characteristics of Oleate Binding to Liver Plasma-Membranes and Its Uptake by Isolated Hepatocytes.* Journal of Hepatology, 1992. **16**(3): p. 304-315.

86. Buque, X., et al., *High insulin levels are required for FAT/CD36 plasma membrane translocation and enhanced fatty acid uptake in obese Zucker rat hepatocytes.* Am J Physiol Endocrinol Metab, 2012. **303**(4): p. E504-14.

87. Stahl, A., et al., *Insulin causes fatty acid transport protein translocation and enhanced fatty acid uptake in adipocytes.* Dev Cell, 2002. **2**(4): p. 477-88.

88. Ge, F., et al., *Insulin- and leptin-regulated fatty acid uptake plays a key causal role in hepatic steatosis in mice with intact leptin signaling but not in ob/ob or db/db mice.* Am J Physiol Gastrointest Liver Physiol, 2010. **299**(4): p. G855-66.

89. Durrington, P.N., et al., *Effects of Insulin and Glucose on Very Low-Density Lipoprotein Triglyceride Secretion by Cultured Rat Hepatocytes.* Journal of Clinical Investigation, 1982. **70**(1): p. 63-73.

90. Nelson, R.H., et al., *Triglyceride uptake and lipoprotein lipase-generated fatty acid spillover in the splanchnic bed of dogs.* Diabetes, 2007. **56**(7): p. 1850-5.

91. Quarfordt, S.H., et al., *Differing uptake of emulsion triglyceride by the fed and fasted rat liver.* J Clin Invest, 1982. **69**(5): p. 1092-8.

92. Bergman, E.N., et al., *Quantitative studies of the metabolism of chylomicron triglycerides and cholesterol by liver and extrahepatic tissues of sheep and dogs.* J Clin Invest, 1971. **50**(9): p. 1831-9.

93. Jungermann, K. and N. Katz, *Functional specialization of different hepatocyte populations.* Physiol Rev, 1989. **69**(3): p. 708-64.

94. Jungermann, K. and T. Kietzmann, *Role of oxygen in the zonation of carbohydrate metabolism and gene expression in liver.* Kidney Int, 1997. **51**(2): p. 402-12.

95. Nauck, M., et al., *Modulation of the glucagon-dependent induction of phosphoenolpyruvate carboxykinase and tyrosine aminotransferase by arterial and venous oxygen concentrations in hepatocyte cultures.* Eur J Biochem, 1981. **119**(3): p. 657-61.

96. Wolfle, D. and K. Jungermann, *Long-term effects of physiological oxygen concentrations on glycolysis and gluconeogenesis in hepatocyte cultures.* Eur J Biochem, 1985. **151**(2): p. 299-303.

97. Jackson, R.C., H.P. Morris, and G. Weber, *Partial purification, properties and regulation of inosine 5'phosphate dehydrogenase in normal and malignant rat tissues.* Biochem J, 1977. **166**(1): p. 1-10.

98. Donnelly, K.L., et al., *Sources of fatty acids stored in liver and secreted via lipoproteins in patients with nonalcoholic fatty liver disease.* J Clin Invest, 2005. **115**(5): p. 1343-51.

99. Guzman, M. and J. Castro, *Zonal Heterogeneity of the Effects of Chronic Ethanol Feeding on Hepatic Fatty-Acid Metabolism.* Hepatology, 1990. **12**(5): p. 1098-1105.

100. Katz, N., et al., *Heterogeneous reciprocal localization of fructose-1,6-bisphosphatase and of glucokinase in microdissected periportal and perivenous rat liver tissue.* FEBS Lett, 1977. **83**(2): p. 272-6.

101. Lawrence, G.M., et al., *The compartmentation of glycolytic and gluconeogenic enzymes in rat kidney and liver and its significance to renal and hepatic metabolism.* Histochem J, 1986. **18**(1): p. 45-53.

102. Lawrence, G.M., I.P. Trayer, and D.G. Walker, *Histochemical and immunohistochemical localization of hexokinase isoenzymes in normal rat liver.* Histochem J, 1984. **16**(10): p. 1099-111.

103. Fischer, W., M. Ick, and N.R. Katz, *Reciprocal distribution of hexokinase and glucokinase in the periportal and perivenous zone of the rat liver acinus.* Hoppe Seylers Z Physiol Chem, 1982. **363**(4): p. 375-80.

104. Teutsch, H.F. and O.H. Lowry, *Sex specific regional differences in hepatic glucokinase activity.* Biochem Biophys Res Commun, 1982. **106**(2): p. 533-8.

105. Trus, M., et al., *Hexokinase and glucokinase distribution in the liver lobule.* J Histochem Cytochem, 1980. **28**(6): p. 579-81.

106. Eilers, F., H. Bartels, and K. Jungermann, *Zonal expression of the glucokinase gene in rat liver. Dynamics during the daily feeding rhythm and starvation-refeeding cycle demonstrated by in situ hybridization.* Histochemistry, 1993. **99**(2): p. 133-40.

107. Moorman, A.F., et al., *Pericentral expression pattern of glucokinase mRNA in the rat liver lobulus.* FEBS Lett, 1991. **287**(1-2): p. 47-52.

108. Kirchner, G., et al., *Zonation of glucokinase in rat liver changes during postnatal development.* FEBS Lett, 1993. **328**(1-2): p. 119-24.

109. Wals, P.A., M. Palacin, and J. Katz, *The zonation of liver and the distribution of fructose 2,6-bisphosphate in rat liver.* J Biol Chem, 1988. **263**(10): p. 4876-81.

110. Teutsch, H.F., *Quantitative determination of G6Pase activity in histochemically defined zones of the liver acinus.* Histochemistry, 1978. **58**(4): p. 281-8.

111. Katz, N., et al., *Heterogeneous distribution of glucose-6-phosphatase in microdissected periportal and perivenous rat liver tissue.* FEBS Lett, 1977. **76**(2): p. 226-30.

112. Royal-Free-Liver&Digestive-Health, *Unpublished Data.* 2013.

113. Giffin, B.F., et al., *Hepatic lobular patterns of phosphoenolpyruvate carboxykinase, glycogen synthase, and glycogen phosphorylase in fasted and fed rats.* J Histochem Cytochem, 1993. **41**(12): p. 1849-62.

114. Frederiks, W.M., F. Marx, and C.J. Van Noorden, *Quantitative histochemical assessment of the heterogeneity of glycogen phosphorylase activity in liver parenchyma of fasted rats using the semipermeable membrane technique and the PAS reaction.* Histochem J, 1987. **19**(3): p. 150-6.

115. Frederiks, W.M., F. Marx, and C.J. van Noorden, *Homogeneous distribution of phosphofructokinase in the rat liver acinus: a quantitative histochemical study.* Hepatology, 1991. **14**(4 Pt 1): p. 634-9.

116. Lamas, E., A. Kahn, and A. Guillouzo, *Detection of mRNAs present at low concentrations in rat liver by in situ hybridization: application to the study of metabolic regulation and azo dye hepatocarcinogenesis.* J Histochem Cytochem, 1987. **35**(5): p. 559-63.

117. Eilers, F., S. Modaressi, and K. Jungermann, *Predominant periportal expression of the fructose 1,6-bisphosphatase gene in rat liver: dynamics during the daily feeding rhythm and starvation-refeeding cycle.* Histochem Cell Biol, 1995. **103**(4): p. 293-300.

118. Bartels, H., S. Freimann, and K. Jungermann, *Predominant periportal expression of the phosphoenolpyruvate carboxykinase gene in liver of fed and fasted mice, hamsters and rats studied by in situ hybridization.* Histochemistry, 1993. **99**(4): p. 303-9.

119. Bartels, H., H. Herbort, and K. Jungermann, *Predominant periportal expression of the phosphoenolpyruvate carboxykinase and tyrosine aminotransferase genes in rat liver. Dynamics during the daily feeding rhythm and starvation-refeeding cycle demonstrated by in situ hybridization.* Histochemistry, 1990. **94**(6): p. 637-44.

120. Bartels, H., H. Linnemann, and K. Jungermann, *Predominant localization of phosphoenolpyruvate carboxykinase mRNA in the periportal zone of rat liver parenchyma demonstrated by in situ hybridization.* FEBS Lett, 1989. **248**(1-2): p. 188-94.

121. Jones, C.G. and M.A. Titheradge, *Measurement of metabolic fluxes through pyruvate kinase, phosphoenolpyruvate carboxykinase, pyruvate dehydrogenase, and pyruvate carboxylate in hepatocytes of different acinar origin.* Arch Biochem Biophys, 1996. **326**(2): p. 202-6.

122. Loud, A.V., *A quantitative stereological description of the ultrastructure of normal rat liver parenchymal cells.* J Cell Biol, 1968. **37**(1): p. 27-46.

123. Wimmer, M. and D. Pette, *Microphotometric studies on intraacinar enzyme distribution in rat liver.* Histochemistry, 1979. **64**(1): p. 23-33.

124. Novikoff, A.B., *Cell Heterogeneity within the Hepatic Lobule of the Rat (Staining Reactions).* Journal of Histochemistry & Cytochemistry, 1959. **7**(4): p. 240-244.

125. Katz, N.R., W. Fischer, and S. Giffhorn, *Distribution of enzymes of fatty acid and ketone body metabolism in periportal and perivenous rat-liver tissue.* Eur J Biochem, 1983. **135**(1): p. 103-7.

126. Katz, N., J. Thiele, and S. Giffhorn-Katz, *Zonal distribution of fatty acid synthase in liver parenchyma of male and female rats.* Eur J Biochem, 1989. **180**(1): p. 185-9.

127. Katz, N.R., W. Fischer, and M. Ick, *Heterogeneous distribution of ATP citrate lyase in rat-liver parenchyma. Microradiochemical determination in microdissected periportal and perivenous liver tissue.* Eur J Biochem, 1983. **130**(2): p. 297-301.

128. Morrison, G.R., et al., *Quantitative analysis of regenerating and degenerating areas within the lobule of the carbon tetrachloride-injured liver.* Arch Biochem Biophys, 1965. **111**(2): p. 448-60.

129. Guzman, M. and J. Castro, *Zonation of fatty acid metabolism in rat liver.* Biochem J, 1989. **264**(1): p. 107-13.

130. Bass, N.M., et al., *Acinar heterogeneity of fatty acid binding protein expression in the livers of male, female and clofibrate-treated rats.* Hepatology, 1989. **9**(1): p. 12-21.

131. Suzuki, T. and T. Ono, *Immunohistochemical studies on the distribution and frequency of fatty-acid-binding protein positive cells in human fetal, newborn and adult liver tissues.* J Pathol, 1987. **153**(4): p. 385-94.

132. Krones, A., T. Kietzmann, and K. Jungermann, *Perivenous localization of insulin receptor protein in rat liver, and regulation of its expression by glucose and oxygen in hepatocyte cultures.* Biochem J, 2000. **348 Pt 2**: p. 433-8.

133. Krones, A., T. Kietzmann, and K. Jungermann, *Periportal localization of glucagon receptor mRNA in rat liver and regulation of its expression by glucose and oxygen in hepatocyte cultures.* FEBS Lett, 1998. **421**(2): p. 136-40.

134. Bass, N.M., *Fatty acid-binding protein expression in the liver: its regulation and relationship to the zonation of fatty acid metabolism.* Mol Cell Biochem, 1990. **98**(1-2): p. 167-76.
