# Supplementary material for: A Computational Model of Hepatic Energy Metabolism: Understanding Zonated Damage and Steatosis in NAFLD
Source: PLoS Comput Biol. 2016 Sep 15;12(9):e1005105. doi: 10.1371/journal.pcbi.1005105 (PMC5025084; doi:10.1371/journal.pcbi.1005105)
Supplement: S2 Text — (DOCX) [file pcbi.1005105.s002.docx]

# S2 Text. Comparisons with Experimental Data

In the ‘*Validation of Liver Metabolism in Healthy Patients*’ section, the model simulations are compared with experimental data for metabolically healthy individuals. In the ‘*Concentrations of Intermediates and of the Energy Molecules*’ subsection the average concentrations of the various hepatic metabolism intermediates are compared with experimental data. In the ‘*Glycogen Synthesis After a Mixed Meal*’ subsection, the rate of postprandial glycogen synthesis is compared with experimental data. In the ‘*Metabolic Rates*’ subsection the rate at which various metabolic processes occur under conditions leading to hepatic glucose production and to hepatic glucose clearance. In the ‘*Plasma Concentrations Throughout the Day*’ subsection, the effects of a daily intake/use cycle on plasma concentrations of glucose, lactate, FFAs, triglycerides and insulin are compared with experimental data. In the ‘*Validation of Zonation*’ subsection, the simulated rates of various processes across the sinusoid are compared with experimental data. Very little data regarding the concentrations of molecules in different regions of the sinusoid have been published. Instead the variation in rates of different glucose and lipid metabolism processes are compared with experimental data.

In the ‘*Validation of the Simulated Data in Insulin Resistant Patients*’ section, the effects of insulin resistance on plasma variables are validated against experimental data. A more detailed study of the effects of insulin resistance on hepatic metabolism is performed in the main text.

In the ‘*Validation of the relative contributions of lipids and carbohydrates to oxidative phosphorylation*’ section, the simulated post-prandial changes in the relative rates of glucose oxidation and β-oxidation are compared with experimental data when simulating metabolically normal and insulin resistant individuals.

## Validation of Liver Metabolism in Healthy Patients

### Concentrations of Intermediates and of the Energy Molecules

| Protein | Average Value (Low-high over intake cycle)  – Model (mM) | Average Value (Confidence Interval)  – Experimental (mM) |
| --- | --- | --- |
| Inorganic Phosphate | 3.96 (3971-4011) | 4.07 (3.55-4.07) [[1](#_ENREF_1)] |
| ATP | 2.82 (2.64-2.96) | 2.78 (2.71-2.85) [[1](#_ENREF_1)] |
| ADP | .832 (.765-.926) | .885 (.794-.976) [[2](#_ENREF_2)] |
| AMP | .269 (.223-.337) | .237 (.200-.272) [[1](#_ENREF_1)] |
| UTP | .324 (.205-.365) | .285 (.255-.315) [[2](#_ENREF_2)] |
| UDP | .067 (.027-.188) | .108 (.096-.120) [[2](#_ENREF_2)] |
| GTP | .274 (.271-.275) | .277 (.266-.288) [[2](#_ENREF_2)] |
| GDP | .101 (.100-.104) | .098 (.091-.105) [[2](#_ENREF_2), [3](#_ENREF_3)] |
| Hepatic Glucose | 8.24 (7.28-10.0) | 9.86-10.20 [[3](#_ENREF_3), [4](#_ENREF_4)],[[5](#_ENREF_5)] |
| G6P | .085 (.031-.126) | 0.071 ± 0.004mM – starved rats,  0.133 ± 0.010mM – well fed rats [[1](#_ENREF_1)] |
| GADP | .236 (.195-.312) | 0.2-1.0 [[4](#_ENREF_4)] |
| Acetyl-CoA | .046 (.35-.58) | .039 [[4](#_ENREF_4)] |
| Glycogen | ~200mM  (0 - 500mM)  In glucose units | Since glycogen is used for glucose storage, the liver glycogen concentration is strongly dependent on previous feeding conditions. See ‘*Glycogen Synthesis After a Mixed Meal*’ section for comparison with Taylor *et al.* [[6](#_ENREF_6)]. |
| Hepatic Triglyceride | Average: 38.1mM (37.6-38.4mM)   - 2.31% (2.28-2.33%)   Periportal: 33.6mM (33.1-34.0mM)   - 2.05% (2.01-2.07%)   Pericentral: 45.5mM (45.7-45.0mM)   - 2.75% (2.72-2.76%) | Slightly lower than the average but within the 90^th^ percentile of liver triglyceride concentrations measured in the population by Szczepaniak et al. consistent with simulating a healthy diet [[7](#_ENREF_7)]. Change across the sinusoid consistent with the values of 59.2±5.8 and 65.7±4.0 nmol/mg (protein) in isolated periportal and pericentral cells respectively measured by Guzman *et al.* [[8](#_ENREF_8)]. |

Table 1. The concentration of various hepatic metabolism intermediates and of the mono-, di-, and tri-phosphate molecules included in the model when simulating a moderate intake diet of 70% carbohydrate: 30% FFA, averaged over each 4 hour input cycle.

In Table 1, the average concentrations of the various hepatic metabolism intermediates and the mono-, di-, and tri-phosphate molecules (when simulating the 4 hour moderate intake cycle discussed in S1 Text – ‘*Input*’ section) are compared with experimentally measured values. It is important that, when simulating normal physiological conditions, the outputted concentrations remain in a realistic range.

The simulated average values for hepatic glucose and triglycerides along with glucose metabolism intermediates G6P, G3P and acetyl-CoA are within the experimentally measured ranges. Similarly, the simulated average concentrations for inorganic phosphate, ATP, ADP, AMP, GTP and GDP are all within the experimentally measured confidence intervals. This shows that, when an input approximating a daily meal cycle is provided, the model gives realistic values for the average values of key intermediates of glucose and lipid metabolism in hepatocytes, along with the adenosine and guanine phosphate molecules.

Only the simulated UTP and UDP concentrations are outside of the experimentally measured confidence intervals. The simulated average UTP concentration is 13% higher (+39µM) than the value measured experimentally, whilst the UDP concentration is 39µM lower, suggesting that the activity of NDKU in converting UDP to UTP is overestimated in the model. Therefore, a potential solution to return these concentrations to the experimentally measured range would be to reduce the rate constant for NDKU in the model. However, due to the strong dependence of the UTP concentration on feeding state in the model, if the rate constant for NDKU is reduced by more than around 20% from its value in the model, the simulated UTP concentration in periportal cells falls to near zero during periods of high glycogenesis. This disrupts the storage of glucose as glycogen in these cells after feeding. On the other hand, since the Michaelis-Menten constant for UTP in glycogen synthase is low (48µM) relative to the average UTP concentration (285µM experimental/324µM simulated), the small increase in simulated average UTP concentration has little effect on overall metabolism. It is possible that NDK shows zonated expression *in vivo* with higher activity in the periportal zone to match the increased glycogenesis in this region of the sinusoid.

In addition to the data presented, the relative concentrations of the various molecules are largely consistent the data presented by Faupel *et al.* [[9](#_ENREF_9)] in the vast majority of cases. However, since the experimental data was normalised against DNA weight rather than total protein or cell volume it was not possible to compare absolute values and this data is not presented. Furthermore, the relative concentrations of the various metabolic intermediates are consistent with the data published by Saggerson et al. [[10](#_ENREF_10)]. However, since this data is for adipose tissue, the comparisons are also not presented.

### Glycogen Synthesis After a Mixed Meal


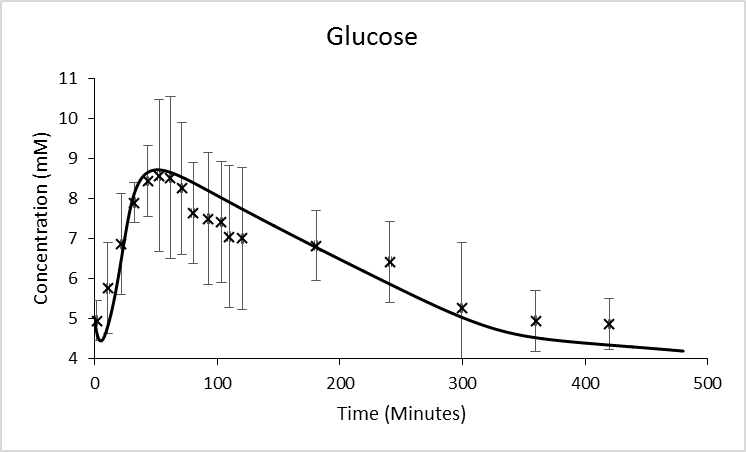

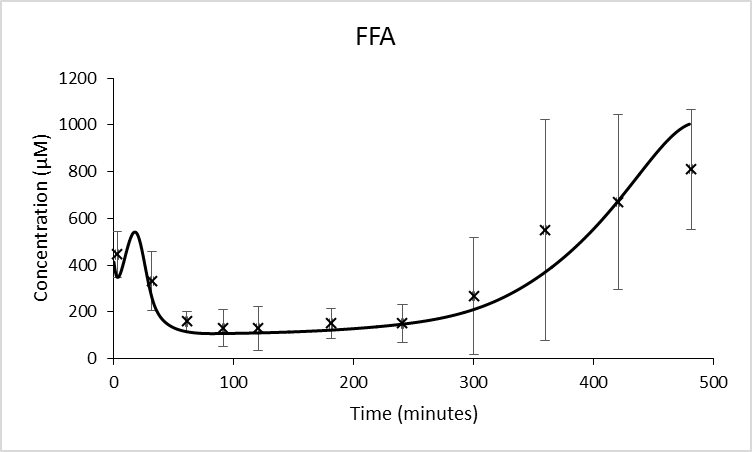

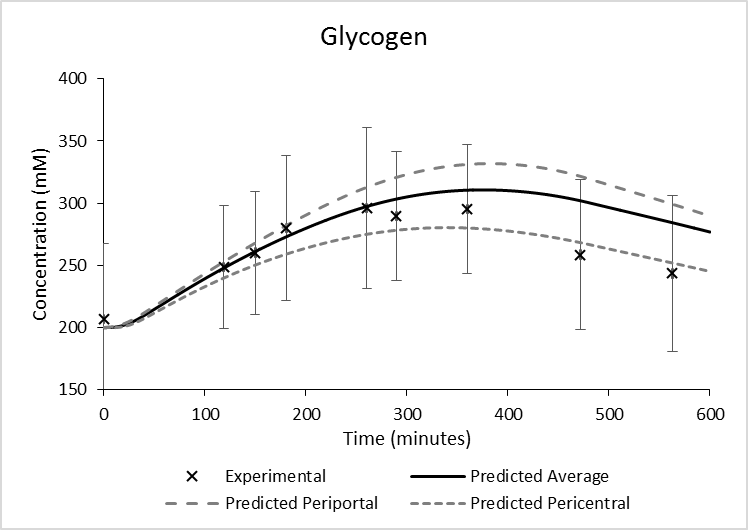


a)

b)

c)

Fig 1. The simulated concentrations of (a) glucose, (b) FFA and (c) glycogen concentrations after input of a mixed meal in which 62% of the energy comes from carbohydrate and 38% from lipid compared with experimental data from Taylor et al. [[6](#_ENREF_6)]. Experimental data approximated from the graphs provided using image J. The error bars on the experimental data are the SD based on the SEM provided.

In addition to providing realistic values for average concentrations, the model outputs must also match experimental data for the changes in plasma and hepatic concentrations under conditions of known intake. The liver is the major tissue responsible for the storage of glucose as glycogen. Therefore, the model must give reasonable values for the change in glycogen storage, along with plasma glucose and FFA concentrations under conditions in which known quantities of glucose and fat are consumed. The simulated data were compared with experimental data published by Taylor *et al.* [[6](#_ENREF_6)] for glucose, FFA and glycogen concentrations after intake of a mixed meal in which 62% of the energy comes from carbohydrate and 38% from lipid (neglecting the protein content of the meal provided experimentally) [[6](#_ENREF_6)] (Fig 1). Since the exact rates at which glucose and FFAs enter the blood stream are unknown, inputs with an initial spike followed by a slow linear decrease were used. The average differences between the simulated and measured glucose and FFA curves over the first 5 hours (excluding t=0) are 0.3 standard deviations (420µM) and 0.51 standard deviations (34µM) respectively. After 6 hours, the simulated glucose concentration falls to a slightly lower baseline value than the experimental data, whilst the simulated increase in FFA concentration is delayed relative to the experimental data. However, in both cases the simulated data remain within one standard deviation of the experimental data, and the shape of the simulated curves remains consistent with the experimental data.

The simulated average glycogen concentration remains on average 0.1 standard deviations from the experimentally measured mean concentrations for the first 6 hours after the mixed meal. This shows that the model is able to accurately represent the storage of glucose as glycogen after intake of a mixed meal. After 6 hours, the fall in glycogen concentration is larger in the experimental data than in the simulated data, but the difference between the two curves remains well within one standard deviation. Over a period of longer than 6 hours, numerous factors may explain the more rapid fall in the experimental curve compared with the model simulations. For example, the model does not account for any variation in the activity of the patient and energy is consumed at a roughly constant rate (dependent only on plasma concentrations of hormones, FFA and glucose).

The simulated compartment 1 (proximal periportal) and compartment 8 (distal perivenous/pericentral) concentrations are also plotted to show the difference across the sinusoid in glycogen storage. Both *in vivo* [[11](#_ENREF_11)], and in the model simulations a high rate of glycogen synthesis is seen in periportal cells post-prandially.

### Metabolic Rates

#### During Hepatic Glucose Production

| Process | Simulated % of Glycogen Breakdown (mM/s) | Experimental % of Glycogen Breakdown (nmol.mg^-1^.min^-1^) [[12](#_ENREF_12)] |
| --- | --- | --- |
| Glycogen Breakdown | 100% (5.63 [Glucose]) | 100% (8.67 [Glucose]) |
| Glucose Production | 54.7% (3.08 [Glucose]) | 54.4% (4.72 [Glucose]) |
| Glycolysis | 45.3% (2.55 [Glucose]) | 45.7% (3.96 [Glucose]) |
| Lactate Release | 31.3% (1.76 [Glucose]*) | 35.6% (3.09 [Glucose]*) |
| Pyruvate Oxidation | 24.3% (1.37 [Glucose]*) | ~20.1% (8.72 [Ox]) |
| Lipogenesis | 3.3% (0.19 [Glucose]*) | ~0 |
| Mitochondrial Oxidation | 115 [ATP] | 18.01 [ATP] |
| ATP Consumption | 199 [ATP] | 31.63 [ATP] |

Table 2. Hepatic metabolic rates compared with experimental data from Ainscow and Brand under conditions of glycogen breakdown [[12](#_ENREF_12)]. To simulate conditions of constant glycogen breakdown, the plasma fatty acid, glucose, lactate, insulin and glucagon concentrations were set to fixed values of 0.4mM, 5mM, 0.45mM, 0mM and 31.5pM. Rates were taken after 2000s, once they had reached equilibrium. * equivalents of glucose.

Table 2 compares the simulated hepatic metabolic rates with those presented by Ainscow and Brand for isolated hepatocytes under conditions of glycogen breakdown [[12](#_ENREF_12)]. To simulate conditions providing glycogen breakdown consistent with the experimental study, the plasma fatty acid, glucose, lactate, insulin and glucagon concentrations were set to fixed values of 0.4mM, 5mM, 0.45mM, 0mM and 31.5pM. Rates were taken after 2000s, once they had reached equilibrium.

Although comparison of the absolute values is not possible due to differences in the units, the relative rates of all of the processes provide a reasonable approximation to the experimental data. In both the simulated and experimental data, approximately 55% of G6P derived from glycogen breakdown was converted to glucose, whilst 45% underwent glycolysis. 31.3% of the G6P was released as lactate in the model compared with 35.6% experimentally. 24.3% underwent oxidation to acetyl-CoA compared with 20.1% experimentally. Therefore, a slightly higher relative rate of pyruvate oxidation occurred in the model than is seen experimentally, but the values remain well within a range which could be accounted for by small differences in plasma and hepatic metabolite concentrations. Only a very low rate of lipogenesis occurred when simulating the presence of glucagon but not insulin, consistent with the experimental study. The ratio of ATP consumption to mitochondrial oxidation (1.73:1) is within 2% of the experimentally measured ratio (1.75:1). However absolute comparison is not possible since the experimental data is provided in units of moles per milligram of tissue rather than moles per litre. These results demonstrate that the model provides appropriate relative metabolic rates under conditions of glycogen breakdown.

#### During Glucose Clearance

| Hepatic Glucose Removal | Basal | Moderate Insulin (173pM) | High Insulin  (700pM) |
| --- | --- | --- | --- |
| Simulated:  μmol/L/s (fold change from basal) | 11.3 (1.00) | -7.1 (-0.62) | -11.4 (-1.01) |
| Experimental Removal:  mg/kg/min (fold change from basal) | 1.3±0.4 (1.00) | -0.2±0.2 (-0.15) | -0.9±.7 (-0.69) |
| Experimental Glycogen Synthesis: mg/kg/min (fold change from basal) | 0.9±0.4 (1.00) | -1±0.4 (-1.11) | -1.5±0.5 (-1.67) |

| Gluconeogenesis | Basal | Moderate Insulin (173pM) | High Insulin  (700pM) |
| --- | --- | --- | --- |
| Simulated: Change in gluconeogenesis (μmol/L/s) | 0 | -1.2 | -4.7 |
| Simulated: Change in net gluconeogenesis (μmol/L/s) | 0 | -0.56 | -3.4 |
| Experimental: Change in Flux to G6P (mg/kg/min) | 0±0.4 | -0.1±0.7 | -0.5±0.7 |

Tables 3 and 4. Comparison of the simulated effects of insulin stimulation on hepatic glucose production and storage with experimentally measured values for canine livers [[13](#_ENREF_13)]. Simulated using a fixed plasma glucagon concentrations of 93.75pM and fixed plasma glucose, lactate, insulin and FFA concentrations at the values measured experimentally.

| Non-Oxidative Glucose Metabolism | Basal | Moderate Insulin (340pM) | Very High Insulin  (>1nm) |
| --- | --- | --- | --- |
| Simulated (fold change from basal) | 1.0 | 6.4 | 8.2 |
| Experimental (fold change from basal) | 1.0±0.3 | 5.9±1.0 | 17±3 |

| Oxidative Glucose Metabolism | Basal | Moderate Insulin (340pM) | Very High Insulin  (>1nm) |
| --- | --- | --- | --- |
| Simulated (fold change from basal) | 1.0 | 1.4 | 2.9 |
| Experimental (fold change from basal) | 1.0±0.1 | 2.3±0.2 | 2.7±0.3 |

| β-Oxidation | Basal | Moderate Insulin (340pM) | Very High Insulin  (>1nm) |
| --- | --- | --- | --- |
| Simulated (fold change from basal) | 1.0 | 0.67 | -0.56 |
| Experimental (fold change from basal) | 1.0±0.14 | 0.21±0.22 | -0.53±0.24 |

Tables 5-7. Comparison of the simulated effects of insulin stimulation on hepatic metabolic rates and experimentally measured values for muscle [[14](#_ENREF_14)]. Simulated using fixed plasma glucose, lactate and glucagon concentrations of 5mM, 0.9mM, 0mM and fixed plasma insulin and FFA concentrations at the values measured experimentally. For the very high insulin infusion experimentally, a concentration of greater than 4nM was measured. In the model, a concentration this high caused non-continuous jumps even for small integration step sizes. Instead, a fixed insulin concentration of 1nM was simulated. However, further increases in insulin concentration from 1nM have little effect on rates when simulating conditions of fixed plasma concentration in the model because substrate and allosteric regulation become rate limiting.

A similar study under conditions of glycogen synthesis was not available. However, Tables 3 and 4 compare the simulated rates of glucose uptake and gluconeogenesis at three plasma insulin concentrations with experimentally measured values for canine livers from Edgerton *et al.* [[13](#_ENREF_13)], whilst Tables 5-7 compare the effects of insulin on the rates of hepatic glucose removal, non-oxidative glucose metabolism, oxidative glucose metabolism and β-oxidation with experimental data from Mandarino *et al.* for the processes in muscle [[14](#_ENREF_14)] (presented normalised to basal).

Since the experimental results in Tables 3 and 4 are expressed per kg of liver, absolute comparisons of the rates are not possible. Furthermore, unlike when considering glycogen breakdown, the percentage of molecules moving through each process relative to an initial source could not be calculated making comparison more difficult. However, the relative effects of basal and high insulin concentrations on glucose removal and gluconeogenesis are equivalent in the simulated and experimental data. High insulin concentrations cause a rate of glucose output approximately equal to the rate of clearance at basal insulin in both sets (averaging the two experimental measurements). Additionally the relative effects of moderate to high insulin perfusion on the system are similar in the simulated and experimental data. The effect of the moderate insulin concentration on hepatic glucose removal was approximately 80% of the change due to the high insulin concentration in the simulated and experimental data sets for both glucose removal and glycogen synthesis. At the moderate insulin concentration, suppression of gluconeogenesis was approximately 20% of the change for the high insulin concentration in both the simulated and experimental data.

The fold increase in the rate of non-oxidative glucose metabolism is well within one standard deviation of the experimental data at the moderate insulin concentration (Table 5). The very high insulin concentration had a smaller effect in the simulated data than is seen experimentally. However, one possible explanation for this may be that muscle cells rather than hepatocytes were studied experimentally and muscle cells are known to undergo extensive anaerobic glycolysis during exercise. Furthermore muscle cells do not undergo gluconeogenesis due to a lack of glucose-6-phosphatase [[15](#_ENREF_15)]. As a result, they would be expected to have a maximum non-oxidative metabolic capacity higher than liver cells.

The simulated fold change in oxidative glucose metabolism (Table 6) and β-oxidation (Table 7) are smaller than the experimental data for moderate insulin concentrations. Liver has higher baseline activity of both β-oxidation and overall oxidative metabolism than muscle such that the relative increase when simulated by insulin is initially smaller. At very high insulin concentrations, the drop in FFA concentration, rather than the direct effects of insulin itself, forced hepatocytes to switch to much higher glucose oxidation and lower β-oxidation consistent with the experimental data.

### Plasma Concentrations Throughout the Day

Fig 2 compares the simulated plasma glucose, FFA, insulin and triglyceride concentrations (red) plotted with experimental data measured by Daly *et al.* (blue) throughout a daily meal cycle [[17](#_ENREF_17)]. The model was provided with a spiked input every four hours and twenty minutes roughly corresponding to the food intake provided experimentally (as discussed in S1 Text). However, in the experimental study, the meals were not equally spaced and differed in size. In the experimental study, the three meals (breakfast, lunch and dinner) contained an average of 78.1g of carbohydrate and 22.2g of fat. In terms of the energy provided from these two sources this was a ratio of 1.648 to 1. When running the simulations, the size of the glucose and FFA inputs were set to match these values. Each intake cycle was set to provide 86.74mmoles of glucose per litre of blood into the body compartment. Presuming a blood volume of 5L, this corresponds to 78.1g. Since fatty acids have a range of molar masses, the FFA input to the model in moles could not be calculated from the grams of fat per meal. Instead the relative contributions of glucose and fatty acids to energy were used to set the inputs. In the model, each glucose molecules that undergoes glycolysis followed by oxidation provides a net total of 34 ATP. Each palmitate molecules that is oxidised provides a net of 114 ATP. Therefore to attain the ratio of 1.648:1 provided in the study, the FFA input was set to provide 15.75mmoles of fat per litre of blood per intake cycle to the body compartment. Using the molar mass of palmitate and assuming 5L of total blood this would correspond to 20.25g of fat.

Since the spiked periodic input provided is only a crude approximation to the rate of intake of glucose and fatty acids (in the experimental study the meals were neither equal in size nor equally spaced), it was considered inappropriate to base the comparison of the simulated and experimental curves on a simple calculation of the squared difference of the curves. When this analysis was performed, although the difference in food intake times meant that whilst the differences between the simulated (χ) and experimental ($x$) curves remained low for the majority of time points, high errors were seen around meal times (e.g. 13:00-14:00), particularly for glucose and insulin due to differences in the exact timing of intake. As a result the summed error over variance for glucose and lactate were high at 1.30 and 1.08 respectively (calculated as $\frac{\sum_{i=1}^{i=n} \frac{\left| x\left( i \right)-\chi(i) \right|}{\sigma(i)}}{n}$, where $i=1:n$ are times at which experimental data points were available). The summed error over variance for triglyceride was also large at 1.59.

Instead, to give a more representative quantitative comparison of simulated and experimental data, Table 8 compares the peak, trough and average concentrations for each variable along with the offset in the peak time from the glucose peak. Values for lactate and glycerol are additionally included in this table. These measures are less dependent on the differences in meal time and size. Since data points were taken at 30 minute intervals in the data, there is a 30 minute uncertainty in the time at which peaks occurred experimentally.

The simulated glucose, insulin and FFAs concentrations replicate the key features of experimental data. In both the simulated and experimentally measured data, the glucose and insulin concentrations show sharp post-prandial peaks followed by broad troughs between meals. The FFA concentration gradually rises between meals before falling rapidly postprandially in both data sets.

The simulated peak concentrations are equal in magnitude because equal meal sizes were simulated, whilst the experimental peaks increase throughout the day due to increases in the size of the meal. As a result, the final peak matched the experimental data well, but the simulated first and second post-prandial glucose peaks were 1.09 and 0.74 standard deviations higher than measured respectively. The simulated peak concentrations were 0.79 standard deviations higher than the average peak in the experimental data. However, since readings were taken at 30 minute intervals in the experimental study, and the peaks in glucose concentration are very sharp, the true peak concentration is likely to be higher than the experimentally measured peak.

The simulated average peak insulin concentration closely matches the experimentally measured value (0.27 standard deviations). However, the simulated plasma concentration falls to effectively zero between meals whilst the experimentally measured value only falls to at 29±24pM. This is because no insulin release occurs between meals in the model, where some release would be expected *in vivo*. A concentration this small is unlikely to have a large effect on metabolism and will be dwarfed by the effects of the raised glucagon concentration.

The simulated FFA concentration curve matches the experimental data well. Sharp pre-prandial peaks in concentration occurred in the simulated data. This would not be seen experimentally because the experimental data provides an average over several patients with readings taken at half hour intervals. Therefore comparison of this aspect of the simulated data with the experimental data is not possible.

In both the simulated and experimental data, the lactate concentration follows a similar pattern to the glucose concentration but with a slightly delayed peak. The simulated peak concentrations match the experimental data well in both magnitude and time relative to the peak in glucose concentration. However, the simulated concentration only falls to 0.82mM between meals in the model, compared with 0.52±0.15mM experimentally.

The triglyceride and glycerol concentrations show stronger periodic behaviour in the model simulations than in the experimental data. This difference occurs because adipose tissue storage of triglycerides is not included in the model. Since the focus of the study is on hepatic metabolism a separate adipose compartment allowing adipose triglyceride storage was not included. However, hepatic uptake of triglycerides from plasma is slow, such that the hepatic triglyceride concentration is more strongly dependent on the longer term average plasma concentration than on short-term fluctuations. Once equilibrium is reached, less than a 5% variation is seen in hepatic triglyceride concentration throughout a meal cycle, and the majority of this results from variation in hepatic synthesis. Additionally, very little lipolysis occurs in hepatocytes such that variations in hepatic triglyceride concentration only very weakly feed back into the rest of hepatic metabolism. Therefore, in developing a model to study hepatic metabolism, it is more important that the longer term average triglyceride concentration remains in the experimentally measured range. From Fig 2D and Table 1 it can be seen that the simulated triglyceride concentration remains in the same range as the experimental data. It is also important that the experimentally measured changes in average triglyceride concentration under conditions of insulin resistance are reproduced in the simulated data as discussed in the ‘*Validation of the Simulated Data in Insulin Resistant Patients*’ section.


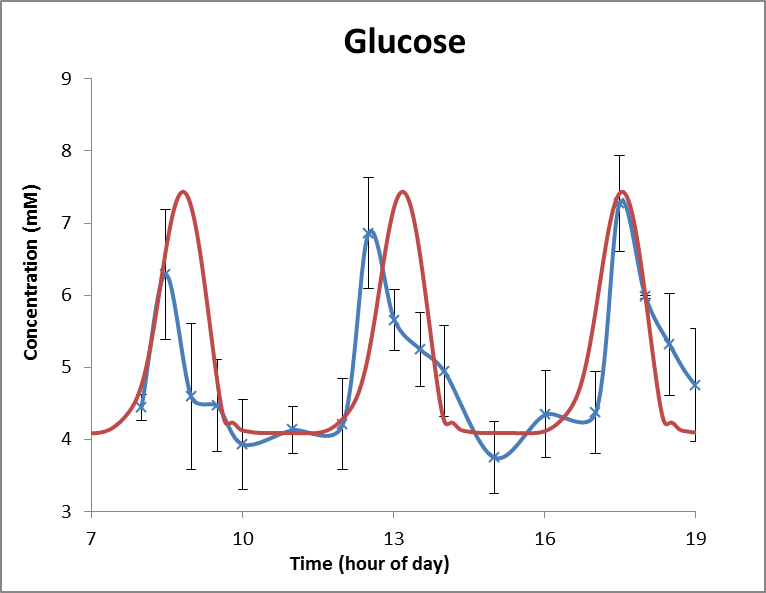

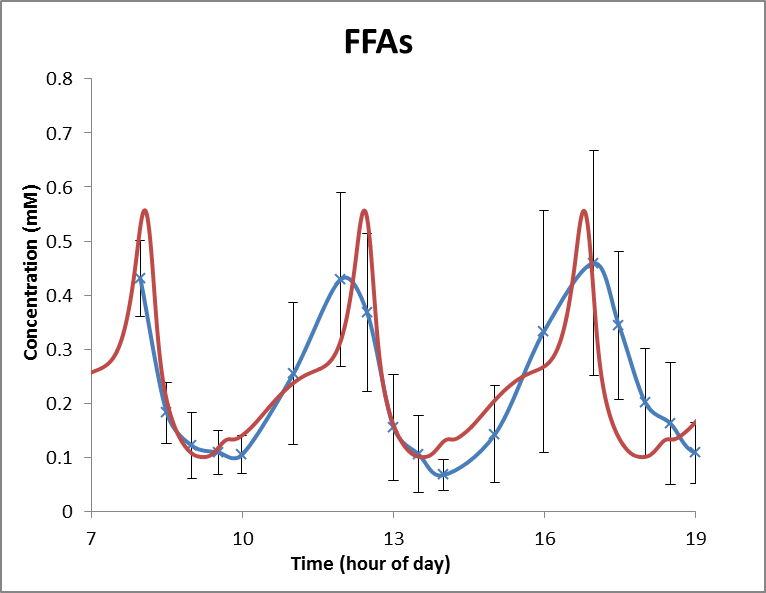

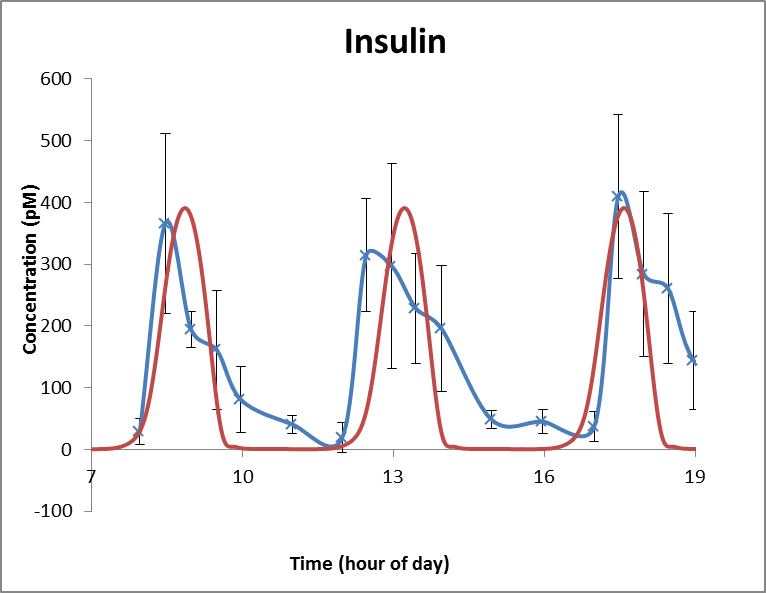

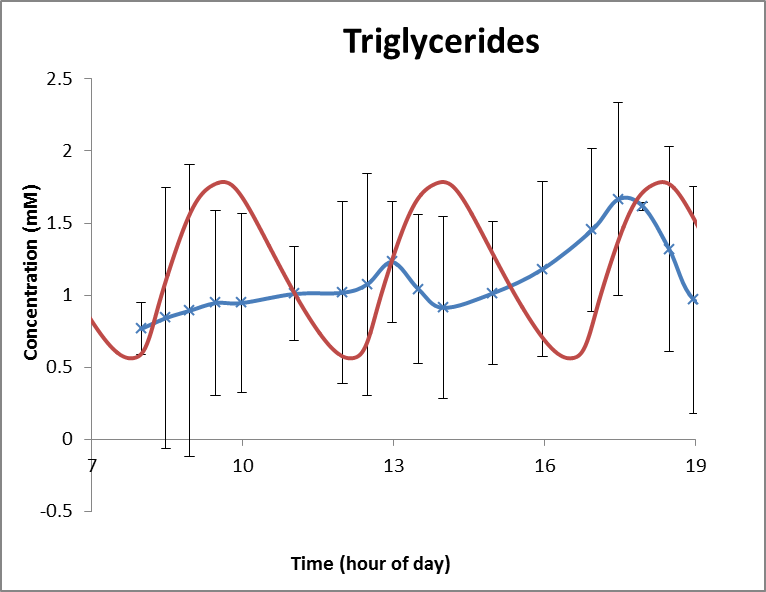


c)

d)

b)

a)

Fig 2. Comparison of the model simulations for (a) plasma glucose, (b) FFA, (c) insulin and (d) triglycerides throughout a daily feeding cycle with experimental data from Daly et al. [[17](#_ENREF_17)]. Experimental data approximated from the graphs provided using image J.

|  | Peak Concentrations | Trough Concentrations | Average Concentration | | Difference in peak time from peak glucose |
| --- | --- | --- | --- | --- | --- |
| Glucose | | | | | |
| Simulated | **7.43mM** | **4.08mM** | **4.92mM** | | - |
| Experimental | **Av.) 6.81±0.78mM**  **(Δ = 0.79 σ)**  1) 7.27±0.67mM  (0.24 σ)  2) 6.86±0.77mM  (0.74 σ)  3) 6.29±0.90mM  (1.09 σ) | **Av.) 3.84±0.56mM**  **(Δ = 0.43 σ)**  1) 3.75±0.50mM  (0.66 σ)  2) 3.93±0.62mM  (0.24 σ) | **4.59±0.65mM**  **(Δ = 0.51 σ)** | | - |
| FFAs | | | | | |
| Simulated | **557µM** | **100µM** | **229μM** | | **-45.6 mins** |
| Experimental | **Av.) 445±186µM**  **(Δ = 0.60 σ)**  1) 460 ± 208  (0.47 σ)  2) 429 ± 161  (.80 σ) | **Av.) 86±32µM**  **(Δ =0.44 σ)**  1)104±35  (0.11 σ)  2) 67±29  (1.14 σ) | **234±93μM**  **(Δ =0.05 σ)** | | **-30 mins**  **(-60<t<0 mins)** |
| Triglycerides | | | | | |
| Simulated | **1.79mM** | **0.56mM** | **1.19mM** | | **47.64 Minutes** |
| Experimental | **-No peaks or troughs following intake, but an increase from 0.77mM increase to 1.66mM throughout the day.** | | **1.08±0.30mM**  **(Δ =0.37 σ)** | | - |
| Lactate | | | | | |
| Simulated | **2.70 mM** | **0.82 mM** | | **1.23mM** | **19.7minutes** |
| Experimental | **Av.) 2.29** **± 0.92 mM**  **(Δ =0.45σ)**  2.11 ± 0.63mM  2.39 ± 1.20mM  2.38 ± 0.93mM | **Av.) 0.52 ± 0.15mM**  **(Δ =2σ)**  0.52±0.17 mM  0.52±0.13 mM | | **1.21 ± 0.55mM**  **(Δ =0.04 σ)** | **30 mins**  **(0<t<60 mins)** |
| Insulin | | | | | |
| Simulated | **391pM** | **1pM** | **89pM** | | **2.3 mins** |
| Experimental | **Av.) 363 ± 123 pM**  **(Δ =0.23σ)**  366 ± 146 pM  315 ± 91 pM  409 ± 133 pM | **Av.) 29±24pM**  **(Δ =1.17σ)**  19±24pM  38±24pM | **118 ± 90pM**  **(Δ = 0.32 σ)** | | **0 mins**  **(-30<t<30 mins)** |
| Glycerol | | | | | |
| Simulated | **39µM** | **21µM** | **30 μM** | | **2.0 hours** |
| Experimental | **39±19µM **Not periodic with meal cycle** | **22±10µM ** Not periodic with meal cycle** | **32±15 μM**  **(Δ =.13 σ)** | | **3.5±0.5, 4.5±0.5 hours** |

Table 8. Comparison of the simulated peak, trough and average concentrations and peak times relative to the glucose peak for plasma glucose, FFA, lactate, insulin, triglycerides and glycerol throughout a daily feeding cycle with experimental data from Daly et al. [[17](#_ENREF_17)]. Experimental data approximated from the graphs provided using image J.

### Validation of Zonation

#### ATP across sinusoid

Data measuring the concentrations of molecules in specific regions of the sinusoid are relatively few. As a result, hepatic heterogeneity in the model was largely validated against measurements of the relative rates of various processes occurring in different regions of the sinusoid. However, Nauck *et al.* approximated the change in ATP concentration across the sinusoid in an *in vitro* experiment by comparing cells grown in culture conditions similar those seen by periportal and pericentral cells *in vivo* [[16](#_ENREF_16)]. Cells cultured in periportal oxygen and hormone concentrations had an ATP concentration of 2.9±0.2mM whilst those grown in pericentral conditions had a concentration of 2.5±0.2mM [[16](#_ENREF_16)].

When simulating a moderate diet in a metabolically normal individual, the ATP concentration fell from 3.0mM in the periportal half of the sinusoid to 2.6mM in the pericentral side. Therefore, as well as the average values matching those measured *in vivo* [[1](#_ENREF_1)], the change in ATP concentration across the sinusoid is consistent with that measured between isolated periportal and pericentral cells [[16](#_ENREF_16)]. It is important that the energetics are represented properly since the inability of pericentral cells to produce sufficient ATP through oxidative phosphorylation is the primary motivation for zonation. Unfortunately data was not available for the relative contribution of oxidative phosphorylation and glycolysis across the sinusoid. However the relative rates of gluconeogenesis and glycolysis across the sinusoid are compared with experimental data below.

#### Processes

Although the zonation of enzymes in the model was based upon experimental data, the relative rates of processes across the sinusoid are also determined by other factors such as substrate concentrations and allosteric regulation. In this section, the relative rates of some of the key processes in the model are compared with experimental data to validate the representation of zonation in the model. In each case, model values are the average when the model was provided with an input cycle representing a moderate diet as discussed in S1 Text – ‘*Input*’ section.

##### β-oxidation

In two studies, Guzman *et al.* measured the rate of β-oxidation to be 1.2±0.3, and 1.4±0.3 times higher than that in pericentral cells in fed animals [[17-19](#_ENREF_17)]. Consistent with these studies, the simulated rate of β-oxidation is 1.25 times higher in the periportal half of the sinusoid than the periportal half. The simulated rate is 1.47 times higher in the proximal periportal compartment (compartment 1) compared to the distal pericentral compartment (compartment 8).

##### Triglyceride synthesis, storage and release as very low density lipoproteins (VLDL)

In two different *in vivo* studies by Guzman *et al.*, pericentral cells were measured to synthesize triglycerides at 1.6±0.4 and 1.3±0.3 times the rate of periportal cells, and release triglycerides as VLDL at 1.5±0.5 and 1.3±0.3 times the periportal rate [[19](#_ENREF_19)] [[17](#_ENREF_17)]. In the model, triglycerides are synthesized in pericentral cells at 1.64 times the rate in periportal cells.

Guzman *et al.* also measured an 11% increase in triglyceride concentration between periportal and pericentral hepatocytes, although this was not statistically significant [[17](#_ENREF_17)]. In the model, the average triglyceride concentration in the pericentral half of the sinusoid is 22% larger than in the periportal half, slightly overestimating this difference. However, this is strongly dependent on the glucose and FFA inputs. A higher glucose to FFA ratio gives more evenly distributed triglyceride levels.

##### Fatty acid uptake

Using fluorescently tagged fatty acids Fitz *et al.* measured higher fatty acid uptake in periportal cells [[20](#_ENREF_20)]. However no quantitative comparison was made. When simulated in the model, fatty acid uptake in the periportal half of the sinusoid was 1.21 times as higher than in pericentral cells.

##### Lipogenesis

When measured *in vivo* by Guzman *et al.* in two studies, the pericentral rate of lipogenesis was measured to be 1.6±0.4 and 1.7±0.5 times that in periportal cells [[17](#_ENREF_17), [19](#_ENREF_19)]. When simulated in the model, the pericentral rate of lipogenesis to be 1.9 times the periportal rate. This is within one standard deviation of the experimental data but slightly higher than the average. However, the relative rates of lipogenesis across the sinusoid are dependent on the ratio of glucose to FFA input.

##### Gluconeogenesis and glycolysis

Wolfe *et al* performed an *in vitro* study in which they measured the relative rates of gluconeogenesis and glycolysis in cells cultured in periportal or pericentral oxygen and hormone concentrations [[21](#_ENREF_21)]. Their results show that glycolysis is 1.5-2.5 times more rapid in pericentral-like cells whilst gluconeogenesis is 1.5-2.5 times more rapid in periportal-like cells [[21](#_ENREF_21)]. When simulating a moderate diet in the model, glycolysis occurs roughly 1.7 times more rapidly in pericentral cells whilst gluconeogenesis occurs 2.3 times as quickly in periportal cells consistent with the *in vitro* data.

Both the simulated data and the results of this *in vitro* study are consistent with the idea of glucose-lactate cycling known to occur *in vivo* [[22](#_ENREF_22)]. In glucose-lactate cycling, hypoxic pericentral cells undergo glycolysis to supplement ATP production from oxidative phosphorylation. This produces lactate which cycles round the body and is converted back to glucose by the oxygen rich periportal cells. Due to their higher oxygen environment, periportal hepatocytes can produce sufficient ATP through oxidative phosphorylation to survive and to fuel the additional gluconeogenesis, largely using fatty acids.

## Validation of the Simulated Data in Insulin Resistant Patients

Having shown that the model is able to produce realistic data for the average concentrations of hepatic molecules, for plasma concentrations of key variables during a daily feeding cycle and after a mixed meal, for glycogen storage after a mixed meal, for the rates of key metabolic processes during periods of glycogen synthesis and breakdown and for the zonation of key metabolic processes in metabolically normal individuals, the model outputs were next compared with experimental data when simulating insulin resistance. Insulin resistance is simulated in the model by multiplying the detected insulin by a constant 0≤K_IR_<1 where K_IR_=0 would correspond to total insulin resistance and K_IR_=1 would correspond to normal insulin sensitivity.

### Average plasma values in metabolically healthy and insulin resistant individuals

Firstly, the average plasma values of triglycerides, glucose and FFAs are compared with experimental data when simulating metabolically normal and insulin resistant individuals. The simulated average plasma concentrations are dependent on the inputs provided and on the severity of IR simulated. To provide the data for Table 9, simulations were run with the moderate intake daily diet as discussed in S1 Text – ‘*Input*’ section. Developing and severe IR were simulated by reducing the detected insulin concentration to 5% and 1.5% respectively.

|  | Simulated | Experimental |
| --- | --- | --- |
| Average Triglyceride (mM) | | |
| Metabolically Normal | 1.2±0.4 | 0.9±0.4^a^ |
| Insulin Resistant | Developing: 2.1±0.8  Severe: 3.7±0.9 | 2.6±0.5^a^ |
| Average Glucose (mM) | | |
| Metabolically Normal | 4.9±1.1 | 4.7±0.7^a^, 5.3±1.5 (4.5 - 6.8) ^d^ |
| Insulin Resistant | Developing: 6.3±2.4  Severe: 8.1±3.9 | 9.0±2.2^a^  Low TG group (less severe): 6.3±0.5 ^c^  High TG group (more severe): 6.8±0.5 ^c^  ­ Mild NIDDM: 8.0±2.7 (6.7 - 10.9) ^d^  Severe NIDDM: 19.6±5.3 (16.9 - 23.7) ^d^ |
| Average FFA (mM) | | |
| Metabolically Normal | 0.23±0.12 | 0.3±0.1^b^, 0.3±0.1 (0.16 - 0.52) ^d^ |
| Insulin Resistant | Developing: 0.48±0.16  Severe: 0.84±0.70 | 0.6±0.1^b^  Low TG group (less severe): 0.54±0.07 ^c^  High TG group (more severe): 0.81±0.07 ^c^  Mild T2DM: 0.4±0.1 (0.19 - 0.59) ^d^  Severe T2DM: 0.6±0.1 (0.37 - 0.75) ^d^ |

Table 9. The simulated and experimentally measured average triglyceride, glucose and FFA concentrations in metabolically normal and insulin resistant patients. Experimental data from: ^a^ – Sindelka et al. [[23](#_ENREF_23)], ^b^ – Berndt et al. [[24](#_ENREF_24)], ^c^ – Monti et al. [[25](#_ENREF_25)], ^d^ – Reavan et al. [[26](#_ENREF_26)] (^a,b^ standard deviations calculated based on the SEM provided, ^d^ – averages, ranges and error bars taken from the graphs provided using image J). Model values are averages ± the average deviation from the mean value over each feeding cycle.

For metabolically healthy individuals, the simulated data are within one standard deviation of the experimental data for glucose, FFAs and triglycerides further validating the ability of the model to give realistic data for non-insulin resistant patients (Table 9).

The FFA and triglycerides concentrations measured by Sindelka *et al.* [[23](#_ENREF_23)] and Berndt *et al.* [[24](#_ENREF_24)] in insulin resistant individuals lie between the simulated values for developing insulin resistance and for severe insulin resistance (Table 9). This is consistent with the experimental groups containing individuals with a range of severities of insulin resistance. Monti *et al.* measured FFA levels in T2DM individuals grouped according to their plasma triglyceride levels [[25](#_ENREF_25)]. The measured concentrations in these groups match the simulated data for developing and severe insulin resistance. Reavan et al meanwhile measured slightly lower average FFA concentrations in mild and severe T2DM than the simulated data [[26](#_ENREF_26)].

Large heterogeneity was seen in the experimental measurements of glucose concentrations in insulin resistant individuals. The mean concentration measured by Sindelka *et al.* in insulin resistant individuals is slightly higher than the simulated values when simulating severe insulin resistance (0.4 standard deviations) [[23](#_ENREF_23)]. Conversely the concentrations measured in both groups by Monti *et al.* are several standard deviations lower than the simulated concentration for severe insulin resistance. Meanwhile, the glucose concentration measured in mild T2DM by Reavan *et al*. matches that when simulating severe insulin resistance (0.03 standard deviations), whilst the concentration measured in severe T2DM is more than double this [[26](#_ENREF_26)].

This variation is likely to result from differences in the severity of insulin resistance within the group and differences in feeding. When simulating severely insulin resistant patients on a moderate diet, hyperglycaemia is seen post-prandially due to reduced glycogenesis. However, between meals the blood glucose concentration becomes hypoglycaemic because there are no glycogen stores to break down. It is because of this that the average glucose concentration to show only a modest increase. When simulating a high intake diet, sustained hyperglycaemia occurs in IR patients more consistent with the measurements of Reavan *et al*. [[26](#_ENREF_26)].

### Change in glucose and lactate concentrations after a glucose load

Having shown that the model provides reasonable values for average plasma concentrations in both metabolically normal and insulin resistant individuals, it was next compared with data for the change in plasma glucose and lactate concentrations after an oral glucose load. Table 10 shows the simulated and experimental data for the change in plasma lactate and glucose concentrations after intake of an 100g glucose load [[27](#_ENREF_27)]. Since the rate at which the oral glucose load glucose entered the blood stream in the experimental study is unknown, as an approximation, a spiked input of the form sin^6^(t), starting from zero input at t=0 and reaching peak input at 30 minutes was used. However, to account for the possibility that the sugars may enter the blood stream more or less rapidly than this, two additional input functions were used of the same form but with peak inputs at 20 minutes (rapid intake) and 45 minutes (slow intake). The simulated data in these two cases are the upper and lower error bars respectively. In all three cases, the total glucose input was equal to that in the experimental study (assuming 5L of blood in the body). Severe insulin resistance was simulated using an insulin resistance constant, K_IR_, of 0.015.

For both metabolically normal and insulin resistant individuals, the experimentally measured and simulated changes in glucose and lactate concentrations are well within one standard deviation of the experimental data. This shows that the model is able to accurately represent the changes in hepatic glycogenesis and glycolysis resulting from insulin resistance after a glucose load.

| Change in Glucose | Metabolically Normal | Insulin Resistant |
| --- | --- | --- |
| Simulated | ${2.1}_{-1.0}^{+1.0}$ | ${8.5}_{-3.6}^{+2.1}$ |
| Experimental | $2.8\pm2.0$ | $9.7\pm4.6$ |
| Change in Lactate | Metabolically Normal | Insulin Resistant |
| Simulated | ${0.46}_{-0.29}^{+1.50}$ | ${0.40}_{-0.12}^{+0.09}$ |
| Experimental | $0.46\pm0.12$ | $0.31\pm0.13$ |

Table 10. The simulated change in glucose concentration and lactate concentration after a 100g glucose load in metabolically normal and insulin resistant individuals compared with experimental data from Prando et al. [[27](#_ENREF_27)]. Experimental data approximated from the graph presented using Image J.

### Contribution of lipogenesis, FFA uptake and diet to hepatic triglycerides

| **Metabolically Healthy** | | |
| --- | --- | --- |
|  | Simulated | Experimental |
| Plasma FFA | 54.0% | - |
| Hepatic DNL | 12.0% | - |
| Dietary Triglycerides | 34.0% | - |
| **NAFLD** | | |
|  | Simulated (Liver & VLDL) | Experimental |
| Plasma FFAs | 60.0% | Liver: 59.0±9.9%  VLDL: 62.4±11.7% |
| Hepatic DNL | 20.2% | Liver: 26.1±6.7%  VLDL: 22.9±6.2% |
| Dietary Triglycerides | 19.8% | Liver: 14.9±7.0%  VLDL: 14.7±8.5% |

Table 11. The simulated contribution of hepatic de novo lipogenesis, FFA uptake and dietary triglycerides to hepatic steatosis compared with experimental data from Donnelly *et al.* [[28](#_ENREF_28)].

The contribution of plasma FFAs, hepatic *de novo* lipogenesis and uptake of dietary triglycerides to hepatic triglyceride levels when simulating NAFLD are compared with experimental data from Donnelly *et al*. in Table 11 [[28](#_ENREF_28)]. Additionally, the simulated contributions of the three sources of fatty acids to hepatic triglyceride levels are presented for metabolically healthy individuals. Severe insulin resistance (K_IR_=0.015) with increased SREBP-1c expression (1nM insulin stimulation of lipogenesis and triglyceride synthesis) was simulated to represent NAFLD. Donnelly *et al.* measured the contribution of these three sources to both liver triglycerides and VLDL. However, in the model, the composition of VLDL is only determined by the composition of liver triglycerides such that the two are equal.

The contributions of each source of fatty acids to both liver triglycerides and VLDL are all within one standard deviation of the values measured by Donnelly *et al*. [[28](#_ENREF_28)]. The contribution of dietary triglycerides is towards the high end of the measured values suggesting this may be slightly overestimated. However, this component is the most susceptible to changes in dietary input, and the simulated values remain in the correct range.

The simulated contribution of plasma FFAs was slightly lower for metabolically healthy individuals relative to NAFLD patients (54.0% vs 60.0%). The simulated contribution of dietary triglycerides was higher in metabolically normal individuals relative to NAFLD patients (34.0% vs 19.8%) whilst the percentage of hepatic triglycerides arising from *de novo* lipogenesis was lower (12.0% vs 20.2%).

## Validation of the relative contributions of lipids and carbohydrates to oxidative phosphorylation

In order to properly integrate hepatic glucose and lipid metabolisms, it is important that it is able to predict the rates at which fatty acids and glucose are oxidised under different conditions. Relatively little time series data exists investigating the rates of oxidation in liver. However, in the following sections the model simulations for the rates in liver are compared with experimental data for the overall rates in the body as a whole. Experimental and simulated rates are compared after a mixed meal in metabolically normal individuals (‘*Energy production after a mixed meal*’) and after the intake of starch with either slow or fast hydrolysis rates in metabolically normal and T2DM individuals (‘*Comparison of energy production in metabolically healthy and diabetic individuals*’).

### Energy production after a mixed meal

Daly *et al.* measured the rates of carbohydrate and fatty acid oxidation after a high sucrose/low starch meal with 65% of energy as carbohydrate and 26% as fat (2.5:1) [[29](#_ENREF_29)]. Using the number of ATP molecules produced per glucose and per palmitate in the model, this corresponds to 6.9 moles of glucose per mole of palmitate (or 4.85 grams of glucose per gram of palmitate).

The model was provided with glucose and FFA inputs to match this high sugar/low starch meal with a peak input at 30 minutes. The simulated FFA and glucose concentrations (Figs 3A, 3B) remain well within one standard deviation of the experimental data, although the simulated glucose concentration has a less sharp peak.

In the experimental study, the rates of carbohydrate and lipid oxidation were measured in grams per minute in the body as a whole (Figs 4A, 4C). These results cannot be compared quantitatively with the simulated data for the rate of pyruvate oxidation and β-oxidation in liver alone in moles per minute (Figs 4B, 4D). However, qualitative comparison of the changes occurring postprandially can be made.

The simulated curves for the rates of fatty acid and carbohydrate oxidation follow a similar shape over the 6 hours to those measured experimentally. The rate of carbohydrate oxidation doubles postprandially before falling back to its initial value in both the simulated data and experimental data. Similarly, in both cases the rate of fatty acid oxidation falls by half before rising to slightly above its initial value.

However, the peak in carbohydrate oxidation and the trough in lipid oxidation appear sharper and more rapidly in the simulated data than experimentally. This may be as a result of the fact that only the liver is simulated, rather than the body as a whole. Due to the role of liver in the storage of glucose, and the large quantities of blood that pass through the organ, hepatocytes would be expected to react particularly quickly to changes in plasma insulin and glucagon levels. When considering the body as a whole, less responsive organs would slow this effect.


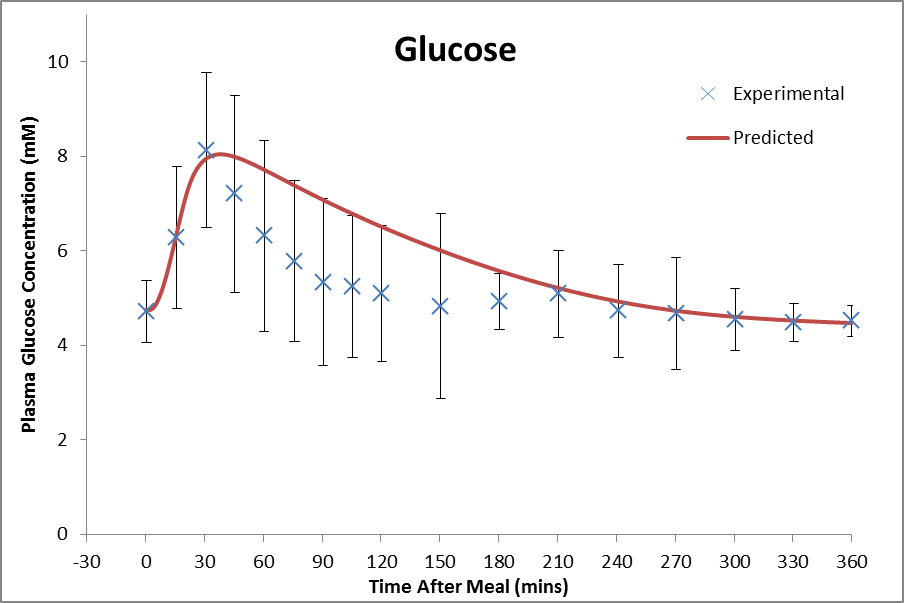

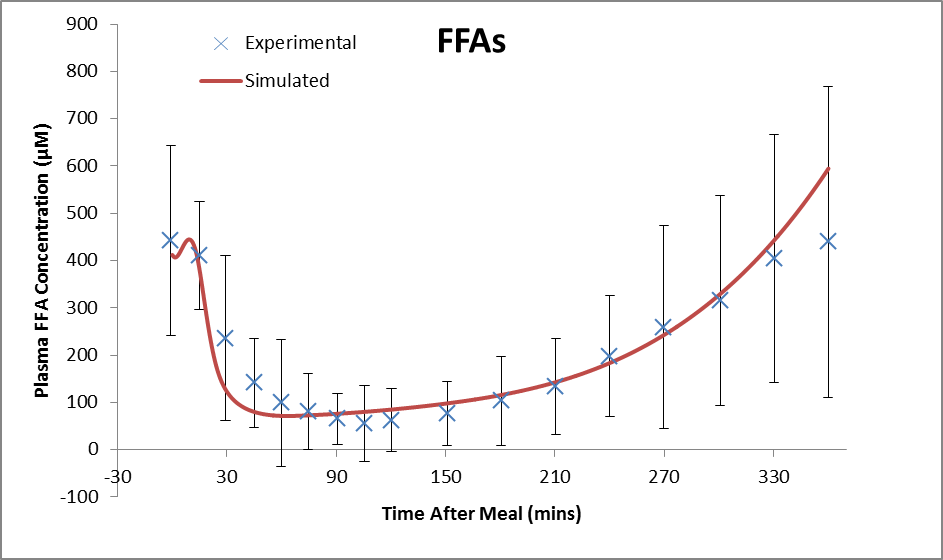


Fig 3. Comparison of the simulated (a) plasma glucose and (b) fatty acid concentration after intake of a mixed meal with data measured experimentally by Daly et al. [[29](#_ENREF_29)].

b)

a)


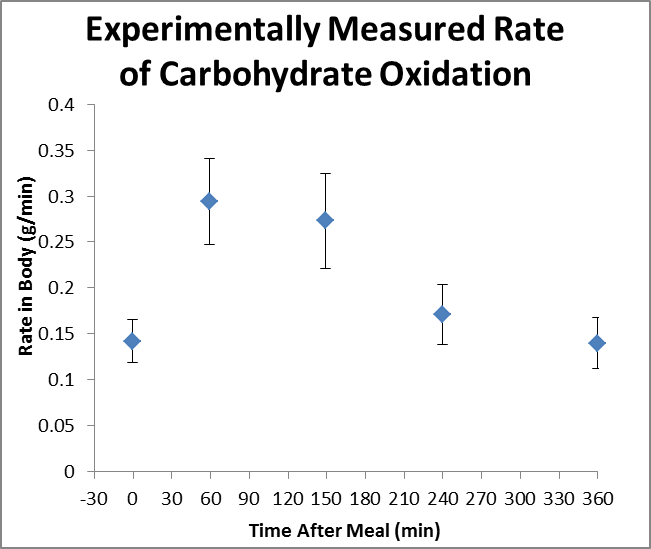

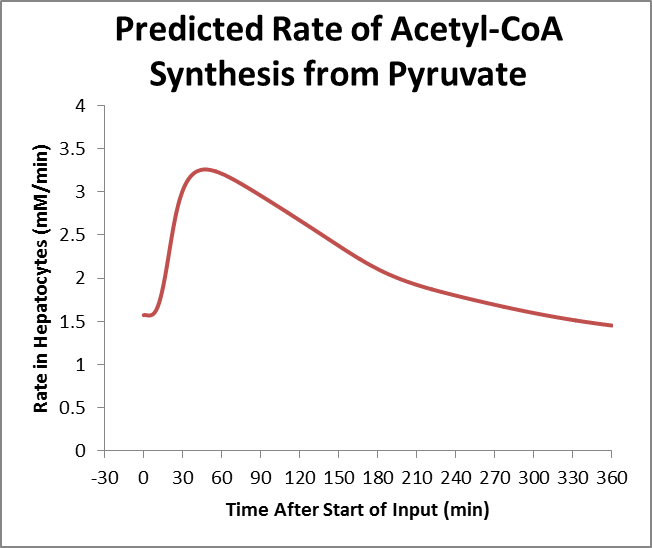


b)

a)


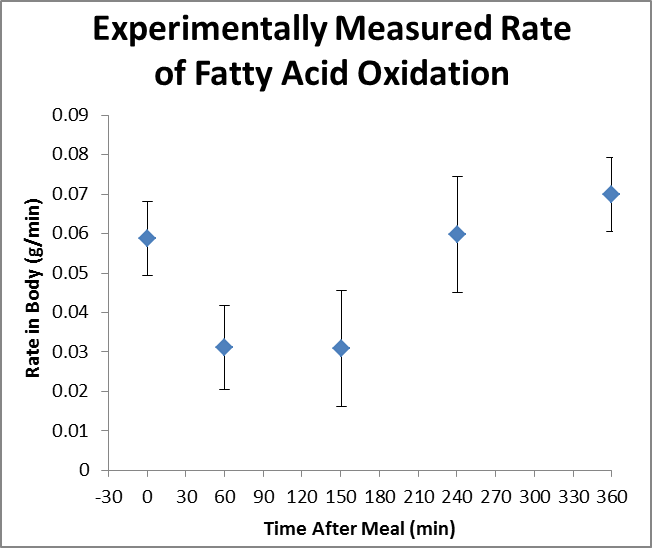

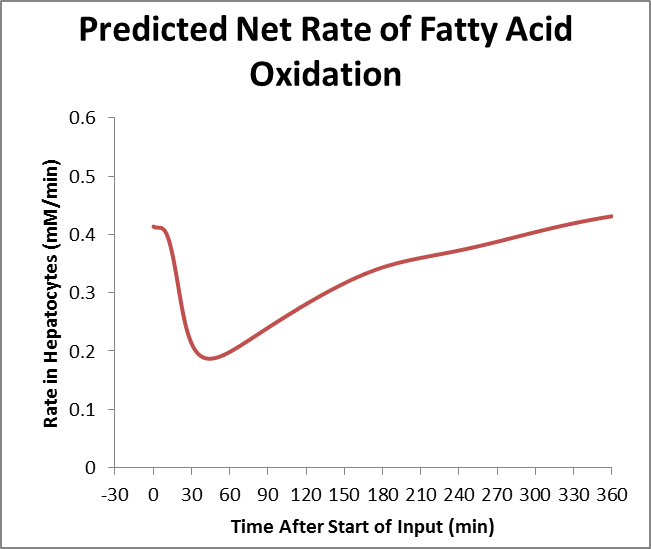


c)

d)

Fig 4. The rates of oxidation of (a,b) glucose and (c,d) fatty acids and when (a,c) measured experimentally in the body as a whole by Daly et al. [[29](#_ENREF_29)] and (b,d) simulated in liver alone after intake of a mixed meal as discussed in the text.

### Comparison of energy production in metabolically healthy and diabetic individuals

Seal *et al.* compared the rates of carbohydrate and lipid oxidation in metabolically healthy and T2DM patients fed 50g of rapidly or slowly hydrolysed starch [[30](#_ENREF_30)]. In this study, the rates at which the two starches were broken down to sugars were also measured, giving an idea of the rate at which glucose entered the blood stream. To validate the models simulations for energy metabolism in insulin resistant individuals, the model was next provided with glucose inputs set to match the rates at which carbohydrates were broken down *in vivo*. For the fast release starch, an input was provided with a sharp peak at around 30 minutes before falling rapidly to zero. For slow release starch an input with a smaller peak centered after an hour was used before falling more slowly, such that the rate of input remained at 17% of its peak rate after 6 hours (as measured experimentally). Experimentally, only 70% of the slowly hydrolysed starch was broken down over the 6 hours compared to 96% for the rapidly hydrolysed starch. As a result, the total glucose inputted to the model was slightly larger when simulating the rapidly hydrolysed starch. In both cases, the model was run for 1.5 hours with no input before the starch intake to allow variables to equilibrate.

Insulin resistance was simulated by multiplying the detected insulin concentration by a constant (K_IR_) smaller than one. Three severities of insulin resistance were simulated to account for variability in the degree of insulin sensitivity between T2DM individuals. Mild, moderate and severe insulin resistance correspond to 90%, 95% and 98.5% de-sensitivity respectively in the graphs.

For the metabolically healthy individuals, the simulated glucose concentrations match the experimental data fairly accurately with the average difference between the simulated and experimental curves well within one standard deviation. This provides further validation that the model simulations for the plasma glucose concentration under conditions where the rate of input is known are consistent with experimental data (Fig 5).

The simulated data for the glucose concentration in insulin resistant individuals match the experimental data through the initial postprandial rise and initial subsequent fall (Fig 5). The experimentally measured concentration remains within one standard deviation of the simulated data for moderate insulin resistance and between the simulated data for mild and severe insulin resistance over the 4-5 hours. However, around 4 hours after the intake of fast release starch and 5 hours after the intake of slow release starch, a drop of glucose concentration to well below the pre-prandial value was measured experimentally but not seen in the simulated data. This was, accompanied by a widening of the error bars in the experimental data. When simulating insulin resistance, the only change that was made was to multiply the detected insulin concentration by an insulin resistance constant less than 1 to allow direct comparison. However, *in vivo*, diabetics additionally show severely depleted glycogen stores reducing their capacity to provide glucose to the blood when plasma concentrations fall. Therefore, the difference between model predictions and experimental data is likely to arise as a result of the fact that in simulations the initial glycogen stores were set to 200mM for both insulin resistant and metabolically normal individuals. Due to this difference in plasma concentrations after 4-5 hours, the rates of oxidation are only compared for the first 300 minutes.

As with the previous section, the model simulates the rates of pyruvate oxidation and fatty acids in liver and so cannot be compared quantitatively with the experimentally measured rates of carbohydrate and lipid oxidation in the body as a whole (Figs 6 and 7). However, the change in the rates of these processes occurring postprandially in the liver should be qualitatively similar to those for the body as a whole.

Over the 360 minutes, the changes in the rates of oxidation relative to baseline match the experimental data closely with a upregulation of carbohydrate oxidation and reduction in lipid oxidation occurring. In the case of fast release starch, a slightly larger increase in carbohydrate oxidation and reduction in lipid oxidation is seen in T2DM than metabolically healthy individuals after the intake of fast release starch in both the simulated and experimental data (Fig 6). In the case of slow release starch the change in the rate of fatty acid oxidation was roughly the same for T2DM and insulin sensitive individuals in both the simulated and experimental data (Fig 7). A slightly lower increase in carbohydrate oxidation was seen for T2DM individuals experimentally, perhaps due to depleted glycogen stores.

Glucose Concentrations


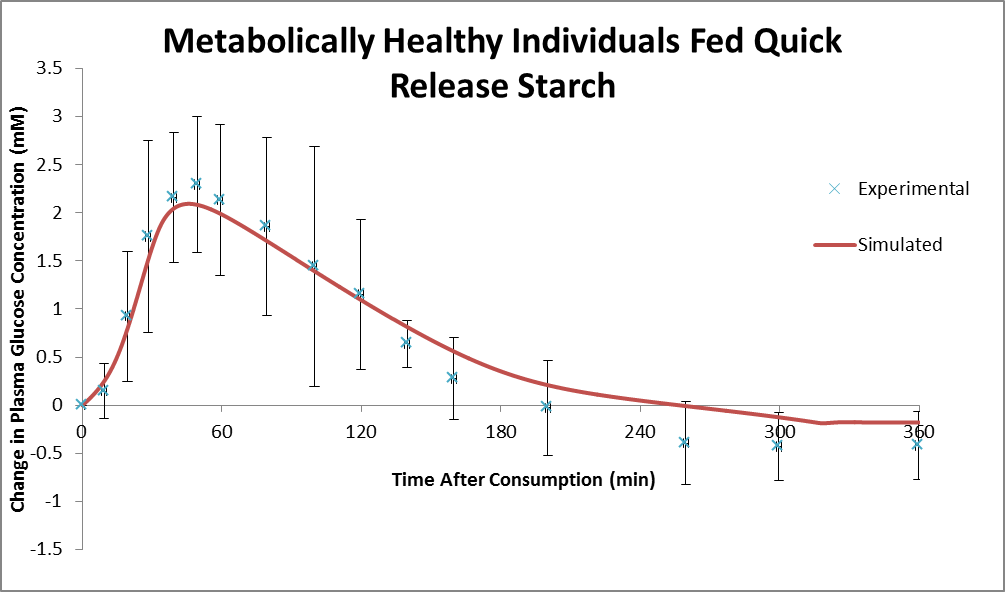

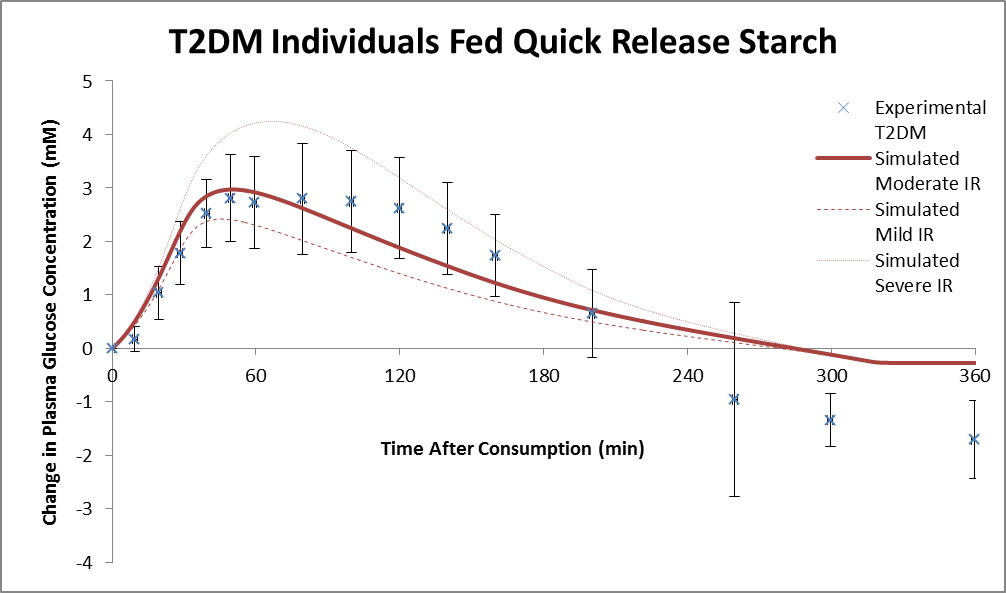

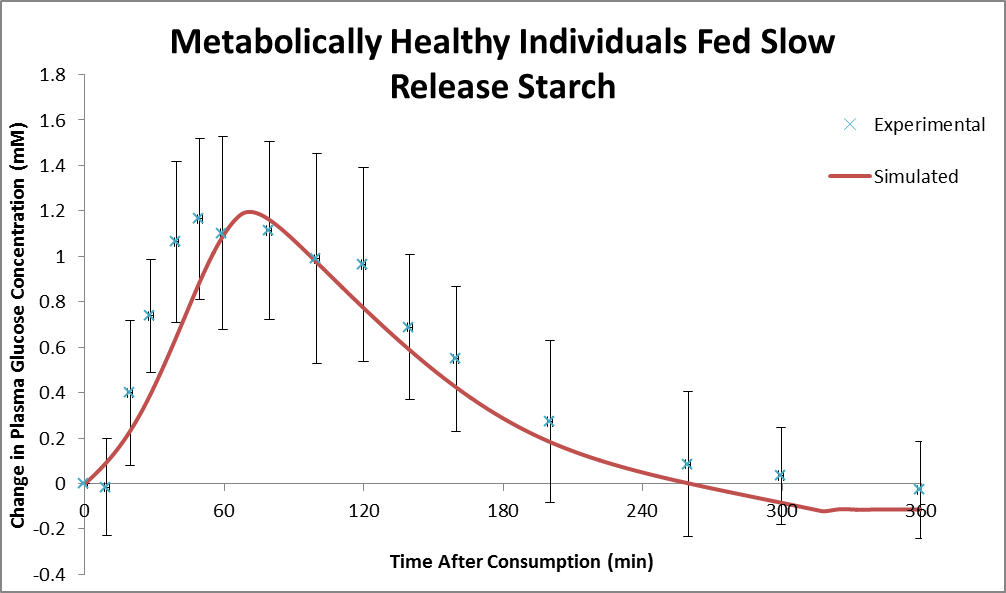

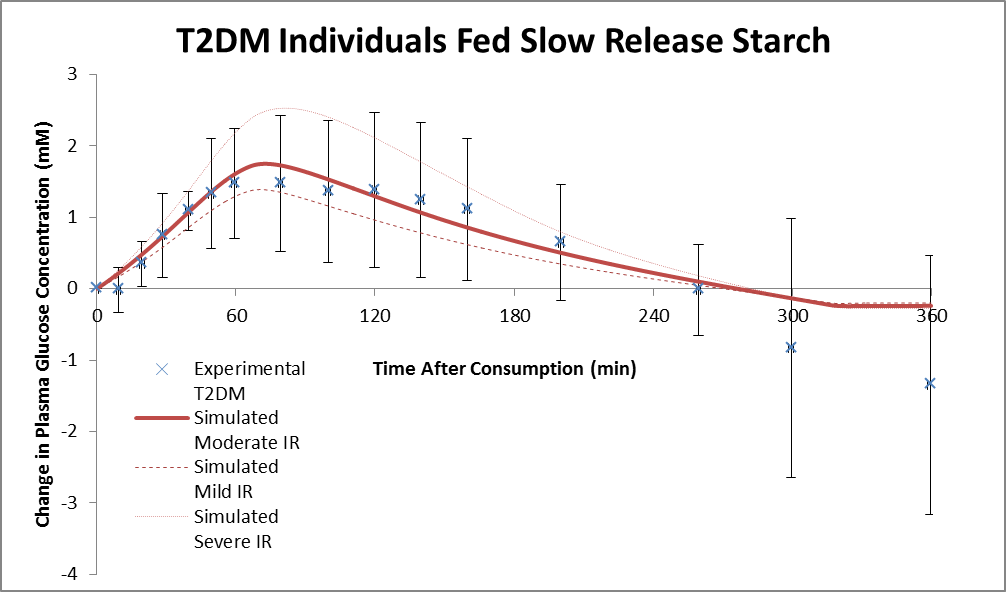


Fig 5. The glucose concentration after intake of a (top) 50g of quick release starch and (bottom) 50g of slow release starch when measured experimentally by Seal et al. [[30](#_ENREF_30)] and simulated by the model in (left) metabolically healthy and (right) T2DM individuals.

Quick Release Starch


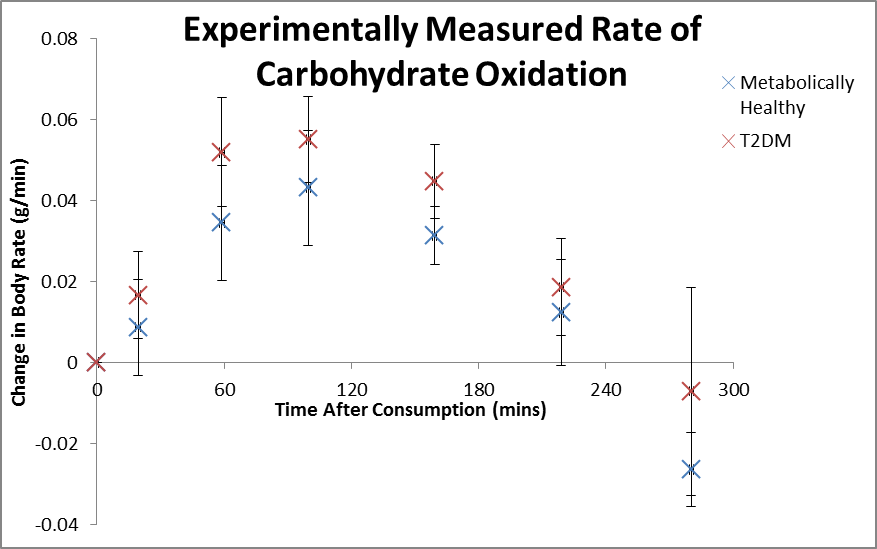

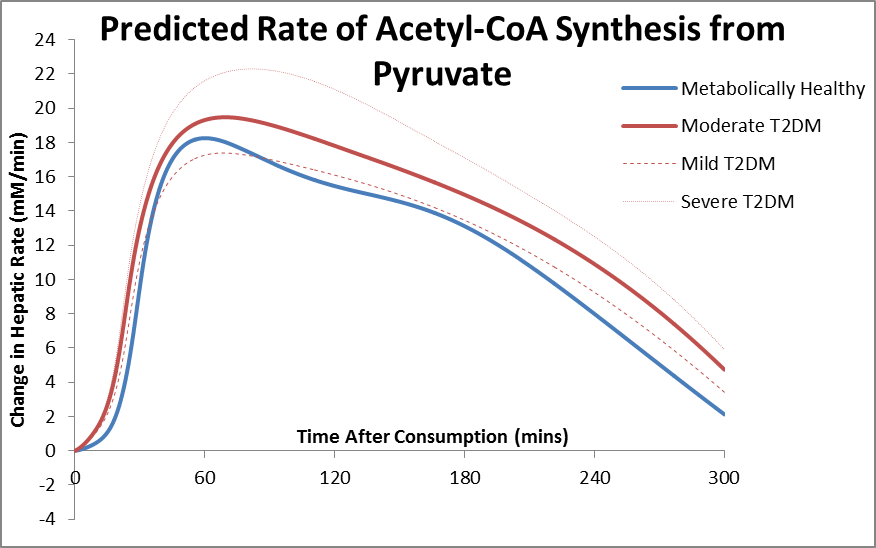

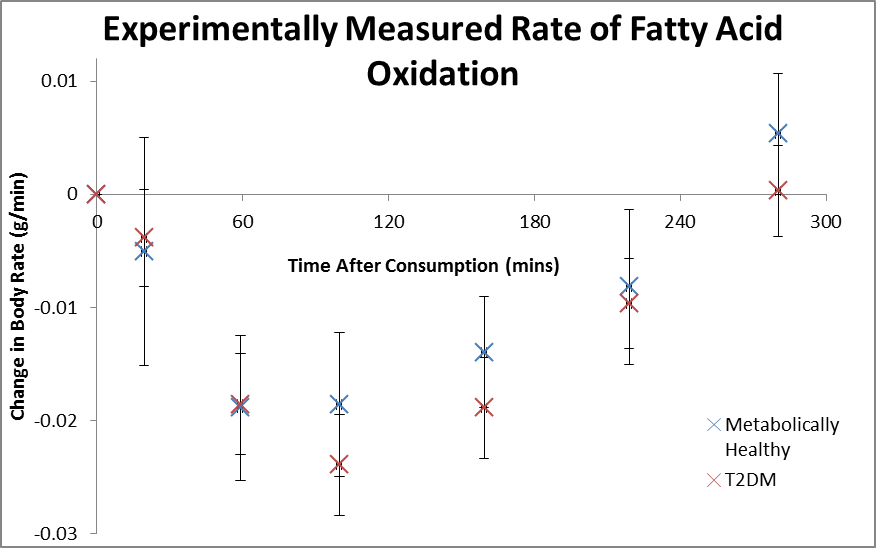

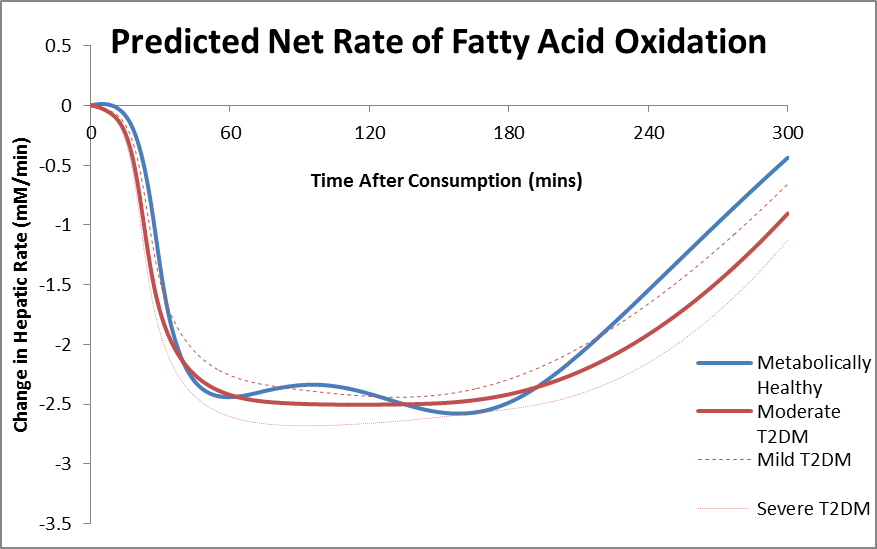


Fig 6. The rates of oxidation of fatty acids and glucose in metabolically healthy and T2DM individuals when (left) measured experimentally in the body as a whole by Seal et al. [[30](#_ENREF_30)] and (right) when simulated in liver alone after intake of 50g of quick release starch as discussed in the text.

Slow Release Starch


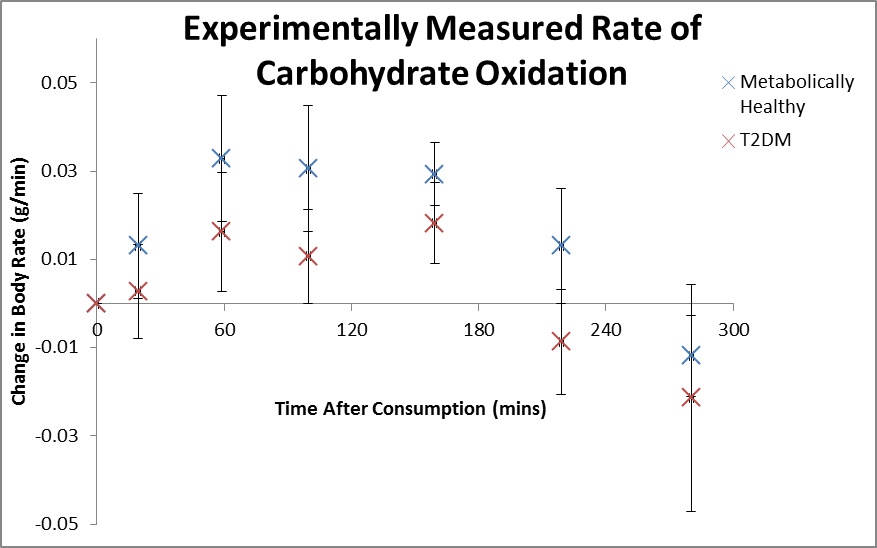

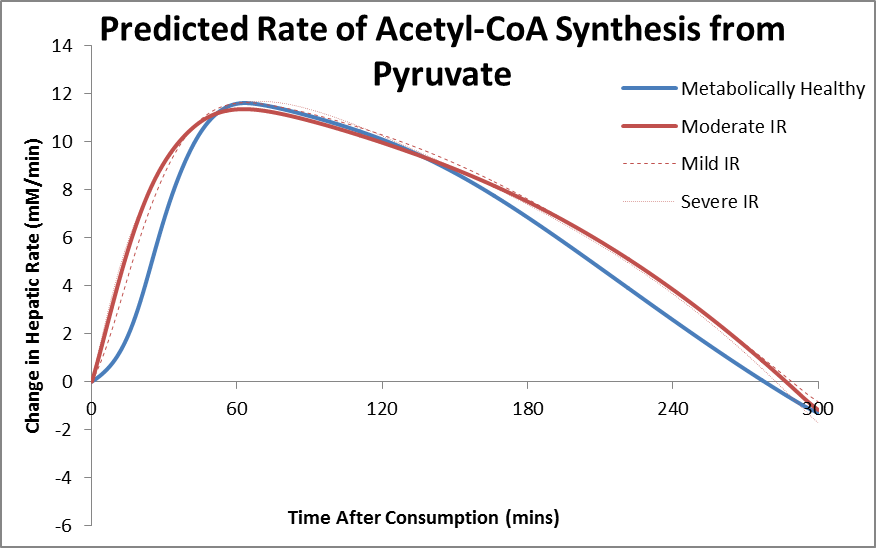

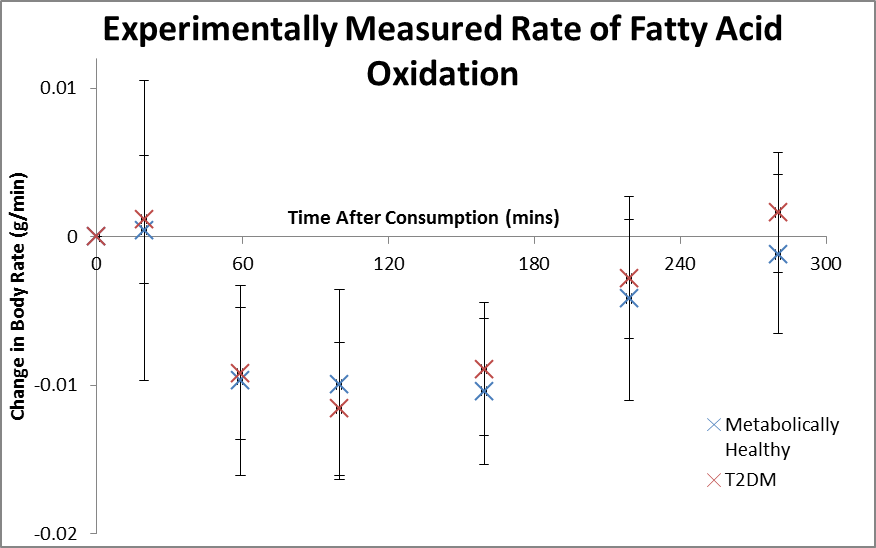

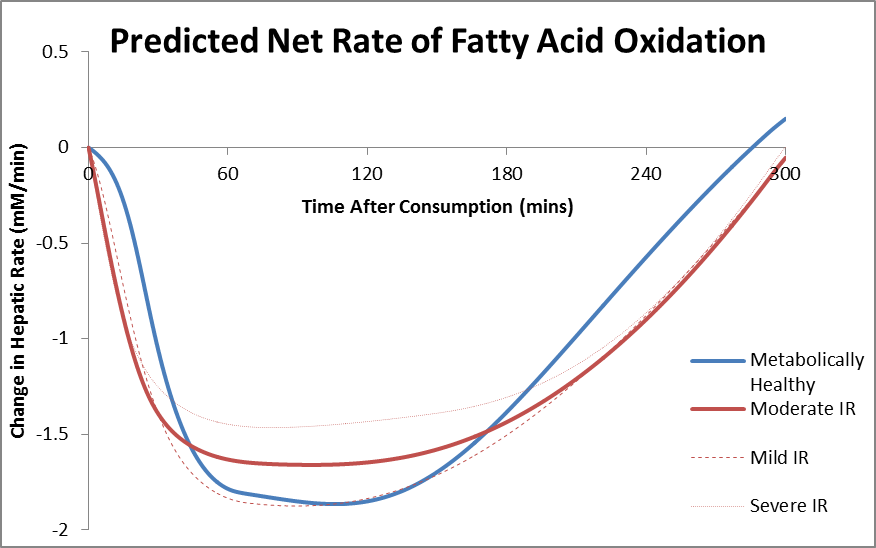


Fig 7. The rates of oxidation of fatty acids and glucose in metabolically healthy and T2DM individuals when (left) measured experimentally in the body as a whole by Seal et al. [[30](#_ENREF_30)] and (right) when simulated in liver alone after intake of 50g of slow release starch as discussed in the text. Error bars in experimental data are SEM rather than SD with n=8 for metabolically healthy and n=13 for T2DM.

## Supplementary Material 2 References

1. Veech, R.L., D. Veloso, and M.A. Mehlman, *Thiamin deficiency: liver metabolite levels and redox and phosphorylation states in thiamin-deficient rats.* J Nutr, 1973. **103**(2): p. 267-72.

2. Jackson, R.C., H.P. Morris, and G. Weber, *Partial purification, properties and regulation of inosine 5'phosphate dehydrogenase in normal and malignant rat tissues.* Biochem J, 1977. **166**(1): p. 1-10.

3. Rawat, A.K., *Effects of ethanol infusion on the redox state and metabolite levels in rat liver in vivo.* Eur J Biochem, 1968. **6**(4): p. 585-92.

4. Albe, K.R., M.H. Butler, and B.E. Wright, *Cellular Concentrations of Enzymes and Their Substrates.* Journal of Theoretical Biology, 1990. **143**(2): p. 163-195.

5. Williamson DH, B.J., Concentrations of metabolites in animal tissues. In: Methods of enzymatic analysis (Bergmeyer HV ed.) pp. 2266-2302. New York: Academic Press.

6. Taylor, R., et al., *Direct assessment of liver glycogen storage by 13C nuclear magnetic resonance spectroscopy and regulation of glucose homeostasis after a mixed meal in normal subjects.* J Clin Invest, 1996. **97**(1): p. 126-32.

7. Szczepaniak, L.S., et al., *Magnetic resonance spectroscopy to measure hepatic triglyceride content: prevalence of hepatic steatosis in the general population.* Am J Physiol Endocrinol Metab, 2005. **288**(2): p. E462-8.

8. Guzman, M. and J. Castro, *Zonal Heterogeneity of the Effects of Chronic Ethanol Feeding on Hepatic Fatty-Acid Metabolism.* Hepatology, 1990. **12**(5): p. 1098-1105.

9. Faupel, R.P., et al., *The problem of tissue sampling from experimental animals with respect to freezing technique, anoxia, stress and narcosis. A new method for sampling rat liver tissue and the physiological values of glycolytic intermediates and related compounds.* Arch Biochem Biophys, 1972. **148**(2): p. 509-22.

10. Saggerson, E.D. and A.L. Greenbaum, *The regulation of triglyceride synthesis and fatty acid synthesis in rat epididymal adipose tissue.* Biochem J, 1970. **119**(2): p. 193-219.

11. Jungermann, K. and N. Katz, *Functional specialization of different hepatocyte populations.* Physiol Rev, 1989. **69**(3): p. 708-64.

12. Ainscow, E.K. and M.D. Brand, *Top-down control analysis of ATP turnover, glycolysis and oxidative phosphorylation in rat hepatocytes.* Eur J Biochem, 1999. **263**(3): p. 671-85.

13. Edgerton, D.S., et al., *Effects of insulin on the metabolic control of hepatic gluconeogenesis in vivo.* Diabetes, 2009. **58**(12): p. 2766-75.

14. Mandarino, L.J., et al., *Effects of insulin infusion on human skeletal muscle pyruvate dehydrogenase, phosphofructokinase, and glycogen synthase. Evidence for their role in oxidative and nonoxidative glucose metabolism.* J Clin Invest, 1987. **80**(3): p. 655-63.

15. Gamberucci, A., et al., *Low levels of glucose-6-phosphate hydrolysis in the sarcoplasmic reticulum of skeletal muscle: involvement of glucose-6-phosphatase.* Mol Membr Biol, 1996. **13**(2): p. 103-8.

16. Nauck, M., et al., *Modulation of the glucagon-dependent induction of phosphoenolpyruvate carboxykinase and tyrosine aminotransferase by arterial and venous oxygen concentrations in hepatocyte cultures.* Eur J Biochem, 1981. **119**(3): p. 657-61.

17. Guzman, M. and J. Castro, *Zonal heterogeneity of the effects of chronic ethanol feeding on hepatic fatty acid metabolism.* Hepatology, 1990. **12**(5): p. 1098-105.

18. Guzman, M., C. Bijleveld, and M.J. Geelen, *Flexibility of zonation of fatty acid oxidation in rat liver.* Biochem J, 1995. **311 ( Pt 3)**: p. 853-60.

19. Guzman, M. and J. Castro, *Zonation of fatty acid metabolism in rat liver.* Biochem J, 1989. **264**(1): p. 107-13.

20. Fitz, J.G., N.M. Bass, and R.A. Weisiger, *Hepatic transport of a fluorescent stearate derivative: electrochemical driving forces in intact rat liver.* Am J Physiol, 1991. **261**(1 Pt 1): p. G83-91.

21. Wolfle, D. and K. Jungermann, *Long-term effects of physiological oxygen concentrations on glycolysis and gluconeogenesis in hepatocyte cultures.* Eur J Biochem, 1985. **151**(2): p. 299-303.

22. Jungermann, K. and T. Kietzmann, *Role of oxygen in the zonation of carbohydrate metabolism and gene expression in liver.* Kidney Int, 1997. **51**(2): p. 402-12.

23. Sindelka, G., et al., *Association of obesity, diabetes, serum lipids and blood pressure regulates insulin action.* Physiol Res, 2002. **51**(1): p. 85-91.

24. Berndt, J., et al., *Fatty acid synthase gene expression in human adipose tissue: association with obesity and type 2 diabetes.* Diabetologia, 2007. **50**(7): p. 1472-80.

25. Monti, L.D., et al., *Myocardial insulin resistance associated with chronic hypertriglyceridemia and increased FFA levels in Type 2 diabetic patients.* Am J Physiol Heart Circ Physiol, 2004. **287**(3): p. H1225-31.

26. Reaven, G.M., et al., *Measurement of plasma glucose, free fatty acid, lactate, and insulin for 24 h in patients with NIDDM.* Diabetes, 1988. **37**(8): p. 1020-4.

27. Prando, R., et al., *Blood Lactate Behavior after Glucose-Load in Diabetes-Mellitus.* Acta Diabetologica Latina, 1988. **25**(3): p. 247-256.

28. Donnelly, K.L., et al., *Sources of fatty acids stored in liver and secreted via lipoproteins in patients with nonalcoholic fatty liver disease.* J Clin Invest, 2005. **115**(5): p. 1343-51.

29. Daly, M.E., et al., *Acute fuel selection in response to high-sucrose and high-starch meals in healthy men.* Am J Clin Nutr, 2000. **71**(6): p. 1516-24.

30. Seal, C.J., et al., *Postprandial carbohydrate metabolism in healthy subjects and those with type 2 diabetes fed starches with slow and rapid hydrolysis rates determined in vitro.* Br J Nutr, 2003. **90**(5): p. 853-64.
